# Supplementation with fortified balanced energy–protein during pregnancy and lactation and its effects on birth outcomes and infant growth in southern Nepal: protocol of a 2×2 factorial randomised trial

**DOI:** 10.1136/bmjpo-2023-002229

**Published:** 2023-11-03

**Authors:** Daniel J Erchick, Tsering P Lama, Subarna K Khatry, Joanne Katz, Luke C Mullany, Eleonor Zavala, Steven C LeClerq, Parul Christian, James M Tielsch

**Affiliations:** 1 Department of International Health, Johns Hopkins Bloomberg School of Public Health, Baltimore, Maryland, USA; 2 Nepal Nutrition Intervention Project Sarlahi (NNIPS), Kathmandu, Nepal; 3 Department of Global Health, Milken Institute School of Public Health, George Washington University, Washington, District of Columbia, USA

**Keywords:** Epidemiology, Infant

## Abstract

**Introduction:**

Many women in low and middle-income countries enter pregnancy with low nutritional reserves with increased risk of fetal growth restriction and poor birth outcomes, including small-for-gestational-age (SGA) and preterm birth. Balanced energy–protein (BEP) supplements have shown reductions in risk of stillbirth and SGA, yet variations in intervention format and composition and limited evidence on the impact of BEP during lactation on growth outcomes warrant further study. This paper describes the protocol of the Maternal Infant Nutrition Trial (MINT) Study, which aims to evaluate the impact of a fortified BEP supplement during pregnancy and lactation on birth outcomes and infant growth in rural Nepal.

**Methods and analysis:**

MINT is a 2×2 factorial, household randomised, unblinded, efficacy trial conducted in a subarea of Sarlahi District, Nepal. The study area covers six rural municipalities with about 27 000 households and a population of approximately 100 000. Married women (15–30 years) who become pregnant are eligible for participation in the trial and are randomly assigned at enrolment to supplementation with fortified BEP or not and at birth to fortified BEP supplementation or not until 6 months post partum. The primary pregnancy outcome is incidence of SGA, using the INTERGROWTH-21st standard, among live born infants with birth weight measured within 72 hours of delivery. The primary infant growth outcome is mean length-for-age z-score at 6 months using the WHO international growth reference.

**Ethics and dissemination:**

The study was approved by the Institutional Review Board (IRB) at Johns Hopkins Bloomberg School of Public Health, Baltimore, USA (IRB00009714), the Committee on Human Research IRB at The George Washington University, Washington, DC, USA (081739), and the Ethical Review Board of the Nepal Health Research Council, Kathmandu, Nepal (174/2018).

**Trial registration number:**

NCT03668977.

**WHAT IS ALREADY KNOWN ON THIS TOPIC**

- Undernourished women are at increased risk of fetal growth restriction and adverse birth outcomes, including preterm birth and small-for-gestational age.
- The WHO recommendations on antenatal care include balanced energy–protein (BEP) supplementation in undernourished populations to reduce the risk of stillbirth and small-for-gestational-age birth.

**WHAT THIS STUDY ADDS**

- The Maternal Infant Nutrition Trial (MINT) will demonstrate whether fortified BEP reduces risk of adverse pregnancy outcomes beyond multiple micronutrient supplementation.
- This study will also generate evidence on whether maternal intervention prevents early life infant growth faltering and improves maternal outcomes, such as anaemia and inadequate gestational weight gain.

**HOW THIS STUDY MIGHT AFFECT RESEARCH, PRACTICE OR POLICY**

- MINT will help refine and implement the WHO context-specific recommendation on BEP supplementation in pregnancy.
- The trial will also provide evidence for potential intervention in pregnancy and post partum on infant growth outcomes.

## Introduction

Many women in low and middle-income countries (LMICs) enter pregnancy with low nutritional reserves, unable to meet the increased requirements for protein, energy, and micronutrients during pregnancy and lactation. Undernourished women are at increased risk of fetal growth restriction and adverse birth outcomes, including preterm birth (<37 gestational weeks) and small-for-gestational-age (SGA) (birth weight <10th percentile of a reference standard by gestational age and sex), two underlying biological causes of low birth weight (LBW) (<2.5 kg).^1^ SGA infants are more likely to experience higher mortality, linear growth faltering and poor health outcomes, poor cognitive performance, and diminished educational and employment attainment.^2–4^ In 2020, 23.4 million infants (17.4% of all births) were born SGA, with the highest prevalence in South Asia (40.9%).^5 6^ Estimates from 2012 indicate that 22% neonatal deaths in LMICs, and 26% in South Asia, were attributable to SGA.^7 8^

Infant size status at birth and growth in the first 6 months of life, especially among preterm and SGA infants, are critical determinants of childhood stunting and wasting associated with higher risk of mortality, morbidity and poor developmental outcomes.^9^ Many maternal risk factors associated with pregnancy outcomes and consequent preterm or SGA are likely contributors to linear growth failure.^10^ Postnatal factors, such as breastfeeding and feeding behaviours, infections and environmental factors, are also important drivers of infant growth.^11 12^

Nutritional interventions targeted to women during pregnancy and lactation provide an opportunity to break the intergenerational cycle of growth failure. The WHO recommendations on antenatal care (ANC) include balanced energy–protein (BEP) supplementation in undernourished populations to reduce the risk of stillbirth and SGA birth.^13^ This is based on a systematic review of BEP supplementation during pregnancy that has shown decreased risk of stillbirth (relative risk (RR): 0.60, 95% CI: 0.39 to 0.94) and SGA (RR: 0.79, 95% CI: 0.69 to 0.90).^14^ BEP also increased mean infant birth weight by 41 g overall and 100 g among undernourished women.^14 15^

Despite this context-specific recommendation, the variations in formats, ingredients and nutrient composition have hindered programmatic progress. Further, there is no recommendation for supplementation of women who are breastfeeding, despite high rates of growth faltering in early life in LMICs, where maternal undernutrition is high and exclusive breastfeeding is recommended in the first 6 months of life. Recently, an expert consensus developed specifications for a fortified BEP ‘ready-to-use’ food supplement for pregnant and lactating women in LMICs.^16^ Such a product requires testing in undernourished settings for efficacy in improving birth outcomes and infant growth.

In this paper, we describe the study protocol for the Maternal Infant Nutrition Trial (MINT) at the Nepal Nutrition Intervention Project Sarlahi (NNIPS) research site in Sarlahi District, Nepal. The aim of the MINT Study is to evaluate the efficacy of daily fortified BEP supplementation during pregnancy and lactation on SGA and mean length-for-age z-score (LAZ) in the first 6 months of life. In this rural population, there is a significant burden of undernutrition among mothers and infants and high prevalence of adverse pregnancy outcomes.^17–19^ However, breastfeeding through 6 months of life is high, presenting postpartum supplementation as a plausible mechanism by which to improve infant growth. Our primary hypotheses are that (1) fortified BEP supplementation versus no supplementation during pregnancy will reduce incidence of SGA, and (2) fortified BEP supplementation versus no supplementation during lactation alone or both during pregnancy and lactation will improve infant linear growth in the first 6 months after delivery. Several secondary outcomes will also be examined.

## Methods and analysis

### Study design

**Figure 1:**
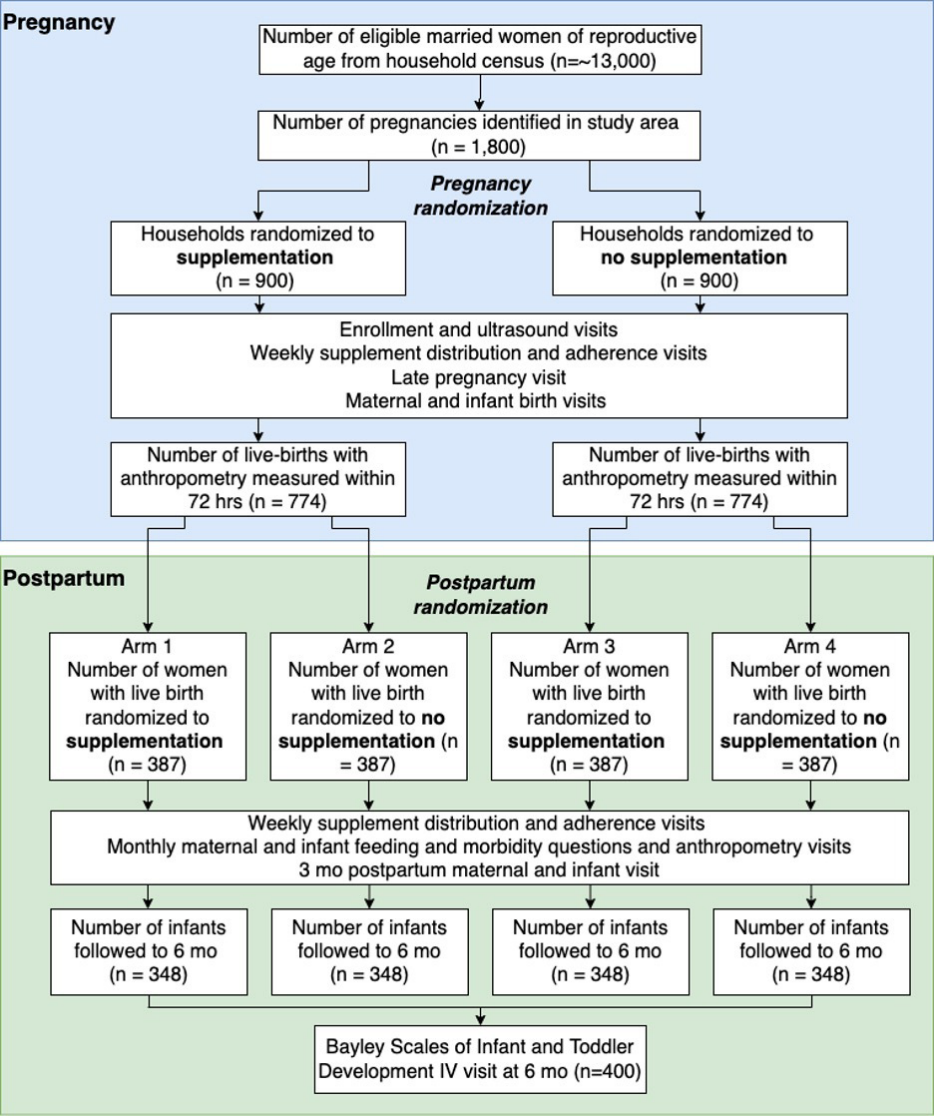
MINT Study design. MINT, Maternal Infant Nutrition Trial.

MINT is a 2×2 factorial, household randomised, unblinded, efficacy trial of fortified BEP supplementation versus no supplementation among women during pregnancy and lactation on SGA and infant growth to 6 months after delivery in rural Nepal (figure 1). This community-based trial began in November 2021, and will enrol approximately 1800 mother–fetus/infant pairs over the course of about 20 months.

We nested several substudies within the trial to explore the impact of the intervention on maternal dietary intake during pregnancy and post partum, infant neurodevelopment at 6 months after delivery, metabolites in blood during pregnancy, and the gut microbiota among mothers in pregnancy and infants in the postnatal period.

This protocol follows the Standard Protocol Items: Recommendations for Interventional Trials guidelines (online supplemental table 1).^20^

10.1136/bmjpo-2023-002229.supp1 Supplementary data

### Study setting

The MINT Study is conducted in Sarlahi District, Madhesh Province, in the Terai region (low-lying plains) of Nepal by the NNIPS research team. The NNIPS field research site, established in 1989 and covering approximately one-third of the district, currently operates as a joint undertaking between the Department of International Health, Johns Hopkins Bloomberg School of Public Health, and the non-profit organisation, Nepal Netra Jyoti Sangh, under a memorandum of agreement with the Social Welfare Council, Government of Nepal. Nepal’s Terai region has high rates of maternal undernutrition, neonatal mortality, adverse pregnancy outcomes and inadequate infant growth, and is representative of rural, low-income settings in much of South Asia.^21^ Over half (56.9%) of households in Nepal’s Madhesh Province faced food insecurity with 10% being severely food insecure, according to the Nepal Demographic and Health Survey 2016.^21^ Of children under 5 years old, 29.3% were stunted and 10.1% were wasted in Madhesh Province in 2022.^22^ Data from our most recent trial in the NNIPS study area, which had over 31 000 live births (2010–2017), reported neonatal mortality and stillbirth rates of approximately 32 per 1000 live births and 29 per 1000 births, respectively (unpublished). In that trial, the proportion of adverse pregnancy outcomes were: preterm birth 14.9%, SGA 48.5% and LBW 30.1% (unpublished). Nearly one-third of women in their first trimester of pregnancy were underweight (29.5%) (body mass index (BMI) <18.5 kg/m^2^) and the same proportion (29.5%) had a height less than 145 cm (unpublished). Over one-third of infants (35%) are breastfed within an hour of birth and 95% within 24 hours in our trial population (unpublished).

### Study population and recruitment

The study area includes 34 wards selected from six rural municipalities (Haripur, Ishwarpur, Kabilasi, Malangawa, Chakraghatta and Chandranagar) with a population of approximately 100 000 people. MINT enumerated and recruited participants through a household census and pregnancy surveillance system. Due to disruptions caused by the COVID-19 pandemic, the trial census and enrolment were divided into two periods.

In July 2019, we conducted a census to enumerate the households in the study area with potentially eligible women. The census activity identified about 27 000 total households, including roughly 13 000 households with at least one woman meeting the eligibility criteria for pregnancy surveillance. The first enrolment period spanned November 2021–June 2022, enrolling 938 women.

Between the original census and the start of pregnancy enrolment (which was delayed due to the COVID-19 pandemic), many newly married women accumulated in the study area. In December 2022 and January 2023, we conducted a new household census update activity to add these newly married women, who would be eligible for enrolment in the trial, to our census and pregnancy surveillance roster. The census update activity added about 1400 newly married women to the census roster for a total of around 14 400 women eligible for pregnancy surveillance and subsequent enrolment if they become pregnant. The second enrolment period began in January 2023 and will run until the sample size of 1800 women is met.

Pregnancy surveillance is conducted by local, female data collectors hired on the project to make household visits every 5 weeks to ask potential participants if they had a period since the last visit and offer a urine-based pregnancy test if not. All married women (15–30 years) resident in the study area at the time of the census who are identified as pregnant by our pregnancy surveillance system (<24 weeks’ gestation) and consent to participate are eligible for participation in the trial. Women who self-report an allergy to nuts, milk or soy at enrolment are excluded.

We have enrolled 1944 women as of August 2023.

### Randomisation/allocation

The study uses a household randomisation scheme as there are joint families in which more than one woman could contribute a pregnancy, which would increase risk of sharing/crossover in the context of individual randomisation. At enrolment, randomisation is stratified by maternal height (<150 cm, ≥150 cm). Using the median of three height measures of the first woman enrolled in the house, we assign each household to either supplementation or not during pregnancy within each height category. We assign any additional women who became pregnant in a household that had already been randomised to the same group. At the birth visit, we randomise within each height category and pregnancy allocation group, assigning each household to either supplementation or not during the postpartum period. Only households with at least one live birth are eligible for postpartum randomisation and follow-up. Since the likelihood of more than one woman from the same household becoming pregnant within the enrolment period is estimated to be low, at ~96.5% from prior data, this choice of randomisation approach allows us to gain the statistical strength of an individually randomised trial.

We pre-generated randomisation sequences using the R software environment for statistical programming (https://www.r-project.org/). We generated these sequences using randomly permuted blocks of size 4, 6, 8 and 12, to ensure sequential (ie, chronological) balance across the groups throughout the enrolment period.

Blinding of participants or study data collectors was not feasible due to the nature of the intervention and control.

### Intervention

At enrolment, households are randomly allocated to either supplementation or not for the duration of pregnancy. Women in the supplementation group receive at home a supply of fortified BEP supplement for daily consumption beginning at 14 gestational weeks until the pregnancy outcome. Both groups also receive (1) recommendation to enrol in ANC at a local health clinic and deliver at a certified birthing facility; (2) nutrition, hygiene, and breastfeeding and infant care counselling; and (3) a clean birthing kit. In pregnancy, women in both groups are provided iron–folic acid tablets and albendazole, if not provided via ANC at their health facility. At the birth visit, households where a woman reports at least one live birth are randomly allocated to either supplementation or not for the postpartum period until the infant is 6 months old. Counselling for exclusive breastfeeding is provided in the postpartum period but iron–folic acid tablets are not provided as per the policy in Nepal.

#### Composition of fortified BEP supplement

The fortified BEP supplement is a ready-to-eat snack in the form of lipid-based peanut paste packaged in individual sachets (72 g). The supplement was designed to meet the expert consensus specifications of a nutritious food supplement for pregnant and lactating women.^16^ Each sachet is a daily portion that provides calories (~400 kcal), protein (~14 g) and multiple micronutrients at the estimated average requirement for pregnancy. The product is made by Nutriset (France), as a specially manufactured version of their product known as ‘Plumpy’Mum’. We previously conducted two formative research studies to evaluate the acceptability of and adherence to various flavours of the BEP product in this community.^23 24^ Due to unavailability of raw materials, Nutriset produced three variations of Plumpy’Mum for use in our study, each with different combinations of ingredients selected to produce a product with a similar nutritional composition. The changes to the original ingredients were to remove the soy isolate (first change) and soy flour (second change), which resulted in minor changes in energy, protein and micronutrient concentrations, deemed inconsequential from a nutrition perspective. The specific ingredients and nutritional composition of each variation are provided in the online supplemental table 2.

### Outcomes

The study has two primary outcomes, one each for the pregnancy and postpartum/postnatal periods, respectively. The primary outcome for the pregnancy period is incidence of SGA among live born infants with birth weight measured within 72 hours of delivery. SGA is defined as weight measured within <72 hours of delivery <10th centile of the INTERGROWTH-21st reference standard by gestational age and sex.^25^ We assessed gestational age using first or second trimester ultrasound examination prior to 24 gestational weeks. The primary outcome for the postpartum/postnatal period is mean LAZ at 6 months using the international WHO international growth reference.^26^ Secondary outcomes are listed in table 1.

**Table 1:** MINT Study primary and secondary outcomes

Primary outcomes	
Small-for-gestational-age (10th centile)	Proportion of infants with birth weight <10th centile of INTERGROWTH-21st reference standard by gestational age and sex for infants measured <72 hours after delivery
Length-for-age z-score (LAZ)	Mean infant length-for-age z-score at 6 months of age using the WHO international growth reference
**Secondary outcomes**	
Newborn	
Small-for-gestational-age (3rd centile)	Proportion of infants with birth weight <3rd centile of INTERGROWTH-21st reference standard by gestational age and sex for infants measured <72 hours after delivery
Large-for-gestational-age (90th centile)	Proportion of infants with birth weight >90th centile of INTERGROWTH-21st reference standard by gestational age and sex for infants measured <72 hours after delivery
Short-for-gestational-age (10th centile)	Proportion of infants with birth length <10th centile of INTERGROWTH-21st reference standard by gestational age and sex for infants measured <72 hours after delivery
Short-for-gestational-age (3rd centile)	Proportion of infants with birth length <3rd centile of INTERGROWTH-21st reference standard by gestational age and sex for infants measured <72 hours after delivery
Birth weight	Mean infant birth weight
Birth length	Mean infant birth length
Head circumference	Mean infant head circumference
Arm circumference	Mean infant mid-upper arm circumference
Low birth weight	Proportion of infants with birth weight <2500 g for infants measured <72 hours after delivery
Gestational age	Mean infant gestational age at delivery
Preterm birth (<37 weeks)	Proportion of pregnancies that are <37 gestational weeks using the INTERGROWTH-21st standards for 1st and 2nd trimester ultrasound dating
Very, extremely preterm (<34, <32, <28 weeks)	Proportion of pregnancies that are <34, <32, <28 gestational weeks using the INTERGROWTH-21st standards for 1st and 2nd trimester ultrasound dating
Stillbirth	Proportion of pregnancies resulting in stillbirth, defined as fetal death ≥28 gestational weeks, using the INTERGROWTH-21st standards for 1st and 2nd trimester ultrasound dating
Weight-for-length z-score at 6 months of age	Mean infant weight-for-length z-score at 6 months of age using the WHO international growth reference
Weight-for-age z-score at 6 months of age	Mean infant weight-for-age z-score at 6 months of age using the WHO international growth reference
Stunting	Proportion of infants with <−2 length-for-age z-score at 6 months of age using the WHO international growth reference
Wasting	Proportion of infants with <−2 weight-for-length z-score at 6 months of age using the WHO international growth reference
Underweight	Proportion of infants with <−2 weight-for-age z-score at 6 months of age using the WHO international growth reference
Developmental scales	Bayley Scales of Infant and Toddler Development 4 assessment at 6 months
Gross motor milestones	WHO 6 gross motor milestones at 6 months
Gut microbiome	Gut microbiome diversity and stool metabolomics at 3 and 6 months
Maternal	
Gestational weight gain	Mean maternal weight gain from enrolment to late pregnancy visits
Net gestational weight gain	Mean maternal weight gain from enrolment to maternal birth assessment visit (after delivery)
Inadequate maternal gestational weight gain	Proportion of women with inadequate rate of gestational weight gain from enrolment to late pregnancy visit using the INTERGROWTH-21st reference for normal body mass index (BMI) and IOM reference for low and high BMI
Inadequate maternal net gestational weight gain	Proportion of women with inadequate rate of gestational weight gain from enrolment to maternal birth assessment visit using the INTERGROWTH-21st reference for normal BMI and IOM reference for low and high BMI
Mid-upper arm circumference	Mean change in mid-upper arm circumference from enrolment to late pregnancy visits
Anaemia	Proportion of women who are anaemic (haemoglobin <110 g/L) in late pregnancy
Weight at 6 months post partum	Mean maternal weight at 6 months post partum
Maternal dietary intake	Mean maternal dietary intake score in late pregnancy and 3 months post partum
Minimum maternal dietary intake	Proportion of women meeting the minimum dietary intake score in late pregnancy and 3 months post partum
Gut microbiome	Gut microbiome diversity and stool metabolomics during pregnancy
Metabolites	Metabolites in the blood during pregnancy

IOM, American Institute of Medicine; MINT, Maternal Infant Nutrition Trial.

### Sample size

With a sample size of 1548 woman/infant pairs visited within <72 hours of delivery, we will be able to detect a 17.5% reduction in SGA with 80% power and a 20.2% reduction with 90% power assuming a type 1 error of 5% (two-sided). Since we are assuming no interaction between the marginal effects, this sample size will allow us to detect a difference in infant LAZ at 6 months after delivery of 0.16 z-scores with 80% power and 0.18 z-scores with 90% power. The MINT Study is designed to enrol approximately 1800–2000 women over a period of 20 months (split into two 6-month enrolment periods). This will yield the required sample of 1548 woman/infant pairs visited within 72 hours of delivery, after a reduction of 14% due to fetal death and loss to follow-up in pregnancy.

### Data collection

**Figure 2:**
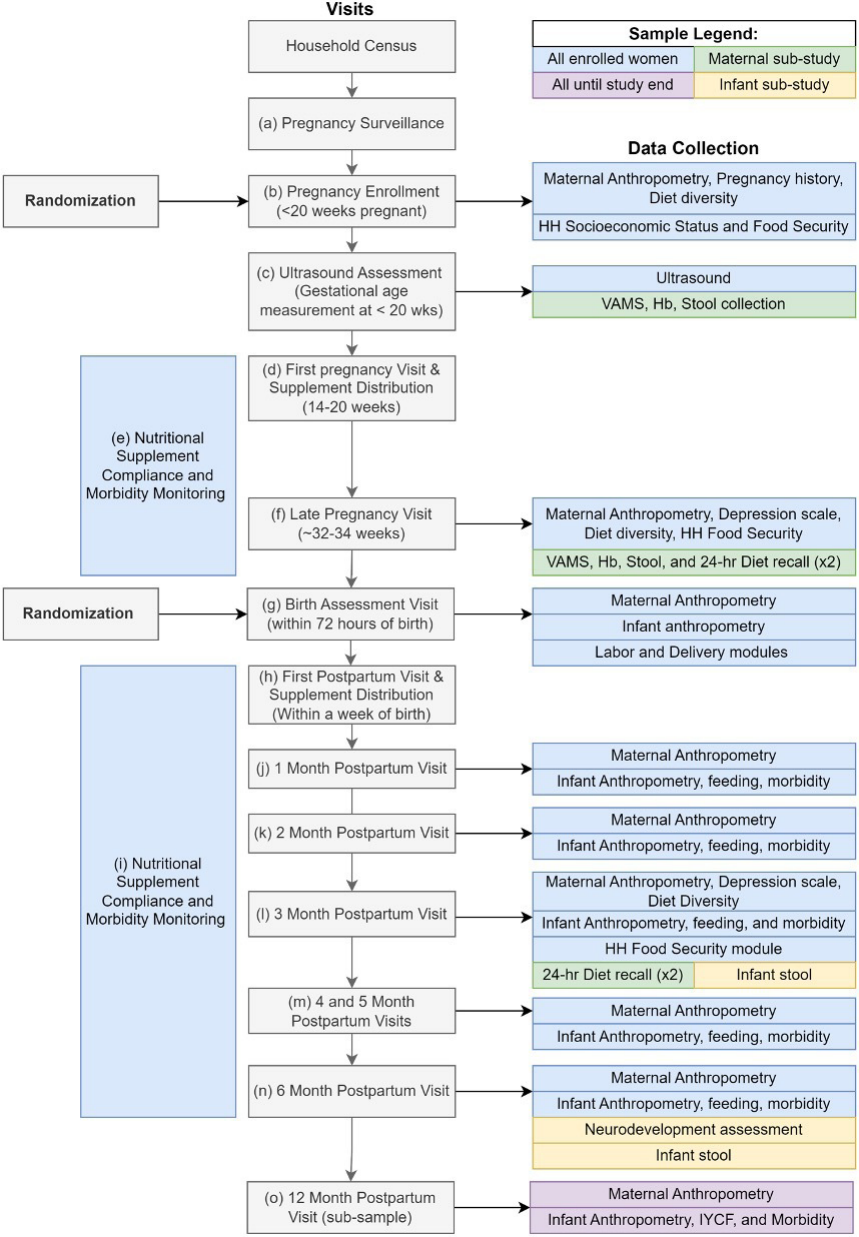
MINT Study visits and data collection. Hb, haemoglobin; HH, Household; IYCF, infant and young child feeding; MINT, Maternal Infant Nutrition Trial; VAMS, volumetric absorptive microsampling sample.

All study visits and interviews are conducted in participant households because of the wide dispersion of households across this rural community and the impracticality of bringing participants to a central location (figure 2). Data collection teams comprising field interviewers (FIs) schedule the study visits, administer the questionnaires and conduct the assessments as described below.

#### Enrolment visit

FIs conduct the enrolment visit, after notification of a newly identified pregnancy, to administer informed consent, baseline questionnaires and anthropometry. Questionnaires cover maternal demographic characteristics, socioeconomic status, pregnancy and reproductive history, dietary diversity and household food security (Household Food Insecurity Access Scale developed by the Food and Nutrition Technical Assistance).^27^ Anthropometric measurements include height (portable stadiometer), weight (MicroLife WS50 scale), mid-upper arm circumference (MUAC) (non-stretchable insertion tape), blood pressure and pulse.

#### Ultrasound visit

A special cadre of auxiliary nurse midwives (ANMs) conduct the ultrasound assessment for gestational dating by visiting the participant’s homes (online supplemental material A). ANMs are trained to use a portable machine (SonoSite NanoMaxx) and follow the INTERGROWTH-21st methodology for early and late fetal gestational age assessment, including measurement of crown-rump length and/or biparietal diameter (outer-to-outer), head circumference and femur length.^28 29^

#### Weekly morbidity and supplement compliance visits

Data collectors visit each woman weekly during pregnancy starting at 14 weeks based on last menstrual period and in the postpartum period until 6 months after delivery to provide the supplement, collect data on maternal morbidities and assess supplement compliance.

#### Late pregnancy visit

FIs conduct a late pregnancy visit between 32 and 34 gestational weeks to collect data on maternal anthropometry (same measures as enrolment), maternal morbidities, tobacco/alcohol use, ANC utilisation, delivery planning, diet diversity, household food security and depression (using a version of the Edinburgh Postnatal Depression Scale validated in Nepali).^30^

#### Birth assessment visit

FIs aim to visit each woman within 72 hours of delivery to collect information about the birth and delivery process, early breastfeeding and feeding behaviours, and newborn care practices. Infant weight (g), length (cm), head circumference (cm) and temperature (°F) are measured. Weight is measured with digital scales (Tanita BD-585), length with Shorr-type boards and MUAC with non-stretchable insertion tape. Non-digital measures are taken in triplicate and the median is used for analysis.

#### Postpartum/postnatal monthly visits

After the birth visit, FIs conduct monthly postnatal visits up to 6 months after delivery to administer questionnaires on maternal and infant morbidity, breastfeeding and infant and young child feeding practices, infant care practices, household food security and anthropometric measurements for the infant (weight, length, head circumference, MUAC) and mother (weight, MUAC). Additionally, we will visit as many woman/infant pairs as possible at 12 months after delivery until the administrative end of the study to allow us to assess infant growth at 12 months as a secondary aim in a subsample.

#### Biospecimen substudies

The MINT Study will enrol two substudies for biospecimen collection (online supplemental table 3). First, we will enrol about 120 pregnant women during the first enrolment period from the pregnancy supplementation (n=60) and not groups (n=60). At the enrolment and late pregnancy visits, these women will provide volumetric absorptive microsampling samples, for serum metabolomics analyses collected via finger-stick using Neoteryx kits, haemoglobin measurement, a maternal stool sample stored in nucleic acid buffer solution (DNA Genotek OMR-GUT), and their infants will provide a stool sample at 6 months. Second, we will enrol about 120 pregnant women during the second enrolment period from the pregnancy supplementation (n=60) and not groups (n=60), and these women will provide a haemoglobin measurement at the enrolment and late pregnancy visits, and their infants will provide a stool sample at 3 and 6 months.

#### Dietary intake

FIs will conduct duplicate 24-hour dietary recalls at the late pregnancy and 3 months postpartum visits for a subset of 240 women in the first (n=120) and second (n=120) enrolment periods. The purpose of these data is to assess nutrient intake and determine whether dietary substitution is occurring in the supplement group. Interviewers use the multiple pass method to help the woman recall all the components of her diet from the previous day. A local food list, photo atlas and electronic entry form have been adapted from previous work in Nepal to assist with portion size recall and data entry.^31 32^ Daily nutrient intake will be estimated with local recipes and national food composition tables from India and Nepal.

#### Bayley Scales of Infant and Toddler Development-4 visit

A specialised team with experience and training in developmental assessment in children will visit a subsample of 400 mother/infant pairs to evaluate infant neurodevelopment at 6 months of life.^33 34^ The visit will include the Bayley Scales of Infant and Toddler Development-4 assessment, a standardised evaluation for infants that measures functioning in five areas: cognition, fine motor, gross motor, receptive language and expressive language. At this visit, data collectors will also conduct the WHO six gross motor milestones module.^35^

### Data management

Our FIs administer study questionnaires and record anthropometric measurements via digital tablets with a cellular connection using a customised web-based data management system developed on the Microsoft ASP.NET framework. The local data collectors complete paper forms that are recorded by data entry operators on a weekly basis. On the backend, data are stored in a relational database management system (Microsoft SQL Server). Prior to study launch, the field team tests all forms, and restrictions and checks are programmed to reduce data entry errors. The senior field team member and co-investigators review important variables weekly to monitor data quality issues, such as missingness, heaping, digit preference and outliers. The senior field team member corrects data entry errors and implements retraining of data collectors on specific protocols when necessary.

### Data monitoring

#### Data and Safety Monitoring Board

An independent Data and Safety Monitoring Board (DSMB) is established for the trial to advise the investigators on issues of safety and efficacy. DSMB membership includes four individuals from Nepal, including a paediatrician, obstetrician-gynaecologist, nutritionist and statistician. The DSMB met prior to the start of the study, twice during implementation of the study and will meet at least once more.

#### Serious adverse events

Serious adverse events, as defined under Good Clinical Practice guidelines, include death, a life-threatening reaction to the supplement or other study procedures, hospitalisation for a reason other than routine delivery, significant or persistent disability or impairment, or a congenital anomaly/birth defect. All data collection staff are trained to recognise a range of serious and other adverse events. Serious adverse events are investigated and reported to the DSMB and Institutional Review Boards according to the study research and ethical protocols. Our most senior data collectors conduct verbal autopsies in the case of a maternal or infant death.

### Statistical analysis

#### Primary aim 1

We will undertake an intention-to-treat analysis to examine the effect of supplementation on the first primary outcome of SGA. The analysis will include all live births for whom a birth weight was collected by study data collectors within 72 hours of delivery for the SGA (<10th centile births using the INTERGROWTH-21st standard) outcome. We will assess the balance between the two pregnancy supplementation groups in baseline household-level and individual-level characteristics including maternal age, height, BMI, parity, previous adverse pregnancy outcome, education and socioeconomic status. If imbalanced variables are also associated with the primary outcome (p<0.10), we will use multivariable log binomial regression modelling to adjust for potential confounding. We will compare the proportion of SGA, between those who were supplemented versus not according to their allocation status, using a log binomial regression model to estimate the relative risk and 95% CI with the control group as the referent both unadjusted and adjusted for identified confounders. If the final model does not converge, we will use Poisson regression with robust SE estimation.

#### Primary aim 2

We will undertake an intention-to-treat analysis to examine the effect of supplementation on the second primary outcome of mean LAZ at 6 months. The analysis will include all children whose length was assessed at 6 months of age. We will assess the balance across the four pregnancy and lactation supplementation groups in baseline household-level and individual-level characteristics. If imbalanced variables are also associated with the secondary outcome (p<0.10), we will use multivariable linear regression modelling to adjust for potential confounding. Due to the factorial study design, we will assess the interaction between pregnancy and lactation supplementation on the outcome of mean LAZ, although our sample size is not adequate to detect small interaction effects. We will use a multivariable linear regression model with an interaction term for treatment arms. If the interaction is significant (<0.10), we will report both the main and interaction effects. If not significant, which we anticipate, we will report the marginal treatment effects for supplementation during lactation versus not and during pregnancy versus not. We will compare mean LAZ scores between supplementation groups using a multivariable linear regression model to estimate the risk difference and 95% CI with the control group as the referent both unadjusted and adjusted for identified confounders.

### Patient and public involvement

We conducted two formative studies to assess compliance with and acceptability of various formulations of BEP supplements. Our field leadership team also engaged several local stakeholders prior to the start of the study, including municipality mayors, health coordinators and health facility in charge in the study area. After completion of the study, the team will conduct similar meetings to disseminate the results.

### Ancillary care

The MINT Study provides iron–folic acid tablets and albendazole to women who report that they have not received these from the health facility during their ANC visits. Women with low haemoglobin (<110 g/L) are referred to a health facility. Women with suspected fetal abnormality detected during the ultrasound gestational age dating visit are referred for clinical examination.

### Confidentiality

Study investigators and staff treat all data as confidential, and participants are informed that their information will be protected. All data collection forms are kept in secure locations with access limited to relevant study staff. All electronic databases are stored on encrypted, password-protected servers, and data transfer uses secure encrypted transfer protocols. Access to identifying information is restricted to the principal investigator and few key co-investigators and staff. Only anonymised datasets are shared beyond the study team.

### Dissemination plan

The study team will disseminate MINT findings to the Sarlahi District Public Health Office, Sarlahi District Project Advisory Committee, Ministry of Health and Population, Nepal, Central Project Advisory Committee, and to various local organisations, including UNICEF-Nepal and UNICEF South Asian Regional Office, US Agency for International Development and other stakeholders in Nepal with interests in nutrition during pregnancy and lactation. We will share anonymised data with the Ki data repository and researchers involved in the BEP Harmonization Initiative organised and funded by the Bill & Melinda Gates Foundation for the purpose of meta-analyses using data from similar trials conducted around the world. The study team will also present results at international workshops and conferences with relevant researchers and programme or policy stakeholders. Members of the study team will publish results of primary and secondary analyses in peer-reviewed academic journals.

## Discussion

Nepal has made progress in reducing maternal, newborn and child mortality, but a significant burden of undernutrition remains among mothers and infants.^21 22^ Novel and effective interventions and practical implementation strategies are needed to prevent adverse pregnancy outcomes and infant growth faltering,^36 37^ and currently, the WHO^13^,^13^ recommends use of BEP supplements in undernourished settings, but standard formulations of such interventions and data on implementation in programmes are lacking. The MINT Study will provide evidence to help refine and implement the WHO context-specific recommendation on BEP supplementation in pregnancy, especially using the fortified, enhanced ready-to-use formulation recommended by the expert consensus on BEP supplements.^16^ As multiple micronutrient supplementation (MMS) reduces risk of adverse pregnancy outcomes, including LBW and SGA, our trial will contribute evidence as to whether the fortified BEP intervention, which includes the same 15 micronutrients, provides additional benefit beyond MMS alone.^38^ This trial will generate evidence on the potential of a maternal intervention to prevent early life growth faltering and improve maternal outcomes, such as anaemia and inadequate gestational weight gain, areas where a research gap exists. The results from the trial will inform health policy and programmes in Nepal, where there currently are no guidelines for community-level management of growth faltering in infants less than 6 months of age beyond exclusive breastfeeding. The primary strength of the trial is the 2×2 factorial randomised controlled trial design, which will enable understanding of the interconnections between maternal nutritional status in pregnancy and lactation and relationships to fetal and infant growth faltering. The trial includes frequent follow-up visits, over the continuum from early pregnancy to 6 months after delivery, which will generate data on many prenatal and postpartum factors that influence maternal and infant outcomes. The trial will describe maternal dietary intake, assess whether provision of BEP supplements leads to dietary substitution and measure adherence, yielding practical insights for implementation. Lastly, the trial structure allows for nesting various substudies, including those that assess the impact of BEP on the maternal and infant microbiome, infant neurodevelopment and maternal metabolites.

## Supplementary Material

Reviewer comments

Author's manuscript

## Data Availability

Data are available upon reasonable request. The principal investigator will share final anonymised data with the Ki data repository and researchers involved in the BEP Harmonization Initiative for the purpose of meta-analyses using data from similar trials conducted around the world.
