# Supplementary material for: Supplementation with fortified balanced energy–protein during pregnancy and lactation and its effects on birth outcomes and infant growth in southern Nepal: protocol of a 2×2 factorial randomised trial
Source: BMJ Paediatr Open. 2023 Nov 3;7(1):e002229. doi: 10.1136/bmjpo-2023-002229 (PMC10626787; doi:10.1136/bmjpo-2023-002229)
Supplement: Supplementary data [file bmjpo-2023-002229supp001.pdf]

SUPPLEMENTARY MATERIAL

Table 1: Standard protocol items: recommendations for interventional trials (SPIRIT) guidelines checklist

SPIRIT 2013 Checklist: Recommended items to address in a clinical trial protocol and related documents*

**Section/item**	**Item No**	**Description**	**Addressed on page number**
**Administrative information**			
Title	1	Descriptive title identifying the study design, population, interventions, and, if applicable, trial acronym	_____1______
Trial registration	2a	Trial identifier and registry name. If not yet registered, name of intended registry	_____1______
	2b	All items from the World Health Organization Trial Registration Data Set	_____1______
Protocol version	3	Date and version identifier	_____1______
Funding	4	Sources and types of financial, material, and other support	____10______
Roles and responsibilities	5a	Names, affiliations, and roles of protocol contributors	_____1______
	5b	Name and contact information for the trial sponsor	_____1_____

	5c	Role of study sponsor and funders, if any, in study design; collection, management, analysis, and interpretation of data; writing of the report; and the decision to submit the report for publication, including whether they will have ultimate authority over any of these activities	_____10_____
	5d	Composition, roles, and responsibilities of the coordinating centre, steering committee, endpoint adjudication committee, data management team, and other individuals or groups overseeing the trial, if applicable (see Item 21a for data monitoring committee)	_____NA_____

Introduction

Background and rationale	6a	Description of research question and justification for undertaking the trial, including summary of relevant studies (published and unpublished) examining benefits and harms for each intervention	_____2______
	6b	Explanation for choice of comparators	_____4______
Objectives	7	Specific objectives or hypotheses	_____2______
Trial design	8	Description of trial design including type of trial (eg, parallel group, crossover, factorial, single group), allocation ratio, and framework (eg, superiority, equivalence, noninferiority, exploratory)	_____2______

Methods: Participants, interventions, and outcomes

Study setting	9	Description of study settings (eg, community clinic, academic hospital) and list of countries where data will be collected. Reference to where list of study sites can be obtained	_____2______
Eligibility criteria	10	Inclusion and exclusion criteria for participants. If applicable, eligibility criteria for study centres and individuals who will perform the interventions (eg, surgeons, psychotherapists)	_____4______
Interventions	11a	Interventions for each group with sufficient detail to allow replication, including how and when they will be administered	_____4______
	11b	Criteria for discontinuing or modifying allocated interventions for a given trial participant (eg, drug dose change in response to harms, participant request, or improving/worsening disease)	_____7______
	11c	Strategies to improve adherence to intervention protocols, and any procedures for monitoring adherence (eg, drug tablet return, laboratory tests)	_____5_____
	11d	Relevant concomitant care and interventions that are permitted or prohibited during the trial	_____4______

Outcomes	12	Primary, secondary, and other outcomes, including the specific measurement variable (eg, systolic blood pressure), analysis metric (eg, change from baseline, final value, time to event), method of aggregation (eg, median, proportion), and time point for each outcome. Explanation of the clinical relevance of chosen efficacy and harm outcomes is strongly recommended	____6-7______
Participant timeline	13	Time schedule of enrolment, interventions (including any run-ins and washouts), assessments, and visits for participants. A schematic diagram is highly recommended (see Figure)	____4-7_____
Sample size	14	Estimated number of participants needed to achieve study objectives and how it was determined, including clinical and statistical assumptions supporting any sample size calculations	_____5______
Recruitment	15	Strategies for achieving adequate participant enrolment to reach target sample size	____4-5______

Methods: Assignment of interventions (for controlled trials)

Allocation:

Sequence generation	16a	Method of generating the allocation sequence (eg, computer-generated random numbers), and list of any factors for stratification. To reduce predictability of a random sequence, details of any planned restriction (eg, blocking) should be provided in a separate document that is unavailable to those who enrol participants or assign interventions	_____4______
Allocation concealment mechanism	16b	Mechanism of implementing the allocation sequence (eg, central telephone; sequentially numbered, opaque, sealed envelopes), describing any steps to conceal the sequence until interventions are assigned	_____4______
Implementation	16c	Who will generate the allocation sequence, who will enrol participants, and who will assign participants to interventions	____4-7______
Blinding (masking)	17a	Who will be blinded after assignment to interventions (eg, trial participants, care providers, outcome assessors, data analysts), and how	_____4______
	17b	If blinded, circumstances under which unblinding is permissible, and procedure for revealing a participant's allocated intervention during the trial	____NA______

Methods: Data collection, management, and analysis

Data collection methods	18a	Plans for assessment and collection of outcome, baseline, and other trial data, including any related processes to promote data quality (eg, duplicate measurements, training of assessors) and a description of study instruments (eg, questionnaires, laboratory tests) along with their reliability and validity, if known. Reference to where data collection forms can be found, if not in the protocol	____4-7_____
	18b	Plans to promote participant retention and complete follow-up, including list of any outcome data to be collected for participants who discontinue or deviate from intervention protocols	____4-7______
Data management	19	Plans for data entry, coding, security, and storage, including any related processes to promote data quality (eg, double data entry; range checks for data values). Reference to where details of data management procedures can be found, if not in the protocol	_____7_____
Statistical methods	20a	Statistical methods for analysing primary and secondary outcomes. Reference to where other details of the statistical analysis plan can be found, if not in the protocol	____7-8____
	20b	Methods for any additional analyses (eg, subgroup and adjusted analyses)	____7-8_____
	20c	Definition of analysis population relating to protocol non-adherence (eg, as randomised analysis), and any statistical methods to handle missing data (eg, multiple imputation)	____7-8_____

Methods: Monitoring

Data monitoring	21a	Composition of data monitoring committee (DMC); summary of its role and reporting structure; statement of whether it is independent from the sponsor and competing interests; and reference to where further details about its charter can be found, if not in the protocol. Alternatively, an explanation of why a DMC is not needed	_____7______
	21b	Description of any interim analyses and stopping guidelines, including who will have access to these interim results and make the final decision to terminate the trial	____NA______
Harms	22	Plans for collecting, assessing, reporting, and managing solicited and spontaneously reported adverse events and other unintended effects of trial interventions or trial conduct	_____7______
Auditing	23	Frequency and procedures for auditing trial conduct, if any, and whether the process will be independent from investigators and the sponsor	____NA______

Ethics and dissemination

Research ethics approval	24	Plans for seeking research ethics committee/institutional review board (REC/IRB) approval	_____10_____

Protocol amendments	25	Plans for communicating important protocol modifications (eg, changes to eligibility criteria, outcomes, analyses) to relevant parties (eg, investigators, REC/IRBs, trial participants, trial registries, journals, regulators)	_____9______
Consent or assent	26a	Who will obtain informed consent or assent from potential trial participants or authorised surrogates, and how (see Item 32)	_____5______
	26b	Additional consent provisions for collection and use of participant data and biological specimens in ancillary studies, if applicable	_____NA_____
Confidentiality	27	How personal information about potential and enrolled participants will be collected, shared, and maintained in order to protect confidentiality before, during, and after the trial	_____9______
Declaration of interests	28	Financial and other competing interests for principal investigators for the overall trial and each study site	_____10_____
Access to data	29	Statement of who will have access to the final trial dataset, and disclosure of contractual agreements that limit such access for investigators	_____10_____
Ancillary and post-trial care	30	Provisions, if any, for ancillary and post-trial care, and for compensation to those who suffer harm from trial participation	_____NA_____

Dissemination policy	31a	Plans for investigators and sponsor to communicate trial results to participants, healthcare professionals, the public, and other relevant groups (eg, via publication, reporting in results databases, or other data sharing arrangements), including any publication restrictions	_____9_____
	31b	Authorship eligibility guidelines and any intended use of professional writers	_____10_____
	31c	Plans, if any, for granting public access to the full protocol, participant-level dataset, and statistical code	_____10_____
**Appendices**			
Informed consent materials	32	Model consent form and other related documentation given to participants and authorised surrogates	__Section C___
Biological specimens	33	Plans for collection, laboratory evaluation, and storage of biological specimens for genetic or molecular analysis in the current trial and for future use in ancillary studies, if applicable	5, main manuscript

*It is strongly recommended that this checklist be read in conjunction with the SPIRIT 2013 Explanation & Elaboration for important clarification on the items. Amendments to the protocol should be tracked and dated. The SPIRIT checklist is copyrighted by the SPIRIT Group under the Creative Commons "Attribution-NonCommercial-NoDerivs 3.0 Unported" license.

Table 2: Balanced energy-protein supplement nutritional composition

Formula 1 ingredients: Vegetable oils (rapeseed, palm, soy in varying proportions), defatted soy flour, skimmed milk powder, peanuts, sugar, maltodextrin, soy protein isolate, vitamin and mineral complex, stabilizer (fully hydrogenated vegetable fat, mono and diglycerides). Allergens warning: peanuts, soy and dairy products. May contain traces of gluten.

Formula 2 ingredients: Vegetable oils (rapeseed, palm), skimmed milk powder, defatted soy flour, peanuts, sugar, maltodextrin, vitamin and mineral premix, fully hydrogenated vegetable oil (palm). Allergens warning: peanuts, soy and dairy products. May contain traces of gluten.

Formula 3 ingredients: Peanuts, vegetable oil (rapeseed), skimmed milk powder, sugar, whey protein concentrate, maltodextrin, premix of vitamins and minerals, fully hydrogenated vegetable oil (palm), flavor. Allergens warning: peanuts and dairy products. May contain traces of soy.

		**Formula 1**		**Formula 2**		**Formula 3**	
		**RUSF P&L 72g**		**Reformulation 1 RUSF P&L 72g**		**Reformulation RUSF 2 P&L 72**	
**Nutrients**	**Unit**	Mean / 100g	Mean / dose 72g	Mean / 100g	Mean / dose 72g	Mean / 100g	Mean / dose 72g
**Energy**	**kcal**	546	393	544	391	557	401
**Protein**	**g**	20.2	14.5	20.4	14.7	20.2	14.5
**Lipids**	**g**	36	26	36	26	37.3	26.9
**Linoleic Acid**	**g**	5.4	3.9	5.4	3.9	6.2	4.5
**alpha-Linolenic Acid**	**g**	1.8	1.3	1.9	1.4	1.8	1.3
**Calcium**	**mg**	694	500	776	559	695	500
**Zinc**	**mg**	21	15	21	15	21	15
**Copper**	**mg**	1.8	1.3	1.8	1.3	1.8	1.3

**Iron**	**mg**	31	22	30	22	31	22
**Iodine**	**ug**	347	250	360	260	348	250
**Selenium**	**ug**	90	65	91	65	94	68
**Manganese**	**mg**	2.9	2.1	2.9	2.1	2.9	21
**Magnesium**	**mg**	102	73	108	78	99	72
**Free phosphorus**	**mg**	580	418	638	459	664	478
**Potassium**	**mg**	781	562	892	642	800	576
**Vitamin A**	**ug RE**	1069	770	1069	770	1,069	770
**Vitamin B1**	**mg**	1.9	1.4	1.9	1.4	1.9	1.4
**Vitamin B2**	**mg**	1.9	1.4	1.9	1.4	1.9	1.4
**Niacin**	**mg**	21	15	21	15	21	15
**Vitamin B5**	**mg**	9.7	7	9.7	7	9.7	7.0
**Vitamin B6**	**mg**	2.6	1.9	2.6	1.9	2.6	1.9
**Folic Acid**	**ug**	556	400	556	400	556	400
**Vitamin B12**	**ug**	3.6	2.6	3.6	2.6	3.6	2.6
**Vitamin C**	**mg**	139	100	139	100	139	100
**Vitamin D (Cholecacifero l)**	**ug**	21	15	21	15	21	15

**Vitamin E (alpha tocopherol)**	**mg**	25	18	25	18	25	16
**Vitamin K**	**ug**	100	72	100	72	100	72

Table 3: Summary of sub-study enrollments

This study has three sub-cohorts: two biospecimen cohorts and a neurodevelopment sub-study cohort.

	**1st biospecimen sub- study (n=100 women/infant pairs)**	**2nd biospecimen sub- study (n=120 study women/infant pairs)**	**Neurodevelopment sub-study (n=400 infants) women/infant pairs)**
**Hemoglobin (early and late pregnancy)**	100	120	N/A
**VAMS (early and late pregnancy)**	100	0	N/A
**Maternal stool (early and late pregnancy)**	100	0	N/A
**Infant stool**	100 (6 months)	120 (3, 6 months)	N/A
**Dietary 24 recall visit (2x visits in pregnancy and 3-months postpartum)**	100	120	N/A
**Neurodevelopment assessment visit at 6 months postnatal**	N/A	N/A	400

Section A: Ultrasound gestational age dating protocol

Purpose: The purpose of the ultrasound exam is to collect data on fetal measurements using transabdominal ultrasound to estimate gestational age. Gestational age is required for calculation of our primary outcome, incidence of small-for-gestational-age (<10th centile) among live born infants whose birthweight is measured within 72 hours of delivery. Gestational age is also required construction of secondary outcomes, such as preterm birth.

Staff: The ultrasound gestational age exam is conducted by a specialized team of Auxiliary Nurse Midwives (ANMs) and assistants in this study. Previously, ANMs in our field team have undergone extensive training for in-home ultrasound examination to identify non-cephalic presentation, multiple births, placenta previa, and transabdominal ultrasound exam for gestational age dating. These trainings have been conducted by staff at the Department of Radiology at Tribhuvan University in Kathmandu. ANMs in this study receive similar training and oversight.

Measurements: The MINT study schedules participants for the ultrasound exam as soon as possible after identification of a pregnancy through our pregnancy surveillance system and the enrollment visit. The study aims to complete the ultrasound exam as earlier as feasible after pregnancy identification, ideally in the first trimester, for all participants to ensure the most accurate results. As part of the ultrasound exam, ANMs measure and record several measurements, depending upon the gestation at time of the exam, including crown rump length (CRL), biparietal diameter (BPD), head circumference (HC), and femur length (FL). The measurements and calculations follow the INTERGROWTH 21st methodology for early and late fetal gestational age assessment (Papageorghiou 2014, Papageorghiou 2016). In brief, the ANMs first attempt to measure CRL. If CRL is <15 mm, participants are rescheduled for another ultrasound exam in the following weeks. If CRL is ≥15 mm and <85 mm, the ANM will measure and record three CRL measurements. If CRL is ≥85 to ≤95 mm, in addition to CRL, the ANM also measure and record three values for BPD, HC, and FL. If CRL is >95 mm or CRL cannot be measured because the fetus is too large to fit in the field of view, the ANM will only measure and record three values for BPD, HC, and FL. Biparental diameter is measured "outer-to-outer." The median measure for each anatomical feature is used for calculation of gestational age using the INTERGROWTH 21st formulas (Papageorghiou 2014, Papageorghiou 2016). Suspected abnormalities are recorded for review and referral by the study radiologist.

Quality control: ANMs record measurements and save digital images from the exam for review by senior field staff and a radiologist who are not present at the home visit. The radiologist reviews a 10-15% sample of images collected by the ANMs to validate that correct imaging and measurement techniques are used for each anatomical feature following the INTERGROWTH-21st Study Protocol (International Fetal and Newborn Growth Consortium 2009).

Equipment: We use the Sonosite NanoMaxx ultrasound machine for our study. The machine is FDA approved, and the specifications are as follows:

- Product: Nanomaxx ultrasound machine
- Weight: 6 lbs / 2.7 kg (with battery)
- Dimensions: 14.1" L x 8.2" H x 2.3" W / 35.8 cm L x 20.8 cm H x 5.8 cm W
- Display: 8.4" / 21.3 cm diagonal display LCD touch screen
- Architecture: All-digital broadband
- Dynamic Range: Up to 165 dB
- Gray Scale: 200 dpi (voltage axis) x 500 dpi (time axis)
- HIPAA compliance: Comprehensive tool set
- Manufacturer: Sonosite

References:

Papageorghiou AT, Kennedy SH, Salomon LJ, Ohuma EO, Cheikh Ismail L, Barros FC, Lambert A, Carvalho M, Jaffer YA, Bertino E, Gravett MG, Altman DG, Purwar M, Noble JA, Pang R, Victora CG, Bhutta ZA, Villar J; International Fetal and Newborn Growth Consortium for the 21st Century (INTERGROWTH-21st). International standards for early fetal size and pregnancy dating based on ultrasound measurement of crown-rump length in the first trimester of pregnancy. Ultrasound Obstet Gynecol. 2014 Dec;44(6):641-8.

Papageorghiou AT, Kemp B, Stones W, Ohuma EO, Kennedy SH, Purwar M, Salomon LJ, Altman DG, Noble JA, Bertino E, Gravett MG, Pang R, Cheikh Ismail L, Barros FC, Lambert A, Jaffer YA, Victora CG, Bhutta ZA, Villar J; International Fetal and Newborn Growth Consortium for the 21st Century (INTERGROWTH-21st). Ultrasound-based gestational-age estimation in late pregnancy. Ultrasound Obstet Gynecol. 2016 Dec;48(6):719-726.

International Fetal and Newborn Growth Consortium. The International Fetal and Newborn Growth Standards for the 21st Century (INTERGROWTH-21st) Study Protocol, 2009, www.intergrowth21.org.uk

Section B: Informed consent document

Nepal Pregnancy and Lactation Nutritional Supplementation Project

Consent for Participation in the Phase 3 Study

GREETING: Namaste! I am a Nepal Nutrition Intervention Project-Sarlahi (NNIPS) staff person. As you may know, NNIPS has been doing research projects concerning the health of mothers and children in your community for the past 30 years. Today, I would like to talk with you about a new NNIPS project. This project is being jointly run by NNIPS, Nepal Netra Jyoti Sangh, George Washington University, and Johns Hopkins University in the USA. I would like to tell you more this new project, ask for your consent to take part in it, and answer any questions you may have about the project.

PURPOSE

We are doing this new research study to learn more about nutrition for pregnant and breastfeeding women in our community. Specifically, we would like to learn if adding a special diet supplement (snack created to meet the needs of pregnant women in this area) to the diet of pregnant and breastfeeding women will improve the health and growth of their newborns and young babies. We would like to compare women who do and don't use this special diet supplement along with their normal diet. We are asking all pregnant women aged 15-30 in this community if they would like to participate in this study, until about 1800 women have agreed to be in the study. This study may help inform the government about ways to improve the health of babies and young infants in Nepal.

PROCEDURES

If you choose to take part in this project, I will ask you some questions today about your household and your family, and also ask about your previous pregnancies. I will also ask you about your current pregnancy, your health and diet, and measure your height, weight, and your blood pressure. This visit will take approximately 30-45 minutes. I will also schedule you to have an ultrasound examination by one of our Auxiliary Nurse-Midwives to check on how far along in pregnancy you are and to take a finger stick blood sample to measure your hemoglobin level. Once you are more than 14 weeks after your last period you will be asked to do one of 4 activities:

- Keep diet and level of activity as you normally would during your pregnancy and while you are breastfeeding.
- Take a special supplement to your diet while you are pregnant but then resume your regular diet after having your baby while you are breastfeeding.
- Keep your regular diet while you are pregnant, but take a special supplement to your diet during the first 6 months after having your baby, while you are breastfeeding.
- Take a special supplement to your diet while you are pregnant and after you have your baby for the first 6 months while you are breastfeeding.

You and the other pregnant women in your neighborhood will be assigned to one of these activities by chance. It is very important that you take the nutritional supplement every day if you are assigned to a supplement group and that you don't change your regular diet.

I or another NNIPS worker will visit you to provide you with these special supplements and ask about your use of the supplements on a weekly basis. These visits will only take about 10 minutes.

Once late in your pregnancy, I or another NNIPS worker will visit to ask about your health during your pregnancy and to measure your weight and blood pressure.

We will visit you on the day your baby is born and ask some questions about the delivery and the health of you and your baby. We will also measure your baby's size and temperature. This visit will take about 30 minutes. We will continue to visit weekly until your baby is 6 months old. Once a month for 6 months, we will measure your baby's weight and length and your weight and ask some questions about the baby's and your health. These visits will take about 25 minutes. For some women and their infants, we will visit again when your baby is 12 months old to measure your baby's weight and length and your weight.

Altogether, you will be in this study for about 14 months.

RISKS/DISCOMFORTS

If you are allergic to milk, nuts (including peanuts) or soy you should not participate in this study. There are no significant risks to you or your baby if you take part in this study. There are minor risks to your privacy, but we will work hard to keep your information and participation confidential. You may feel uncomfortable with some of the questions we ask. If you feel at all uncomfortable with any of the questions we ask, you can just skip those questions. In the short interviews today and during the follow up visits, if there are any questions that are sensitive, you do not have to answer. You may experience slight pain in your finger when we take the blood sample.

As you know, proper nutrition in pregnancy is important for the health of babies in our community. While there are many potential benefits of better nutrition in pregnancy, it is possible that the nutritional supplement that we provide may not taste good to you.

BENEFITS

There are some benefits to participating in this study. We will provide you with a clean delivery kit, iron tablets, medicine for worms, and a baby blanket. We will also provide tetanus vaccine to you if you need it and have not received it at your routine antenatal care visit. We ask that you take the medicine for worms only after you are in your second trimester of pregnancy. The iron tablets, deworming medicine, and tetanus vaccine will improve the health of you during your pregnancy and will also improve the health of your growing baby.

All pregnant women and babies will be checked by our NNIPS workers, and while they cannot provide medical care, they can identify when your baby might be sick and can give you advice on when and where to seek care. The government may provide this care for free or you may have to pay some for it. We strongly encourage you to go to the local health center for antenatal care as well and to deliver your baby at an official birthing center.

We will tell you your hemoglobin level the day we take the finger stick blood sample. This will help determine the strength of your blood. If your hemoglobin level is too low, we will encourage you to take your iron tablets and, if needed, refer you to your local health center.

The information we learn in this study may be helpful for current and future babies in your community.

CONFIDENTIALITY

All information you provide will remain secure with NNIPS staff and will not be shared with anyone who is not working in this project or those organizations who monitor research studies to be sure they are being done properly. We will share your information with the funding agency and other partners for this project but none of that information will identify you personally. We will keep all the forms and data in locked cabinets at our NNIPS office and in protected computer files. The information you provide will only be used for this research study.

With your permission, we want to keep your information in our files in case there will be another NNIPS study in the future.

VOLUNTARY PARTICIPATION

You have a choice to be in this project and you may say yes or no. If you do not wish to be in this project, your other option is to follow the diet you want to, and have routine care given to women in this area.

If you do agree to participate, you can also stop or withdraw from the study at any time. You will also be able to withdraw your permission for us to share your information with the funding agency and other partners for this project. This will not affect your access to health care or NNIPS activities now or in the future. All things provided to you in this study are free, but you will not be otherwise compensated for your participation.

PERSON TO CONTACT

If you have any questions or problems about the study, I can answer them now, or you can contact the Project Director, Dr Subarna Khatry at NNIPS office in Hariaun, Sarlahi (phone no. 046-530135). You can also contact the Nepal Health Research Council in Kathmandu if you have any concerns (phone no. 1-4227460).

You can also contact these people if you believe you have been injured by being in this study.

Do you give permission to participate in this study? Check: Yes _____ No _____

Do you give permission to share your information with the funding agency and other partners?

Check: Yes _______   No _________

Do you give permission to be contacted for future studies?   Check: Yes ___ No ___

PERMISSION TO PROCEED

Woman Consenting:

Name Signature Date

NNIPS Staff Witnessing Consent:

Name Signature Date

Husband's or Guardian's Consent for Women Younger than 18 yrs.:

Name Signature Date
