# Supplementary material for: Supplementation with fortified balanced energy–protein during pregnancy and lactation and its effects on birth outcomes and infant growth in southern Nepal: protocol of a 2×2 factorial randomised trial
Source: BMJ Paediatr Open. 2023 Nov 3;7(1):e002229. doi: 10.1136/bmjpo-2023-002229 (PMC10626787; doi:10.1136/bmjpo-2023-002229)
Supplement: Author's manuscript [file bmjpo-2023-002229.draft_revisions.pdf]

BMJ Paediatrics Open

BMJ Paediatrics Open is committed to open peer review. As part of this commitment we make the peer review history of every article we publish publicly available.

When an article is published we post the peer reviewers' comments and the authors' responses online. We also post the versions of the paper that were used during peer review. These are the versions that the peer review comments apply to.

The versions of the paper that follow are the versions that were submitted during the peer review process. They are not the versions of record or the final published versions. They should not be cited or distributed as the published version of this manuscript.

BMJ Paediatrics Open is an open access journal and the full, final, typeset and author-corrected version of record of the manuscript is available on our site with no access controls, subscription charges or pay-per-view fees (http://bmjpaedsopen.bmj.com).

If you have any questions on BMJ Paediatrics Open's open peer review process please email info.bmjpo@bmj.com

BMJ Paediatrics Open

Fortified balanced energy-protein supplementation during pregnancy and lactation: protocol of a 2x2 factorial randomized trial to improve birth outcomes and infant growth in southern Nepal

Journal:	*BMJ Paediatrics Open*
Manuscript ID	bmjpo-2023-002229
Article Type:	Protocol
Date Submitted by the Author:	16-Aug-2023
Complete List of Authors:	Erchick, Daniel; Johns Hopkins Bloomberg School of Public Health, Department of International Health<br>Lama, Tsering P.; Johns Hopkins Bloomberg School of Public Health, Department of International Health; Nepal Nutrition Intervention Project Sarlahi (NNIPS)<br>Khatry, Subarna K.; Nepal Nutrition Intervention Project Sarlahi (NNIPS)<br>Katz, Joanne; Johns Hopkins Bloomberg School of Public Health, Department of International Health<br>Mullany, Luke C.; Johns Hopkins Bloomberg School of Public Health, Department of International Health<br>Zavala, Eleonor; Johns Hopkins Bloomberg School of Public Health, Department of International Health<br>LeClerq, Steven C.; Johns Hopkins Bloomberg School of Public Health, Department of International Health; Nepal Nutrition Intervention Project Sarlahi (NNIPS)<br>Christian, Parul; Johns Hopkins Bloomberg School of Public Health, Department of International Health<br>Tielsch, James M.; George Washington University, Department of Global Health
Keywords:	Epidemiology, Infant

SCHOLARONE™
Manuscripts

I, the Submitting Author has the right to grant and does grant on behalf of all authors of the Work (as defined in the below author licence), an exclusive licence and/or a non-exclusive licence for contributions from authors who are: i) UK Crown employees; ii) where BMJ has agreed a CC-BY licence shall apply, and/or iii) in accordance with the terms applicable for US Federal Government officers or employees acting as part of their official duties; on a worldwide, perpetual, irrevocable, royalty-free basis to BMJ Publishing Group Ltd ("BMJ") its licensees and where the relevant Journal is co-owned by BMJ to the co-owners of the Journal, to publish the Work in this journal and any other BMJ products and to exploit all rights, as set out in our licence.

The Submitting Author accepts and understands that any supply made under these terms is made by BMJ to the Submitting Author unless you are acting as an employee on behalf of your employer or a postgraduate student of an affiliated institution which is paying any applicable article publishing charge ("APC") for Open Access articles. Where the Submitting Author wishes to make the Work available on an Open Access basis (and intends to pay the relevant APC), the terms of reuse of such Open Access shall be governed by a Creative Commons licence – details of these licences and which Creative Commons licence will apply to this Work are set out in our licence referred to above.

Other than as permitted in any relevant BMJ Author's Self Archiving Policies, I confirm this Work has not been accepted for publication elsewhere, is not being considered for publication elsewhere and does not duplicate material already published. I confirm all authors consent to publication of this Work and authorise the granting of this licence.

Confidential: For Review Only

Fortified balanced energy-protein supplementation during pregnancy and lactation: protocol of a 2x2 factorial randomized trial to improve birth outcomes and infant growth in southern Nepal

Daniel J. Erchick[1], Tsering P. Lama[1,2], Subarna K. Khatry[2], Joanne Katz[1], Luke C. Mullany[1], Eleonor Zavala[1], Steven C. LeClerq[1,2], Parul Christian[1], James M. Tielsch[3]

[1] Department of International Health, Johns Hopkins Bloomberg School of Public Health, Baltimore, MD, USA

[2] Nepal Nutrition Intervention Project Sarlahi (NNIPS), Kathmandu, Nepal

[3] Department of Global Health, Milken Institute School of Public Health, George Washington University, Washington, District of Columbia, USA

Corresponding author: Daniel Erchick, Department of International Health, Johns Hopkins Bloomberg School of Public Health, Baltimore, MD, USA. derchick@jhu.edu

Confidential: For Review Only

Abstract

Introduction: Many women in low and middle-income countries (LMICs) enter pregnancy with low nutritional reserves with increased risk for fetal growth restriction (FGR) and poor birth outcomes, including small-for-gestational-age (SGA) and preterm birth. Balanced energy-protein (BEP) supplements have shown reductions in risk of stillbirth and SGA, yet variations in intervention format and composition and limited evidence on the impact of BEP during lactation on growth outcomes, warrant further study. This paper describes the protocol of the Maternal Infant Nutrition Trial (MINT) study, which aims to evaluate the impact of a fortified BEP supplement during pregnancy and lactation on birth outcomes and infant growth in rural Nepal.

Methods and analysis: MINT is a 2x2 factorial, household randomized, unmasked, efficacy trial conducted in a sub-area of Sarlahi District, Nepal. The study area covers six rural municipalities with about 27,000 households and a population of approximately 100,000. Married women (15-30 years) who become pregnant are eligible for participation in the trial and are randomly assigned at enrollment to supplementation with fortified BEP or not and at birth to fortified BEP supplementation or not until 6 months postpartum. The primary pregnancy outcome is incidence of SGA, using the INTERGROWTH-21st standard, among live born infants with birth weight measured within 72 hours of delivery. The primary infant growth outcome is mean length-for-age z-score (LAZ) at 6 months using the WHO international growth reference.

Ethics and dissemination: The study was approved by Institutional Review Board (IRB) at Johns Hopkins Bloomberg School of Public Health, Baltimore, USA, (IRB00009714), the Committee on Human Research IRB at The George Washington University, Washington, DC, USA (081739), and the Ethical Review Board of the Nepal Health Research Council, Kathmandu, Nepal (174/2018).

Trial registration number: NCT03668977

Key words: Balanced energy-protein, maternal nutrition, small-for-gestational-age, pregnancy outcomes, infant growth, randomized trial, Nepal

Protocol version: Version 6, July 20, 2023

Confidential: For Review Only

KEY MESSAGES

What is already known on this topic: The World Health Organization recommendations on antenatal care include balanced energy-protein (BEP) supplementation in undernourished populations to reduce the risk of stillbirth and SGA birth.

What this study hopes to add: This study will demonstrate whether fortified BEP reduces risk of adverse pregnancy outcomes beyond multiple micronutrient supplementation. MINT will also generate evidence whether maternal intervention prevents early life growth faltering and improves maternal outcomes, such as anemia and inadequate gestational weight gain.

How this study might affect research, practice or policy: The MINT trial will help refine and implement the WHO context-specific recommendation on BEP supplementation in pregnancy and provide evidence for potential intervention in pregnancy and postpartum on infant growth outcomes.

Confidential: For Review Only

INTRODUCTION

Many women in low and middle-income countries (LMICs) enter pregnancy with low nutritional reserves, unable to meet the increased requirements for protein, energy, and micronutrients during pregnancy and lactation. Undernourished women are at increased risk of fetal growth restriction (FGR) and adverse birth outcomes, including preterm birth (<37 gestational weeks) and small-for-gestational age (SGA) (birth weight <10th percentile of a reference standard by gestational age and sex), two underlying biologic causes of low birth weight (LBW) (<2.5 kg).[1] SGA infants are more likely to experience higher mortality, linear growth faltering and poor health outcomes, poor cognitive performance, and diminished educational and employment attainment.[2-4] In 2020, 23.4 million infants (17.4% of all births) were born SGA, with the highest prevalence in South Asia (40.9%).[5, 6] Estimates from 2012 indicate that 22% neonatal deaths in LMICs, and 26% in South Asia, were attributable to SGA.[7, 8]

Infant size status at birth and growth in the first 6-months of life, especially among preterm and SGA infants, are critical determinants of childhood stunting and wasting associated with higher risk for mortality, morbidity, and poor developmental outcomes.[9] Many maternal risk factors associated with pregnancy outcomes and consequent preterm or SGA are likely contributors to linear growth failure.[10] Postnatal factors, such as breastfeeding and feeding behaviors, infections, and environmental factors, are also important drivers of infant growth.[11, 12]

Nutritional interventions targeted to women during pregnancy and lactation provide an opportunity to break the intergenerational cycle of growth failure. The World Health Organization (WHO) recommendations on antenatal care (ANC) include balanced energy-protein (BEP) supplementation in undernourished populations to reduce the risk of stillbirth and SGA birth.[13] This is based on a systematic review of BEP supplementation during pregnancy that has shown decreased risk of stillbirth (relative risk (RR): 0.60, 95% confidence interval (CI): 0.39 to 0.94) and SGA (RR: 0.79, 95% CI: 0.69 to 0.90),.[14] BEP also increased mean infant birth weight by 41g overall and 100g among undernourished women.[14, 15]

Despite this context-specific recommendation, the variation in formats, ingredients, and nutrient composition have hindered programmatic progress. Further, there is no recommendation for supplementation of women who are breastfeeding, despite high rates of growth faltering in early life in LMICs, where maternal undernutrition is high and exclusive breastfeeding is recommended in the first 6 months of life. Recently, an expert consensus developed specifications for a fortified BEP “ready-to-use” food supplement for pregnant and lactating women in LMICs.[16] Such a product requires testing in undernourished settings for efficacy in improving birth outcomes and infant growth.

In this paper, we describe the study protocol for the Maternal Infant Nutrition Trial (MINT) at the Nepal Nutrition Intervention Project Sarlahi (NNIPS) research site in Sarlahi District, Nepal. The aim of the MINT study is to evaluate the efficacy of daily fortified BEP supplementation during pregnancy and lactation on SGA and mean length-for-age z-score (LAZ) in the first six months of life. In this rural population, there is a significant burden of undernutrition among mothers and infants and high prevalence of adverse pregnancy outcomes.[17-19] However, breastfeeding

Confidential: For Review Only

through 6 months of life is high, presenting postpartum supplementation as a plausible mechanism by which to improve infant growth. Our primary hypotheses are that 1) fortified BEP supplementation vs. no supplementation during pregnancy will reduce incidence of SGA, and 2) fortified BEP supplementation vs. no supplementation during lactation alone or both during pregnancy and lactation will improve infant linear growth in the first six months after delivery. Several secondary outcomes will also be examined.

Confidential: For Review Only

METHODS AND ANALYSIS

Study design

MINT is a 2x2 factorial, household randomized, unmasked, efficacy trial of fortified BEP supplementation vs. no supplementation among women during pregnancy and lactation on SGA and infant growth to 6 months after delivery in rural Nepal (Figure 1). This community-based trial began in November 2021, and will enroll approximately 1,800 mother-fetus/infant pairs over the course of about 20 months.

We nested several sub-studies within the trial to explore the impact of the intervention on maternal dietary intake during pregnancy and postpartum, infant neurodevelopment at 6 months after delivery, metabolites in blood during pregnancy, and the gut microbiota among mothers in pregnancy and infants in the postnatal period.

This protocol follows the Standard Protocol Items: Recommendations for Interventional Trials (SPIRIT) guidelines (Supplementary Material, Table 1).[20]

Study setting

The MINT study is conducted in Sarlahi District, Madhesh Province, in the Terai region (low lying plains) of Nepal by the NNIPS research team. The NNIPS field research site, established in 1989 and covering approximately one-third of the district, currently operates as a joint undertaking between the Department of International Health, Johns Hopkins Bloomberg School of Public Health (JHSPH), and the nonprofit organization, Nepal Netra Jyoti Sangh, under a memorandum of agreement with the Social Welfare Council, Government of Nepal. Nepal's Terai region has high rates of maternal undernutrition, neonatal mortality, adverse pregnancy outcomes, and inadequate infant growth, and is representative of rural, low-income settings in much of South Asia.[21] Over half (56.9%) of households in Nepal's Madhesh Province faced food insecurity with 10% being severely food insecure, according to the Nepal Demographic and Health Survey 2016.[21] Of children under five years old, 29.3% were stunted and 10.1% were wasted in Madhesh Province in 2022.[22] Data from our most recent trial in the NNIPS study area, which had over 31,000 live births (2010-2017), reported neonatal mortality and stillbirth rates of approximately 32 per 1,000 live births and 29 per 1,000 births respectively (unpublished). In that trial, the proportion of adverse pregnancy outcomes were: preterm birth 14.9%, SGA 48.5%, and LBW 30.1% (unpublished). Nearly a third of women in their first trimester of pregnancy were underweight (29.5%) (body mass index (BMI) <18.5 kg/m^2) and the same proportion (29.5%) had a height less than 145 cm (unpublished). Over a third of infants (35%) are breastfed within an hour of birth and 95% within 24 hours in our trial population (unpublished).

Study population and recruitment

The study area includes 34 wards selected from six rural municipalities (Haripur, Ishwarpur, Kabilasi, Malangawa, Chakraghatta and Chandranagar) with a population of approximately 100,000 people. MINT enumerated and recruited participants through a household census and

Confidential: For Review Only

pregnancy surveillance system. Due to disruptions caused by the COVID-19 pandemic, the trial census and enrollment was divided into two periods.

In July 2019, we conducted a census to enumerate the households in the study area with potentially eligible women. The census activity identified about 27,000 total households, including roughly 13,000 households with at least one woman meeting the eligibility criteria for pregnancy surveillance. The first enrollment period spanned November 2021 to June 2022, enrolling 938 women.

Between the original census and the start of pregnancy enrollment (which was delayed due to the COVID-19 pandemic), many newly married women accumulated in the study area. In December 2022 and January 2023, we conducted a new household census update activity to add these newly married women, who would be eligible for enrollment in the trial, to our census and pregnancy surveillance roster. The census update activity added about 1,400 newly married women to the census roster for a total of around 14,400 women eligible for pregnancy surveillance and subsequent enrollment if they become pregnant. The second enrollment period began in January 2023 and will run until the sample size of 1,800 women is met.

Pregnancy surveillance is conducted by local, female data collectors hired on the project to make household visits every 5 weeks to ask potential participants if they had a period since the last visit and offer a urine-based pregnancy test if not. All married women (15-30 years) resident in the study area at the time of the census who are identified as pregnant by our pregnancy surveillance system (<24 weeks gestation) and consent to participate are eligible for participation in the trial. Women who self-report an allergy to nuts, milk, or soy at enrollment are excluded.

Randomization/allocation

The study uses a household-randomization scheme as there are joint families in which more than one woman could contribute a pregnancy, which would increase risk of sharing/crossover in the context of individual randomization. At enrollment, randomization is stratified by maternal height (<150 cm, ≥150 cm). Using the median of three height measures of the first woman enrolled in the house, we assign each household to either supplementation or not during pregnancy within each height category. We assign any additional women who became pregnant in a household that had already been randomized to the same group. At the birth visit, we randomize within each height category and pregnancy allocation group, assigning each household to either supplementation or not during the postpartum period. Only households with at least one live birth are eligible for postpartum randomization and follow-up. Since the likelihood of more than one woman from the same household becoming pregnant within the enrollment period is estimated to be low, at ~96.5% from prior data , this choice of randomization approach allows us to gain the statistical strength of an individually randomized trial.

We pre-generated randomization sequences using the R software environment for statistical programming (www.r-project.org). We generated these sequences using randomly permuted

Confidential: For Review Only

blocks of size 4, 6, 8, and 12, to ensure sequential (i.e., chronological) balance across the groups throughout the enrollment period.

Masking of participants or study data collectors was not feasible due to the nature of the intervention and control.

Intervention

At enrollment, households are randomly allocated to either supplementation or not for the duration of pregnancy. Women in the supplementation group receive at home a supply of fortified BEP supplement for daily consumption beginning at 14 gestational weeks until the pregnancy outcome. Both groups also receive i) recommendation to enroll in antenatal care (ANC) at a local health clinic and deliver at a certified birthing facility, ii) nutrition, hygiene, and breast feeding and infant care counseling, and iii) a clean birthing kit. In pregnancy, women in both groups are provided iron-folic acid tablets and albendazole, if not provided via antenatal care at their health facility. At the birth visit, households where a woman reports at least one live birth are randomly allocated to either supplementation or not for the postpartum period until the infant is 6 months old. Counseling for exclusive breast feeding is provided in the postpartum period but iron-folic acid tablets are not provided as per the policy in Nepal.

Composition of fortified BEP supplement

The fortified BEP supplement is a ready-to-eat snack in the form of lipid-based peanut paste packaged in individual sachets (72g). The supplement was designed to meet the expert consensus specifications of a nutritious food supplement for pregnant and lactating women.[16] Each sachet is a daily portion that provides calories (~400 kcal), protein (~14 g), and multiple micronutrients at the estimated average requirement for pregnancy. The product is made by Nutriset, France, as a specially-manufactured version of their product known as “Plumpy’Mum”. We previously conducted two formative research studies to evaluate the acceptability of and adherence to various flavors of the BEP product in this community.[23, 24] Due to unavailability of raw materials, Nutriset produced three variations of Plumpy’Mum for use in our study, each with different combinations of ingredients selected to produce a product with a similar nutritional composition. The changes to the original ingredients were to remove the soy isolate (first change) and soy flour (second change), which resulted in minor changes in energy, protein, and micronutrient concentrations, deemed inconsequential from a nutrition perspective. The specific ingredients and nutritional composition of each variation are provided in the Supplementary Material, Table 2.

Outcomes

The study has two primary outcomes, one each for the pregnancy and postpartum/postnatal periods, respectively. The primary outcome for the pregnancy period is incidence of SGA among live born infants with birth weight measured within 72 hours of delivery. SGA is defined as weight measured within <72 h of delivery <10th centile of the INTERGROWTH 21st reference standard by gestational age and sex.[25] We assessed gestational age using 1st or 2nd trimester ultrasound examination prior to 24 gestational weeks. The primary outcome for

Confidential: For Review Only

the postpartum/postnatal period is mean LAZ at 6 months using the international WHO child growth reference.[26] Secondary outcomes are listed in Table 1.

Sample size

With a sample size of 1,548 woman/infant pairs visited within <72 hours of delivery, we will be able to detect a 17.5% reduction in SGA with 80% power and a 20.2% reduction with 90% power assuming a type 1 error of 5% (two-sided). Since we are assuming no interaction between the marginal effects, this sample size will allow us to detect a difference in infant LAZ at 6 months after delivery of 0.16 Z scores with 80% power and 0.18 Z scores with 90% power. The MINT trial is designed to enroll approximately 1,800 women over a period of 20 months (split into two 6-month enrollment periods). This will yield the required sample of 1,548 woman/infant pairs visited within 72 hours of delivery, after a reduction of 14% due to fetal death and loss to follow-up in pregnancy.

Data collection

All study visits and interviews are conducted in participant households because of the wide dispersion of households across this rural community and the impracticality of bringing participants to a central location (Figure 2). Data collection teams comprising Field Interviewers (FIs) schedule the study visits, administer the questionnaires, and conduct the assessments as described below.

Enrollment visit: FIs conduct the enrollment visit, after notification of a newly identified pregnancy, to administer informed consent, baseline questionnaires, and anthropometry. Questionnaires cover maternal demographic characteristics, socioeconomic status, pregnancy and reproductive history, dietary diversity, and household food security (Household Food Insecurity Access Scale (HFIAS) developed by the Food and Nutrition Technical Assistance).[27] Anthropometric measurements include height (portable stadiometer), weight (MicroLife WS50 scale), mid-upper arm circumference (MUAC) (non-stretch insertion tape), blood pressure, and pulse.

Ultrasound visit: A special cadre of Auxiliary Nurse Midwives (ANMs) conduct the ultrasound assessment for gestational dating by visiting the participant's homes (Supplementary Material, Section A). ANMs are trained to use a portable machine (SonoSite NanoMaxx) and follow the INTERGROWTH 21st methodology for early and late fetal gestational age assessment, including measurement of crown-rump length and/or biparietal diameter (outer-to-outer), head circumference, and femur length.[28, 29]

Weekly morbidity and supplement compliance visits: Data collectors visit each woman weekly during pregnancy starting at 14 weeks based on LMP and in the postpartum period until 6 months after delivery to provide the supplement, collect data on maternal morbidities, and assess supplement compliance.

Late pregnancy visit: FIs conduct a late pregnancy visit between 32-34 gestational weeks to collect data on maternal anthropometry (same measures as enrollment), maternal morbidities,

Confidential: For Review Only

tobacco/alcohol use, ANC utilization, delivery planning, diet diversity, household food security, and depression (using a version of the Edinburgh Postnatal Depression Scale validated in Nepali).[30]

Birth assessment visit: FIs aim to visit each woman within 72 hours of delivery to collection information about the birth and delivery process, early breastfeeding and feeding behaviors, and newborn care practices. Infant weight (g), length (cm), head circumference (cm), and temperature (F) are measured. Weight is measured with digital scales (Tanita BD- 585), length with Shorr-type boards, and MUAC with non-stretch insertion tape. Non-digital measures are taken in triplicate and the median is used for analysis.

Postpartum/Postnatal monthly visits: After the birth visit, field interviewers conduct monthly postnatal visits up to 6 months after delivery to administer questionnaires on maternal and infant morbidity, breastfeeding and infant and young child feeding practices, infant care practices, household food security, and anthropometric measurements for the infant (weight, length, head circumference, MUAC) and mother (weight, MUAC). Additionally, we will visit as many woman/infant pairs at 12 months after delivery as possible until the administrative end of the study to allow us to assess infant growth at 12 months as a secondary aim in a sub-sample.

Biospecimen sub-studies: The MINT study will enroll two sub-studies for biospecimen collection (Supplementary Material, Table 3). First, we will enroll about 120 pregnant women during the first enrollment period from the pregnancy supplementation (n=60) and not groups (n=60). At the enrollment and late pregnancy visits, these women will provide volumetric absorptive microsampling (VAM) samples, for serum metabolomics analyses collected via finger-stick using Neoteryx kits, hemoglobin measurement, a maternal stool sample stored in nucleic acid buffer solution (DNA Genotek OMR-GUT), and their infants will provide a stool sample at 6 months. Second, we will enroll about 120 pregnant women during the second enrollment period from the pregnancy supplementation (n=60) and not groups (n=60), and these women will provide a hemoglobin measurement at the enrollment and late pregnancy visits, and their infants will provide a stool sample at 3 and 6 months.

Dietary intake: Field interviewers will conduct duplicate 24-hour dietary recalls at the late pregnancy and 3-months postpartum visits for a subset of 240 women in the first (n=120) and second (n=120) enrollment periods. The purpose of these data is to assess nutrient intakes and determine whether dietary substitution is occurring in the supplement group. Interviewers use the multiple pass method to help the woman recall all the components of her diet from the previous day. A local food list, photo atlas, and electronic entry form have been adapted from previous work in Nepal to assist with portion size recall and data entry.[31, 32] Daily nutrient intakes will be estimated with local recipes and national food composition tables from India and Nepal.

Bayley Scales of Infant and Toddler development (BSID-4) visit: A specialized team with experience and training in developmental assessment in children will visit a sub-sample of 400 mother/infant pairs to evaluate infant neurodevelopment at 6 months of life.[33, 34] The visit will

Confidential: For Review Only

include the BSID-4 assessment, a standardized evaluation for infants that measures functioning in five areas: cognition, fine motor, gross motor, receptive language, and expressive language. At this visit, data collectors will also conduct the World Health Organization six gross motor milestones module.[35]

Data management
Our field interviewers administer study questionnaires and record anthropometric measurements via digital tablets with a cellular connection using a customized web-based data management system developed on the Microsoft ASP.NET framework. The local data collectors complete paper forms that are recorded by data entry operators on a weekly basis. On the backend, data are stored in a relational database management system (Microsoft SQL Server). Prior to study launch, the field team tests all forms, and restrictions and checks are programmed to reduce data entry errors. The senior field team and co-investigators review important variables weekly to monitor data quality issues, such as missingness, heaping, digit preference, and outliers. The senior field team corrects data entry errors and implements retraining of data collectors on specific protocols when necessary.

Data monitoring
Data and safety monitoring board: An independent Data and Safety Monitoring Board (DSMB) is established for the trial to advise the investigators on issues of safety and efficacy. DSMB membership includes four individuals from Nepal, including a pediatrician, obstetrician-gynecologist, nutritionist, and statistician. The DSMB met prior to the start of the study, twice during implementation of the study, and will meet at least once more.

Serious adverse events: Serious adverse events, as defined under Good Clinical Practice (GCP) guidelines, include death, a life-threatening reaction to the supplement or other study procedure, hospitalization for a reason other than routine delivery, significant or persistent disability or impairment, or a congenital anomaly/birth defect. All data collection staff are trained to recognize a range of serious and other adverse events (AEs). SAEs are investigated and reported to the DSMB and IRBs according to the study research and ethical protocols. Our most senior data collectors conduct verbal autopsies in the case of a maternal or infant death.

Statistical analysis
Primary aim 1: We will undertake an intention-to-treat analysis to examine the effect of supplementation on the first primary outcome of SGA. The analysis will include all live births for whom a birth weight was collected by study data collectors within 72 hours of delivery for the SGA (<10th centile births using the INTERGROWTH-21st standard) outcome. We will assess the balance between the two pregnancy supplementation groups in baseline household- and individual-level characteristics including maternal age, height, body mass index (BMI), parity, previous adverse pregnancy outcome, education, and socioeconomic status. If imbalanced variables are also associated with the primary outcome ($p<0.10$), we will use multivariable log binomial regression modeling to adjust for potential confounding. We will compare the proportion of SGA, between those who were supplemented vs. not according to their allocation status, using a log binomial regression model to estimate the relative risk and 95% confidence

Confidential: For Review Only

interval with the control group as the referent both unadjusted and adjusted for identified confounders. If the final model does not converge, we will use Poisson regression with robust standard error estimation.

Primary aim 2: We will undertake an intention-to-treat analysis to examine the effect of supplementation on the second primary outcome of mean LAZ at 6 months. The analysis will include all children whose length was assessed at 6 months of age. We will assess the balance across the four pregnancy and lactation supplementation groups in baseline household- and individual-level characteristics. If imbalanced variables are also associated with the secondary outcome ($p<0.10$), we will use multivariable linear regression modeling to adjust for potential confounding. Due to the factorial study design, we will assess the interaction between pregnancy and lactation supplementation on the outcome of mean LAZ, although our sample size is not adequate to detect small interaction effects. We will use a multivariable linear regression model with an interaction term for treatment arms. If the interaction is significant (<0.10), we will report both the main and interaction effects. If not significant, which we anticipate, we will report the marginal treatment effects for supplementation during lactation vs. not and during pregnancy vs. not. We will compare mean LAZ scores between supplementation groups using a multivariable linear regression model to estimate the risk difference and 95% confidence interval with the control group as the referent both unadjusted and adjusted for identified confounders.

ETHICS AND DISSEMINATION

Ethical approval

The study was approved by Institutional Review Board (IRB) at Johns Hopkins Bloomberg School of Public Health, Baltimore, USA, (IRB00009714), the Committee on Human Research IRB at The George Washington University, Washington, DC, USA (081739), and the Ethical Review Board of the Nepal Health Research Council, Kathmandu, Nepal (174/2018).

Patient and public involvement

We conducted two formative studies to assess compliance with and acceptability of various formulations of BEP supplements. Our field leadership team also engaged several local stakeholders prior to the start of the study, including municipality mayors, health coordinators, and health facility in-charges in the study area. After completion of the study, the team will conduct similar meetings to disseminate the results.

Ancillary care

The MINT study provides iron folic acid tablets and albendazole to women who report that they have not received these from the health facility during their ANC visits. Women with low hemoglobin (Hb <11g/dL) are referred to a health facility. Women with suspected fetal abnormality detected during the ultrasound gestational age dating visit are referred for clinical examination.

Confidentiality

Confidential: For Review Only

Study investigators and staff treat all data as confidential, and participants are informed that their information will be protected. All data collection forms are kept in secure locations with access limited to relevant study staff. All electronic databases are stored on encrypted, password-protected servers, and data transfer uses secure encrypted transfer protocols. Access to identifying information is restricted to the principal investigator and few key co-investigators and staff. Only anonymized datasets are shared beyond the study team.

Dissemination plan

The study team will disseminate MINT findings to the Sarlahi District Public Health Office, Sarahi District Project Advisory Committee, Ministry of Health and Population, Nepal, Central Project Advisory Committee, and to various local organizations, including Unicef-Nepal and Unicef South Asian Regional Office, USAID, and other stakeholders in Nepal with interests in nutrition during pregnancy and lactation. We will share anonymized data with the Ki data repository and researchers involved in the BEP Harmonization Initiative organized and funded by the BMGF for the purpose of meta-analyses using data from similar trials conducted around the world. The study team will also present results at international workshops and conferences with relevant researchers and program or policy stakeholders. Members of the study team will publish results of primary and secondary analyses in peer-reviewed academic journals.

Confidential: For Review Only

DISCUSSION

Nepal has made progress in reducing maternal, newborn, and child mortality, but a significant burden of undernutrition remains among mothers and infants.[21, 22] Novel and effective interventions and practical implementation strategies are needed to prevent adverse pregnancy outcomes and infant growth faltering,[36, 37] and currently WHO (2016)[13] recommends use of BEP supplements in undernourished settings, but standard formulations of such interventions and data on implementation in programs is lacking. The MINT trial will provide evidence to help refine and implement the WHO context-specific recommendation on BEP supplementation in pregnancy, especially using the fortified, enhanced ready-to-use formulation recommended by the expert consensus on BEP supplements.[16] As multiple micronutrient supplementation (MMS) reduces risk of adverse pregnancy outcomes, including low birth weight and SGA, our trial will contribute evidence as to whether the fortified BEP intervention, which include the same 15 micronutrients, provides additional benefit beyond MMS alone.[38] This trial will generate evidence on the potential of a maternal intervention to prevent early life growth faltering and improve maternal outcomes, such as anemia and inadequate gestational weight gain, areas where a research gap exists. The results from the trial will inform health policy and programs in Nepal, where there currently are no guidelines for community-level management of growth faltering in infants less than 6 months of age beyond exclusive breastfeeding. The primary strength of the trial is the 2x2 factorial RCT design, which will enable understanding of the interconnections between maternal nutritional status in pregnancy and lactation and relationships to fetal and infant growth faltering. The trial includes frequent follow-up visits, over the continuum from early pregnancy to 6 months after delivery, which will generate data on many prenatal and postpartum factors that influence maternal and infant outcomes. The trial will describe maternal dietary intake, assess whether provision of BEP supplements lead to dietary substitution, and measure adherence, yielding practical insights for implementation. Lastly, the trial structure allows for nesting various sub-studies, including those to assess the impact of BEP on the maternal and infant microbiome, infant neurodevelopment, and maternal metabolites.

Confidential: For Review Only

Acknowledgements
We express our sincere thanks to the women and their families in Sarlahi, District, Nepal, who participated in the MINT study. Thank you to Nutriset, France, for producing and donating a portion of the fortified BEP supplements. This study was conducted in collaboration with our implementing partner, Nepal Netra Jyoti Sangh, under the auspices of the Social Welfare Council of the Government of Nepal.

Competing interests
The authors declare no competing interests.

Authors' contributions
JT, JK, LM, and SKK conceptualized the study. All authors contributed to development of the study design, protocols, and forms and tools. SKK, TPL, and SCL led data collection and supervision in the field. DE wrote the first draft of the manuscript. All authors provided revisions and inputs to the manuscript. All authors reviewed and agreed on the final version of the manuscript.

Data availability
The principal investigator will share final anonymized data with the Ki data repository and researchers involved in the BEP Harmonization Initiative for the purpose of meta-analyses using data from similar trials conducted around the world. Data will also be available to other investigators upon reasonable request.

Funding
This study was supported by the Bill & Melinda Gates Foundation (OPP11599195), Thrasher Research Fund (TRF01278), and the Eunice Kennedy Shriver National Institute of Child Health and Human Development (R01HD109385). The funders had no role in the design, implementation, or analysis of the trial.

Confidential: For Review Only

Table 1: MINT study primary and secondary outcomes

**Primary outcomes**	
Small-for-gestational age (10th centile)	Proportion of infants with birth weight <10th centile of INTERGROWTH 21st reference standard by gestational age and sex for infants measured <72 hours after delivery.
Length-for-age Z (LAZ)	Mean infant length-for-age Z score at 6 months of age using the World Health Organization Child Growth Study criteria.
**Secondary outcomes**	
**Newborn**	
Small-for-gestational-age (3rd centile)	Proportion of infants with birth weight <3rd centile of INTERGROWTH 21st reference standard by gestational age and sex for infants measured <72 hours after delivery.
Large-for-gestational-age (90th centile)	Proportion of infants with birth weight >90th centile of INTERGROWTH 21st reference standard by gestational age and sex for infants measured <72 hours after delivery.
Short-for-gestational-age (10th centile)	Proportion of infants with birth length <10th centile of INTERGROWTH 21st reference standard by gestational age and sex for infants measured <72 hours after delivery.
Short-for-gestational-age (3rd centile)	Proportion of infants with birth length <3rd centile of INTERGROWTH 21st reference standard by gestational age and sex for infants measured <72 hours after delivery.
Birth weight	Mean infant birth weight.
Birth length	Mean infant birth length.
Head circumference	Mean infant head circumference
Arm circumference	Mean infant mid-upper arm circumference
Low birth weight	Proportion of infants with birth weight <2,500g for infants measured <72 hours after delivery.
Gestational age	Mean infant gestational age at delivery.
Preterm birth (<37 weeks)	Proportion of pregnancies that are <37 gestational weeks using the INTERGROWTH 21st standards for 1st and 2nd trimester ultrasound dating.
Very, extremely preterm (<34, <32, <28 weeks)	Proportion of pregnancies that are <34, <32, <28 gestational weeks using the INTERGROWTH 21st standards for 1st and 2nd trimester ultrasound dating.
Stillbirth	Proportion of pregnancies resulting in stillbirth, defined as fetal death ≥28 gestational weeks, using the INTERGROWTH 21st standards for 1st and 2nd trimester ultrasound dating.

Confidential: For Review Only

Weight-for-length Z score at 6 months of age	Mean infant weight-for-length Z score at 6 months of age using the World Health Organization Child Growth Study criteria.
Weight-for-age Z score at 6 months of age	Mean infant weight-for-age Z score at 6 months of age using the World Health Organization Child Growth Study criteria.
Stunting	Proportion of infants with <-2 length-for-age Z score at 6 months of age using the World Health Organization Child Growth Study criteria.
Wasting	Proportion of infants with <-2 weight-for-length Z score at 6 months of age using the World Health Organization Child Growth Study criteria.
Underweight	Proportion of infants with <-2 weight-for-age Z score at 6 months of age using the World Health Organization Child Growth Study criteria.
Developmental scales	Bayley Scales of Infant and Toddler Development 4 (BSID-4) assessment at 6 months.
Gross motor milestones	World Health Organization 6 gross motor milestones at 6 months.
Gut microbiome	Gut microbiome diversity and stool metabolomics at 3, 6 months.
**Maternal**	
Gestational weight gain	Mean maternal weight gain from enrollment to late pregnancy visits.
Net gestational weight gain	Mean maternal weight gain from enrollment to maternal birth assessment visit (after delivery).
Inadequate maternal gestational weight gain	Proportion of women with inadequate rate of gestational weight gain from enrollment to late pregnancy visit using the INTERGROWTH 21st reference for normal body mass index (BMI) and IOM reference for low and high BMI.
Inadequate maternal net gestational weight gain	Proportion of women with inadequate rate of gestational weight gain from enrollment to maternal birth assessment visit using the INTERGROWTH 21st reference for normal body mass index (BMI) and IOM reference for low and high BMI.
Mid-upper arm circumference	Mean change in mid-upper arm circumference from enrollment to late pregnancy visits.
Anemia	Proportion of women who are anemic (Hb <110 g/L) in late pregnancy.
Weight at 6 months postpartum	Mean maternal weight at 6 months postpartum.
Maternal dietary intake	Mean maternal dietary intake score in late pregnancy and 3 months postpartum.
Minimum maternal dietary intake	Proportion of women meeting the minimum dietary intake score in late pregnancy and 3 months postpartum.

Confidential: For Review Only

Gut microbiome	Gut microbiome diversity and stool metabolomics during pregnancy.
Metabolites	Metabolites in the blood during pregnancy.

Confidential: For Review Only

References

1. Kozuki N, Katz J, Lee AC, et al. Short Maternal Stature Increases Risk of Small-for-Gestational-Age and Preterm Births in Low- and Middle-Income Countries: Individual Participant Data Meta-Analysis and Population Attributable Fraction. J Nutr. 2015;145(11):2542-50.
2. Christian P, Lee SE, Donahue Angel M, et al. Risk of childhood undernutrition related to small-for-gestational age and preterm birth in low- and middle-income countries. Int J Epidemiol. 2013;42(5):1340-55.
3. Murray E, Fernandes M, Fazel M, et al. Differential effect of intrauterine growth restriction on childhood neurodevelopment: a systematic review. Bjog. 2015;122(8):1062-72.
4. Victora CG, Adair L, Fall C, et al. Maternal and child undernutrition: consequences for adult health and human capital. Lancet. 2008;371(9609):340-57.
5. Lawn J, Ohuma E, Bradley E, et al. Small babies, big risks: Global estimates of prevalence and mortality for vulnerable newborns to accelerate change and improve counting. Lancet. 2023.
6. Erchick DJ, Hazel EA, Katz J, et al. Vulnerable newborn types: analysis of subnational, population-based birth cohorts for 541 285 live births in 23 countries, 2000-2021. Bjog. 2023.
7. Katz J, Lee AC, Kozuki N, et al. Mortality risk in preterm and small-for-gestational-age infants in low-income and middle-income countries: a pooled country analysis. Lancet. 2013;382(9890):417-25.
8. Lee AC, Kozuki N, Cousens S, et al. Estimates of burden and consequences of infants born small for gestational age in low and middle income countries with INTERGROWTH-21(st) standard: analysis of CHERG datasets. Bmj. 2017;358:j3677.
9. Benjamin-Chung J, Mertens A, Colford JM, et al. Early childhood linear growth failure in low- and middle-income countries. medRxiv. 2020:2020.06.09.20127001.
10. Christian P. Prenatal origins of malnutrition. Emerging Societies Coexistence of Childhood Malnutrition and Obesity. 632009.
11. Black RE, Victora CG, Walker SP, et al. Maternal and child undernutrition and overweight in low-income and middle-income countries. Lancet. 2013;382(9890):427-51.
12. Keusch GT, Rosenberg IH, Denno DM, et al. Implications of acquired environmental enteric dysfunction for growth and stunting in infants and children living in low- and middle-income countries. Food Nutr Bull. 2013;34(3):357-64.
13. World Health Organization. WHO Guidelines Approved by the Guidelines Review Committee. WHO Recommendations on Antenatal Care for a Positive Pregnancy Experience. Geneva: World Health Organization; 2016.
14. Ota E, Hori H, Mori R, et al. Antenatal dietary education and supplementation to increase energy and protein intake. Cochrane Database Syst Rev. 2015(6):Cd000032.
15. Imdad A, Bhutta ZA. Maternal nutrition and birth outcomes: effect of balanced protein-energy supplementation. Paediatr Perinat Epidemiol. 2012;26 Suppl 1:178-90.
16. Members of an Expert Consultation on Nutritious Food Supplements for Pregnant and Lactating Women. Framework and specifications for the nutritional composition of a food supplement for pregnant and lactating women (PLW) in undernourished and low income settings. . Gates Open Res. 2019;3:1498 (document)
17. Subedi S, Hazel EA, Mohan D, et al. Prevalence and predictors of spontaneous preterm births in Nepal: findings from a prospective, population-based pregnancy cohort in rural Nepal-a secondary data analysis. BMJ Open. 2022;12(12):e066934.
18. Bryce E, Gurung S, Tong H, et al. Population attributable fractions for risk factors for spontaneous preterm births in 81 low- and middle-income countries: A systematic analysis. J Glob Health. 2022;12:04013.

Confidential: For Review Only

19. Hazel EA, Mohan D, Zeger S, et al. Demographic, socio-economic, obstetric, and behavioral factors associated with small-and large-for-gestational-age from a prospective, population-based pregnancy cohort in rural Nepal: a secondary data analysis. BMC Pregnancy Childbirth. 2022;22(1):652.
20. Chan AW, Tetzlaff JM, Altman DG, et al. SPIRIT 2013 statement: defining standard protocol items for clinical trials. Ann Intern Med. 2013;158(3):200-7.
21. Ministry of Health - MOH/Nepal, New ERA/Nepal, ICF. Nepal Demographic and Health Survey 2016. Kathmandu, Nepal: MOH/Nepal, New ERA, and ICF; 2017.
22. Ministry of Health - MOH/Nepal, New ERA/Nepal, ICF. Nepal Demographic and Health Survey Key Indicators Report 2022. Kathmandu, Nepal: MOH/Nepal, NEW ERA, and ICF; 2022.
23. Lama TP, Khatry SK, Isanaka S, et al. Acceptability of 11 fortified balanced energy-protein supplements for pregnant women in Nepal. Matern Child Nutr. 2022;18(3):e13336.
24. Lama TP, Moore K, Isanaka S, et al. Compliance with and acceptability of two fortified balanced energy protein supplements among pregnant women in rural Nepal. Matern Child Nutr. 2022;18(2):e13306.
25. Villar J, Cheikh Ismail L, Victora CG, et al. International standards for newborn weight, length, and head circumference by gestational age and sex: the Newborn Cross-Sectional Study of the INTERGROWTH-21st Project. Lancet. 2014;384(9946):857-68.
26. WHO Child Growth Standards based on length/height, weight and age. Acta Paediatr Suppl. 2006;450:76-85.
27. Coates J, Swindale, A, Bilinsky P. Household Food Insecurity Access Scale (HFIAS) for Measurement of Household Food Access: Indicator Guide (v. 3). Washington, D.C.: FHI 360/FANTA; 2007.
28. Papageorghiou AT, Kennedy SH, Salomon LJ, et al. International standards for early fetal size and pregnancy dating based on ultrasound measurement of crown–rump length in the first trimester of pregnancy. Ultrasound in Obstetrics & Gynecology. 2014;44(6):641-8.
29. Papageorghiou AT, Kemp B, Stones W, et al. Ultrasound-based gestational-age estimation in late pregnancy. Ultrasound Obstet Gynecol. 2016;48(6):719-26.
30. Bhusal BR, Bhandari N, Chapagai M, et al. Validating the Edinburgh Postnatal Depression Scale as a screening tool for postpartum depression in Kathmandu, Nepal. Int J Ment Health Syst. 2016;10:71-.
31. Harris-Fry H, Paudel P, Karn M, et al. Development and validation of a photographic food atlas for portion size assessment in the southern plains of Nepal. Public Health Nutr. 2016;19(14):2495-507.
32. Harris-Fry H, Beard BJ, Harrisson T, et al. Smartphone tool to collect repeated 24 h dietary recall data in Nepal. Public Health Nutr. 2018;21(2):260-72.
33. Murray-Kolb LE, Rasmussen ZA, Scharf RJ, et al. The MAL-ED cohort study: methods and lessons learned when assessing early child development and caregiving mediators in infants and young children in 8 low- and middle-income countries. Clin Infect Dis. 2014;59 Suppl 4(Suppl 4):S261-72.
34. Ranjitkar S, Kvestad I, Strand TA, et al. Acceptability and Reliability of the Bayley Scales of Infant and Toddler Development-III Among Children in Bhaktapur, Nepal. Front Psychol. 2018;9:1265.
35. Wijnhoven TM, de Onis M, Onyango AW, et al. Assessment of gross motor development in the WHO Multicentre Growth Reference Study. Food Nutr Bull. 2004;25(1 Suppl):S37-45.
36. Bhutta ZA, Das JK, Rizvi A, et al. Evidence-based interventions for improvement of maternal and child nutrition: what can be done and at what cost? Lancet. 2013;382(9890):452-77.
37. Bhutta ZA, Das JK, Bahl R, et al. Can available interventions end preventable deaths in mothers, newborn babies, and stillbirths, and at what cost? Lancet. 2014;384(9940):347-70.

Confidential: For Review Only

38. Keats EC, Haider BA, Tam E, et al. Multiple-micronutrient supplementation for women during pregnancy. Cochrane Database Syst Rev. 2019;3(3):Cd004905.

Confidential: For Review Only

Figure 1: Study design of MINT 2x2 factorial randomized trial

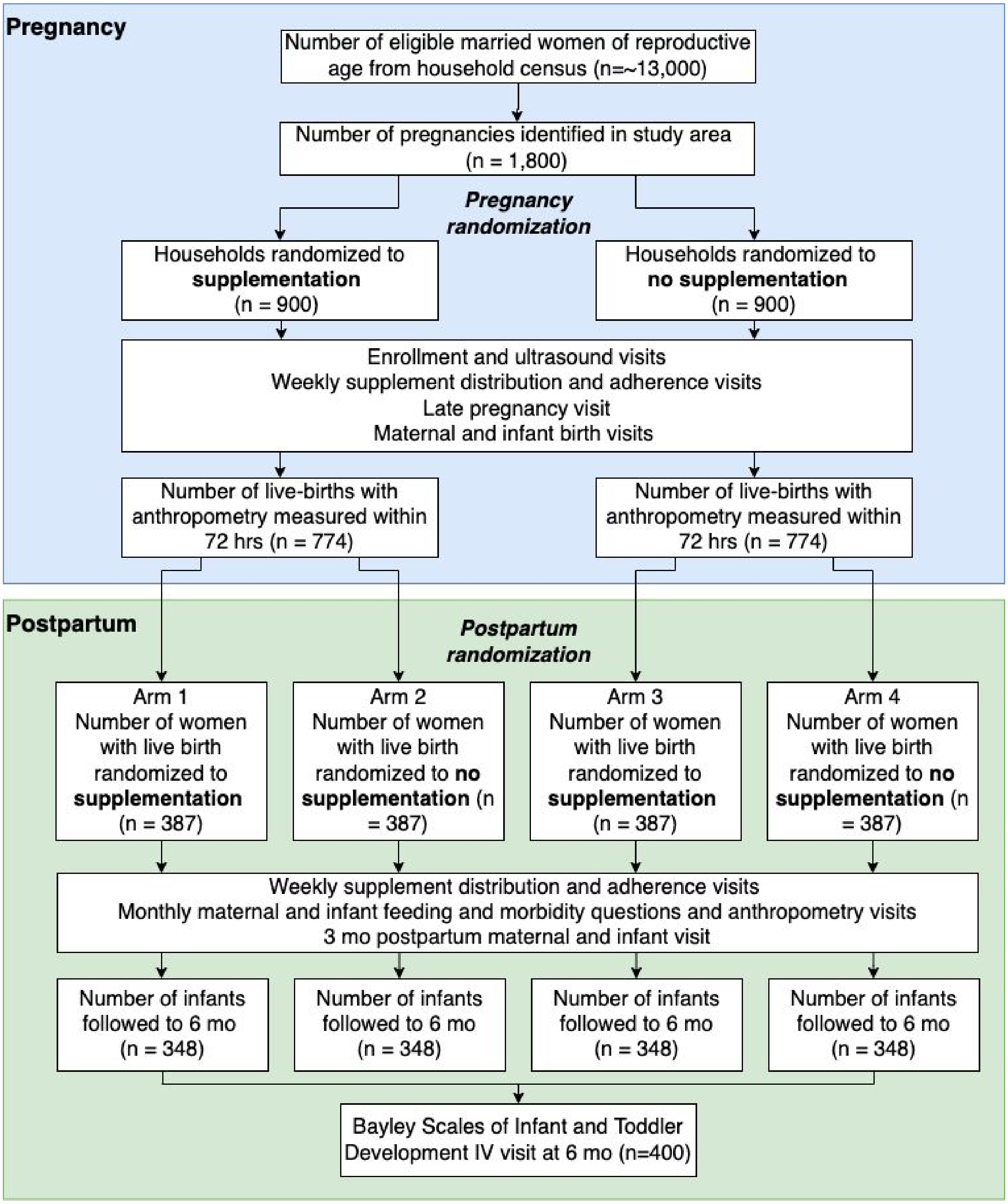

Figure 2: Study procedures flow diagram

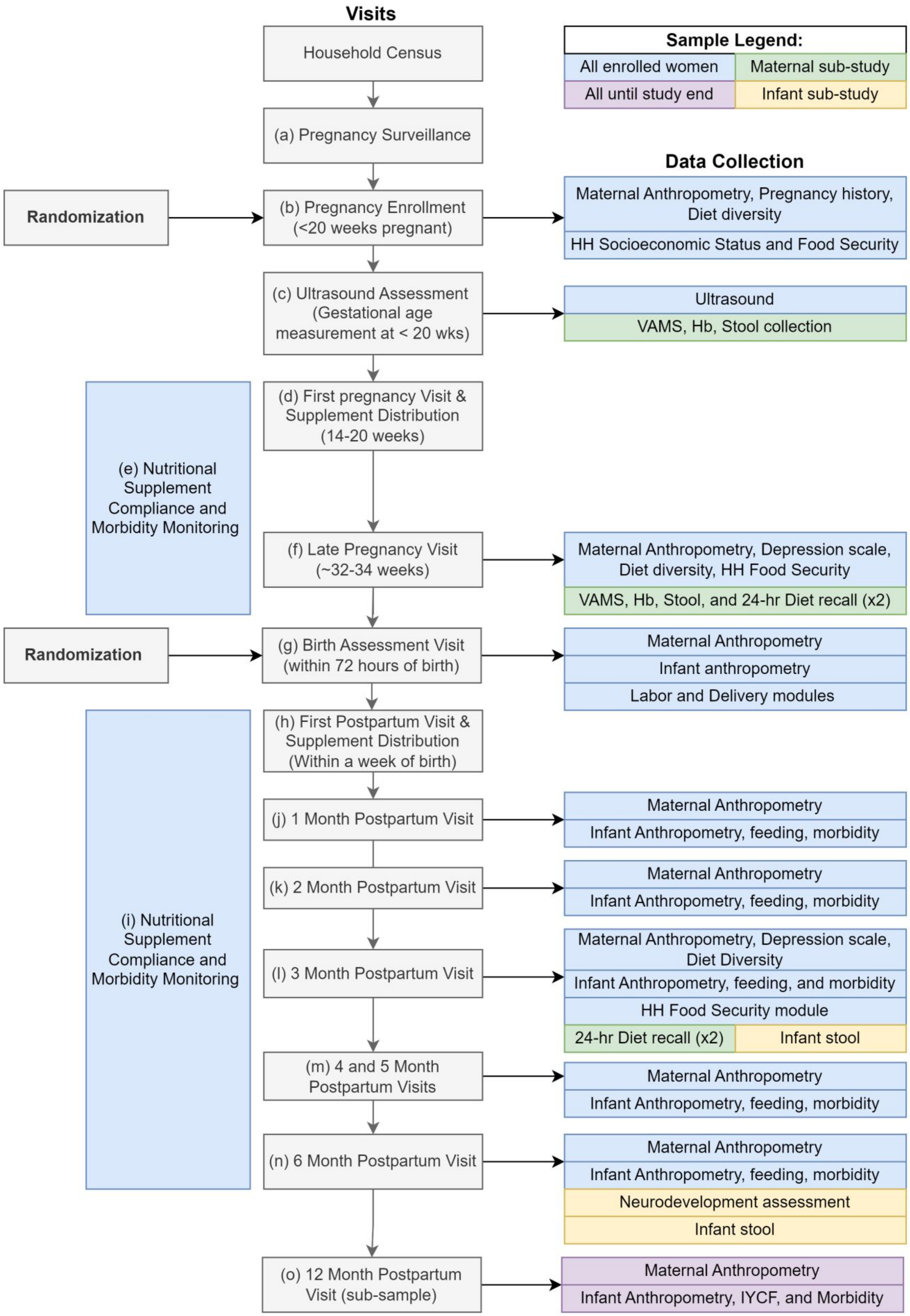

SUPPLEMENTARY MATERIAL

Table 1: Standard protocol items: recommendations for interventional trials (SPIRIT) guidelines checklist

SPIRIT 2013 Checklist: Recommended items to address in a clinical trial protocol and related documents*

**Section/item**	**Item No**	**Description**	**Addressed on page number**
**Administrative information**			
Title	1	Descriptive title identifying the study design, population, interventions, and, if applicable, trial acronym	_____1______
Trial registration	2a	Trial identifier and registry name. If not yet registered, name of intended registry	_____2______
	2b	All items from the World Health Organization Trial Registration Data Set	_____2______
Protocol version	3	Date and version identifier	_____2______
Funding	4	Sources and types of financial, material, and other support	____15______
Roles and responsibilities	5a	Names, affiliations, and roles of protocol contributors	_____1______
	5b	Name and contact information for the trial sponsor	_____15_____

Confidential: For Review Only

	5c	Role of study sponsor and funders, if any, in study design; collection, management, analysis, and interpretation of data; writing of the report; and the decision to submit the report for publication, including whether they will have ultimate authority over any of these activities	_____15______
	5d	Composition, roles, and responsibilities of the coordinating centre, steering committee, endpoint adjudication committee, data management team, and other individuals or groups overseeing the trial, if applicable (see Item 21a for data monitoring committee)	______NA_____

Introduction

Background and rationale	6a	Description of research question and justification for undertaking the trial, including summary of relevant studies (published and unpublished) examining benefits and harms for each intervention	______4______
	6b	Explanation for choice of comparators	______4______
Objectives	7	Specific objectives or hypotheses	_____4-5______
Trial design	8	Description of trial design including type of trial (eg, parallel group, crossover, factorial, single group), allocation ratio, and framework (eg, superiority, equivalence, noninferiority, exploratory)	_____4-5______

Confidential: For Review Only

Methods: Participants, interventions, and outcomes

Study setting	9	Description of study settings (eg, community clinic, academic hospital) and list of countries where data will be collected. Reference to where list of study sites can be obtained	_____6______
Eligibility criteria	10	Inclusion and exclusion criteria for participants. If applicable, eligibility criteria for study centres and individuals who will perform the interventions (eg, surgeons, psychotherapists)	_____7______
Interventions	11a	Interventions for each group with sufficient detail to allow replication, including how and when they will be administered	_____8______
	11b	Criteria for discontinuing or modifying allocated interventions for a given trial participant (eg, drug dose change in response to harms, participant request, or improving/worsening disease)	_____8______
	11c	Strategies to improve adherence to intervention protocols, and any procedures for monitoring adherence (eg, drug tablet return, laboratory tests)	____9-10_____
	11d	Relevant concomitant care and interventions that are permitted or prohibited during the trial	_____8______

Outcomes	12	Primary, secondary, and other outcomes, including the specific measurement variable (eg, systolic blood pressure), analysis metric (eg, change from baseline, final value, time to event), method of aggregation (eg, median, proportion), and time point for each outcome. Explanation of the clinical relevance of chosen efficacy and harm outcomes is strongly recommended	______8______
Participant timeline	13	Time schedule of enrolment, interventions (including any run-ins and washouts), assessments, and visits for participants. A schematic diagram is highly recommended (see Figure)	__9-10, 16____
Sample size	14	Estimated number of participants needed to achieve study objectives and how it was determined, including clinical and statistical assumptions supporting any sample size calculations	____8-9______
Recruitment	15	Strategies for achieving adequate participant enrolment to reach target sample size	____6-7______

Methods: Assignment of interventions (for controlled trials)

Allocation:

ly

Sequence generation	16a	Method of generating the allocation sequence (eg, computer-generated random numbers), and list of any factors for stratification. To reduce predictability of a random sequence, details of any planned restriction (eg, blocking) should be provided in a separate document that is unavailable to those who enrol participants or assign interventions	____7-8______
Allocation concealment mechanism	16b	Mechanism of implementing the allocation sequence (eg, central telephone; sequentially numbered, opaque, sealed envelopes), describing any steps to conceal the sequence until interventions are assigned	____7-8______
Implementatio n	16c	Who will generate the allocation sequence, who will enrol participants, and who will assign participants to interventions	____7-8______
Blinding (masking)	17a	Who will be blinded after assignment to interventions (eg, trial participants, care providers, outcome assessors, data analysts), and how	_____8______
	17b	If blinded, circumstances under which unblinding is permissible, and procedure for revealing a participant's allocated intervention during the trial	____NA______

Methods: Data collection, management, and analysis

Data collection methods	18a	Plans for assessment and collection of outcome, baseline, and other trial data, including any related processes to promote data quality (eg, duplicate measurements, training of assessors) and a description of study instruments (eg, questionnaires, laboratory tests) along with their reliability and validity, if known. Reference to where data collection forms can be found, if not in the protocol	___9-10_____
	18b	Plans to promote participant retention and complete follow-up, including list of any outcome data to be collected for participants who discontinue or deviate from intervention protocols	___9-10______
Data management	19	Plans for data entry, coding, security, and storage, including any related processes to promote data quality (eg, double data entry; range checks for data values). Reference to where details of data management procedures can be found, if not in the protocol	_____11_____
Statistical methods	20a	Statistical methods for analysing primary and secondary outcomes. Reference to where other details of the statistical analysis plan can be found, if not in the protocol	____11-12____
	20b	Methods for any additional analyses (eg, subgroup and adjusted analyses)	___11-12_____
	20c	Definition of analysis population relating to protocol non-adherence (eg, as randomised analysis), and any statistical methods to handle missing data (eg, multiple imputation)	___11-12_____

Methods: Monitoring

Data monitoring	21a	Composition of data monitoring committee (DMC); summary of its role and reporting structure; statement of whether it is independent from the sponsor and competing interests; and reference to where further details about its charter can be found, if not in the protocol. Alternatively, an explanation of why a DMC is not needed	____11______
	21b	Description of any interim analyses and stopping guidelines, including who will have access to these interim results and make the final decision to terminate the trial	____NA______
Harms	22	Plans for collecting, assessing, reporting, and managing solicited and spontaneously reported adverse events and other unintended effects of trial interventions or trial conduct	____11______
Auditing	23	Frequency and procedures for auditing trial conduct, if any, and whether the process will be independent from investigators and the sponsor	____NA______

Ethics and dissemination

Research ethics approval	24	Plans for seeking research ethics committee/institutional review board (REC/IRB) approval	____12______

w Only

Protocol amendments	25	Plans for communicating important protocol modifications (eg, changes to eligibility criteria, outcomes, analyses) to relevant parties (eg, investigators, REC/IRBs, trial participants, trial registries, journals, regulators)	____12______
Consent or assent	26a	Who will obtain informed consent or assent from potential trial participants or authorised surrogates, and how (see Item 32)	_____9______
	26b	Additional consent provisions for collection and use of participant data and biological specimens in ancillary studies, if applicable	_____NA_____
Confidentiality	27	How personal information about potential and enrolled participants will be collected, shared, and maintained in order to protect confidentiality before, during, and after the trial	___12-13_____
Declaration of interests	28	Financial and other competing interests for principal investigators for the overall trial and each study site	_____15_____
Access to data	29	Statement of who will have access to the final trial dataset, and disclosure of contractual agreements that limit such access for investigators	_____15_____
Ancillary and post-trial care	30	Provisions, if any, for ancillary and post-trial care, and for compensation to those who suffer harm from trial participation	_____NA_____

Confidential: For Revi

Dissemination policy	31a	Plans for investigators and sponsor to communicate trial results to participants, healthcare professionals, the public, and other relevant groups (eg, via publication, reporting in results databases, or other data sharing arrangements), including any publication restrictions	_____13_____
	31b	Authorship eligibility guidelines and any intended use of professional writers	_____15_____
	31c	Plans, if any, for granting public access to the full protocol, participant-level dataset, and statistical code	_____15_____
**Appendices**			
Informed consent materials	32	Model consent form and other related documentation given to participants and authorised surrogates	__Section C___
Biological specimens	33	Plans for collection, laboratory evaluation, and storage of biological specimens for genetic or molecular analysis in the current trial and for future use in ancillary studies, if applicable	10, main manuscript

*It is strongly recommended that this checklist be read in conjunction with the SPIRIT 2013 Explanation & Elaboration for important clarification on the items. Amendments to the protocol should be tracked and dated. The SPIRIT checklist is copyrighted by the SPIRIT Group under the Creative Commons "Attribution-NonCommercial-NoDerivs 3.0 Unported" license.

Confidential: For Review Only

Table 2: Balanced energy-protein supplement nutritional composition

Formula 1 ingredients: Vegetable oils (rapeseed, palm, soy in varying proportions), defatted soy flour, skimmed milk powder, peanuts, sugar, maltodextrin, soy protein isolate, vitamin and mineral complex, stabilizer (fully hydrogenated vegetable fat, mono and diglycerides). Allergens warning: peanuts, soy and dairy products. May contain traces of gluten.

Formula 2 ingredients: Vegetable oils (rapeseed, palm), skimmed milk powder, defatted soy flour, peanuts, sugar, maltodextrin, vitamin and mineral premix, fully hydrogenated vegetable oil (palm). Allergens warning: peanuts, soy and dairy products. May contain traces of gluten.

Formula 3 ingredients: Peanuts, vegetable oil (rapeseed), skimmed milk powder, sugar, whey protein concentrate, maltodextrin, premix of vitamins and minerals, fully hydrogenated vegetable oil (palm), flavor. Allergens warning: peanuts and dairy products. May contain traces of soy.

		**Formula 1**		**Formula 2**		**Formula 3**	
		**RUSF P&L 72g**		**Reformulation 1 RUSF P&L 72g**		**Reformulation RUSF 2 P&L 72**	
**Nutrients**	**Unit**	Mean / 100g	Mean / dose 72g	Mean / 100g	Mean / dose 72g	Mean / 100g	Mean / dose 72g
**Energy**	**kcal**	546	393	544	391	557	401
**Protein**	**g**	20.2	14.5	20.4	14.7	20.2	14.5
**Lipids**	**g**	36	26	36	26	37.3	26.9
**Linoleic Acid**	**g**	5.4	3.9	5.4	3.9	6.2	4.5
**alpha-Linolenic Acid**	**g**	1.8	1.3	1.9	1.4	1.8	1.3
**Calcium**	**mg**	694	500	776	559	695	500
**Zinc**	**mg**	21	15	21	15	21	15
**Copper**	**mg**	1.8	1.3	1.8	1.3	1.8	1.3

Confidential: For Review Only

**Iron**	**mg**	31	22	30	22	31	22
**Iodine**	**ug**	347	250	360	260	348	250
**Selenium**	**ug**	90	65	91	65	94	68
**Manganese**	**mg**	2.9	2.1	2.9	2.1	2.9	21
**Magnesium**	**mg**	102	73	108	78	99	72
**Free phosphorus**	**mg**	580	418	638	459	664	478
**Potassium**	**mg**	781	562	892	642	800	576
**Vitamin A**	**ug RE**	1069	770	1069	770	1,069	770
**Vitamin B1**	**mg**	1.9	1.4	1.9	1.4	1.9	1.4
**Vitamin B2**	**mg**	1.9	1.4	1.9	1.4	1.9	1.4
**Niacin**	**mg**	21	15	21	15	21	15
**Vitamin B5**	**mg**	9.7	7	9.7	7	9.7	7.0
**Vitamin B6**	**mg**	2.6	1.9	2.6	1.9	2.6	1.9
**Folic Acid**	**ug**	556	400	556	400	556	400
**Vitamin B12**	**ug**	3.6	2.6	3.6	2.6	3.6	2.6
**Vitamin C**	**mg**	139	100	139	100	139	100
**Vitamin D (Cholecacifero l)**	**ug**	21	15	21	15	21	15

Confidential: For Review Only

**Vitamin E (alpha tocopherol)**	**mg**	25	18	25	18	25	16
**Vitamin K**	**ug**	100	72	100	72	100	72

Confidential: For Review Only

Table 3: Summary of sub-study enrollments
This study has three sub-cohorts: two biospecimen cohorts and a neurodevelopment sub-study cohort.

	1st biospecimen sub- study (n=100 women/infant pairs)	2nd biospecimen sub- study (n=120 study women/infant pairs)	Neurodevelopment sub-study (n=400 infants) women/infant pairs)
**Hemoglobin (early and late pregnancy)**	100	120	N/A
**VAMS (early and late pregnancy)**	100	0	N/A
**Maternal stool (early and late pregnancy)**	100	0	N/A
**Infant stool**	100 (6 months)	120 (3, 6 months)	N/A
**Dietary 24 recall visit (2x visits in pregnancy and 3-months postpartum)**	100	120	N/A
**Neurodevelopment assessment visit at 6 months postnatal**	N/A	N/A	400

Confidential: For Review Only

Section A: Ultrasound gestational age dating protocol

Purpose: The purpose of the ultrasound exam is to collect data on fetal measurements using transabdominal ultrasound to estimate gestational age. Gestational age is required for calculation of our primary outcome, incidence of small-for-gestational-age (<10th centile) among live born infants whose birthweight is measured within 72 hours of delivery. Gestational age is also required construction of secondary outcomes, such as preterm birth.

Staff: The ultrasound gestational age exam is conducted by a specialized team of Auxiliary Nurse Midwives (ANMs) and assistants in this study. Previously, ANMs in our field team have undergone extensive training for in-home ultrasound examination to identify non-cephalic presentation, multiple births, placenta previa, and transabdominal ultrasound exam for gestational age dating. These trainings have been conducted by staff at the Department of Radiology at Tribhuvan University in Kathmandu. ANMs in this study receive similar training and oversight.

Measurements: The MINT study schedules participants for the ultrasound exam as soon as possible after identification of a pregnancy through our pregnancy surveillance system and the enrollment visit. The study aims to complete the ultrasound exam as earlier as feasible after pregnancy identification, ideally in the first trimester, for all participants to ensure the most accurate results. As part of the ultrasound exam, ANMs measure and record several measurements, depending upon the gestation at time of the exam, including crown rump length (CRL), biparietal diameter (BPD), head circumference (HC), and femur length (FL). The measurements and calculations follow the INTERGROWTH 21st methodology for early and late fetal gestational age assessment (Papageorghiou 2014, Papageorghiou 2016). In brief, the ANMs first attempt to measure CRL. If CRL is <15 mm, participants are rescheduled for another ultrasound exam in the following weeks. If CRL is ≥15 mm and <85 mm, the ANM will measure and record three CRL measurements. If CRL is ≥85 to ≤95 mm, in addition to CRL, the ANM also measure and record three values for BPD, HC, and FL. If CRL is >95 mm or CRL cannot be measured because the fetus is too large to fit in the field of view, the ANM will only measure and record three values for BPD, HC, and FL. Biparental diameter is measured “outer-to-outer.” The median measure for each anatomical feature is used for calculation of gestational age using the INTERGROWTH 21st formulas (Papageorghiou 2014, Papageorghiou 2016). Suspected abnormalities are recorded for review and referral by the study radiologist.

Quality control: ANMs record measurements and save digital images from the exam for review by senior field staff and a radiologist who are not present at the home visit. The radiologist reviews a 10-15% sample of images collected by the ANMs to validate that correct imaging and measurement techniques are used for each anatomical feature following the INTERGROWTH-21st Study Protocol (International Fetal and Newborn Growth Consortium 2009).

Equipment: We use the Sonosite NanoMaxx ultrasound machine for our study. The machine is FDA approved, and the specifications are as follows:

Confidential: For Review Only

- Product: Nanomaxx ultrasound machine
- Weight: 6 lbs / 2.7 kg (with battery)
- Dimensions: 14.1" L x 8.2" H x 2.3" W / 35.8 cm L x 20.8 cm H x 5.8 cm W
- Display: 8.4" / 21.3 cm diagonal display LCD touch screen
- Architecture: All-digital broadband
- Dynamic Range: Up to 165 dB
- Gray Scale: 200 dpi (voltage axis) x 500 dpi (time axis)
- HIPAA compliance: Comprehensive tool set
- Manufacturer: Sonosite

References:

Papageorghiou AT, Kennedy SH, Salomon LJ, Ohuma EO, Cheikh Ismail L, Barros FC, Lambert A, Carvalho M, Jaffer YA, Bertino E, Gravett MG, Altman DG, Purwar M, Noble JA, Pang R, Victora CG, Bhutta ZA, Villar J; International Fetal and Newborn Growth Consortium for the 21st Century (INTERGROWTH-21st). International standards for early fetal size and pregnancy dating based on ultrasound measurement of crown-rump length in the first trimester of pregnancy. Ultrasound Obstet Gynecol. 2014 Dec;44(6):641-8.

Papageorghiou AT, Kemp B, Stones W, Ohuma EO, Kennedy SH, Purwar M, Salomon LJ, Altman DG, Noble JA, Bertino E, Gravett MG, Pang R, Cheikh Ismail L, Barros FC, Lambert A, Jaffer YA, Victora CG, Bhutta ZA, Villar J; International Fetal and Newborn Growth Consortium for the 21st Century (INTERGROWTH-21st). Ultrasound-based gestational-age estimation in late pregnancy. Ultrasound Obstet Gynecol. 2016 Dec;48(6):719-726.

International Fetal and Newborn Growth Consortium. The International Fetal and Newborn Growth Standards for the 21st Century (INTERGROWTH-21st) Study Protocol, 2009, www.intergrowth21.org.uk

Confidential: For Review Only

Section B: Informed consent document

Nepal Pregnancy and Lactation Nutritional Supplementation Project

Consent for Participation in the Phase 3 Study

GREETING: Namaste! I am a Nepal Nutrition Intervention Project-Sarlahi (NNIPS) staff person. As you may know, NNIPS has been doing research projects concerning the health of mothers and children in your community for the past 30 years. Today, I would like to talk with you about a new NNIPS project. This project is being jointly run by NNIPS, Nepal Netra Jyoti Sangh, George Washington University, and Johns Hopkins University in the USA. I would like to tell you more this new project, ask for your consent to take part in it, and answer any questions you may have about the project.

PURPOSE

We are doing this new research study to learn more about nutrition for pregnant and breastfeeding women in our community. Specifically, we would like to learn if adding a special diet supplement (snack created to meet the needs of pregnant women in this area) to the diet of pregnant and breastfeeding women will improve the health and growth of their newborns and young babies. We would like to compare women who do and don't use this special diet supplement along with their normal diet. We are asking all pregnant women aged 15-30 in this community if they would like to participate in this study, until about 1800 women have agreed to be in the study. This study may help inform the government about ways to improve the health of babies and young infants in Nepal.

PROCEDURES

If you choose to take part in this project, I will ask you some questions today about your household and your family, and also ask about your previous pregnancies. I will also ask you about your current pregnancy, your health and diet, and measure your height, weight, and your blood pressure. This visit will take approximately 30-45 minutes. I will also schedule you to have an ultrasound examination by one of our Auxiliary Nurse-Midwives to check on how far along in pregnancy you are and to take a finger stick blood sample to measure your hemoglobin level. Once you are more than 14 weeks after your last period you will be asked to do one of 4 activities:

- Keep diet and level of activity as you normally would during your pregnancy and while you are breastfeeding.
- Take a special supplement to your diet while you are pregnant but then resume your regular diet after having your baby while you are breastfeeding.
- Keep your regular diet while you are pregnant, but take a special supplement to your diet during the first 6 months after having your baby, while you are breastfeeding.
- Take a special supplement to your diet while you are pregnant and after you have your baby for the first 6 months while you are breastfeeding.

You and the other pregnant women in your neighborhood will be assigned to one of these activities by chance. It is very important that you take the nutritional supplement every day if you are assigned to a supplement group and that you don't change your regular diet.

Confidential: For Review Only

I or another NNIPS worker will visit you to provide you with these special supplements and ask about your use of the supplements on a weekly basis. These visits will only take about 10 minutes.

Once late in your pregnancy, I or another NNIPS worker will visit to ask about your health during your pregnancy and to measure your weight and blood pressure.

We will visit you on the day your baby is born and ask some questions about the delivery and the health of you and your baby. We will also measure your baby's size and temperature. This visit will take about 30 minutes. We will continue to visit weekly until your baby is 6 months old. Once a month for 6 months, we will measure your baby's weight and length and your weight and ask some questions about the baby's and your health. These visits will take about 25 minutes. For some women and their infants, we will visit again when your baby is 12 months old to measure your baby's weight and length and your weight.

Altogether, you will be in this study for about 14 months.

RISKS/DISCOMFORTS

If you are allergic to milk, nuts (including peanuts) or soy you should not participate in this study. There are no significant risks to you or your baby if you take part in this study. There are minor risks to your privacy, but we will work hard to keep your information and participation confidential. You may feel uncomfortable with some of the questions we ask. If you feel at all uncomfortable with any of the questions we ask, you can just skip those questions. In the short interviews today and during the follow up visits, if there are any questions that are sensitive, you do not have to answer. You may experience slight pain in your finger when we take the blood sample.

As you know, proper nutrition in pregnancy is important for the health of babies in our community. While there are many potential benefits of better nutrition in pregnancy, it is possible that the nutritional supplement that we provide may not taste good to you.

BENEFITS

There are some benefits to participating in this study. We will provide you with a clean delivery kit, iron tablets, medicine for worms, and a baby blanket. We will also provide tetanus vaccine to you if you need it and have not received it at your routine antenatal care visit. We ask that you take the medicine for worms only after you are in your second trimester of pregnancy. The iron tablets, deworming medicine, and tetanus vaccine will improve the health of you during your pregnancy and will also improve the health of your growing baby.

All pregnant women and babies will be checked by our NNIPS workers, and while they cannot provide medical care, they can identify when your baby might be sick and can give you advice on when and where to seek care. The government may provide this care for free or you may have to pay some for it. We strongly encourage you to go to the local health center for antenatal care as well and to deliver your baby at an official birthing center.

We will tell you your hemoglobin level the day we take the finger stick blood sample. This will help determine the strength of your blood. If your hemoglobin level is too low, we will encourage you to take your iron tablets and, if needed, refer you to your local health center.

Confidential: For Review Only

The information we learn in this study may be helpful for current and future babies in your community.

CONFIDENTIALITY

All information you provide will remain secure with NNIPS staff and will not be shared with anyone who is not working in this project or those organizations who monitor research studies to be sure they are being done properly. We will share your information with the funding agency and other partners for this project but none of that information will identify you personally. We will keep all the forms and data in locked cabinets at our NNIPS office and in protected computer files. The information you provide will only be used for this research study.

With your permission, we want to keep your information in our files in case there will be another NNIPS study in the future.

VOLUNTARY PARTICIPATION

You have a choice to be in this project and you may say yes or no. If you do not wish to be in this project, your other option is to follow the diet you want to, and have routine care given to women in this area.

If you do agree to participate, you can also stop or withdraw from the study at any time. You will also be able to withdraw your permission for us to share your information with the funding agency and other partners for this project. This will not affect your access to health care or NNIPS activities now or in the future. All things provided to you in this study are free, but you will not be otherwise compensated for your participation.

PERSON TO CONTACT

If you have any questions or problems about the study, I can answer them now, or you can contact the Project Director, Dr Subarna Khatry at NNIPS office in Hariaun, Sarlahi (phone no. 046-530135). You can also contact the Nepal Health Research Council in Kathmandu if you have any concerns (phone no. 1-4227460).

You can also contact these people if you believe you have been injured by being in this study.

Do you give permission to participate in this study? Check: Yes _____ No _____

Do you give permission to share your information with the funding agency and other partners?

Check: Yes _______ No _________

Do you give permission to be contacted for future studies? Check: Yes ___ No ___

PERMISSION TO PROCEED

Confidential: For Review Only

Woman Consenting:

Name Signature Date

NNIPS Staff Witnessing Consent:

Name Signature Date

Husband's or Guardian's Consent for Women Younger than 18 yrs.:

Name Signature Date

Confidential: For Review Only

BMJ Paediatrics Open

Supplementation with fortified balanced energy-protein during pregnancy and lactation and its effects on birth outcomes and infant growth in southern Nepal: protocol of a 2x2 factorial randomized trial

Journal:	*BMJ Paediatrics Open*
Manuscript ID	bmjpo-2023-002229.R1
Article Type:	Protocol
Date Submitted by the Author:	22-Aug-2023

Complete List of Authors:	Erchick, Daniel; Johns Hopkins Bloomberg School of Public Health, Department of International Health<br>Lama, Tsering P.; Johns Hopkins Bloomberg School of Public Health, Department of International Health; Nepal Nutrition Intervention Project Sarlahi (NNIPS)<br>Khatry, Subarna K.; Nepal Nutrition Intervention Project Sarlahi (NNIPS)<br>Katz, Joanne; Johns Hopkins Bloomberg School of Public Health, Department of International Health<br>Mullany, Luke C.; Johns Hopkins Bloomberg School of Public Health, Department of International Health<br>Zavala, Eleonor; Johns Hopkins Bloomberg School of Public Health, Department of International Health<br>LeClerq, Steven C.; Johns Hopkins Bloomberg School of Public Health, Department of International Health; Nepal Nutrition Intervention Project Sarlahi (NNIPS)<br>Christian, Parul; Johns Hopkins Bloomberg School of Public Health, Department of International Health<br>Tielsch, James M.; George Washington University, Department of Global Health

Keywords:	Epidemiology, Infant

SCHOLARONE™
Manuscripts

I, the Submitting Author has the right to grant and does grant on behalf of all authors of the Work (as defined in the below author licence), an exclusive licence and/or a non-exclusive licence for contributions from authors who are: i) UK Crown employees; ii) where BMJ has agreed a CC-BY licence shall apply, and/or iii) in accordance with the terms applicable for US Federal Government officers or employees acting as part of their official duties; on a worldwide, perpetual, irrevocable, royalty-free basis to BMJ Publishing Group Ltd ("BMJ") its licensees and where the relevant Journal is co-owned by BMJ to the co-owners of the Journal, to publish the Work in this journal and any other BMJ products and to exploit all rights, as set out in our licence.

The Submitting Author accepts and understands that any supply made under these terms is made by BMJ to the Submitting Author unless you are acting as an employee on behalf of your employer or a postgraduate student of an affiliated institution which is paying any applicable article publishing charge ("APC") for Open Access articles. Where the Submitting Author wishes to make the Work available on an Open Access basis (and intends to pay the relevant APC), the terms of reuse of such Open Access shall be governed by a Creative Commons licence – details of these licences and which Creative Commons licence will apply to this Work are set out in our licence referred to above.

Other than as permitted in any relevant BMJ Author's Self Archiving Policies, I confirm this Work has not been accepted for publication elsewhere, is not being considered for publication elsewhere and does not duplicate material already published. I confirm all authors consent to publication of this Work and authorise the granting of this licence.

Confidential: For Review Only

Supplementation with fortified balanced energy-protein during pregnancy and lactation and its effects on birth outcomes and infant growth in southern Nepal: protocol of a 2x2 factorial randomized trial

Daniel J. Erchick[1], Tsering P. Lama[1,2], Subarna K. Khatry[2], Joanne Katz[1], Luke C. Mullany[1], Eleonor Zavala[1], Steven C. LeClerq[1,2], Parul Christian[1], James M. Tielsch[3]

[1] Department of International Health, Johns Hopkins Bloomberg School of Public Health, Baltimore, MD, USA

[2] Nepal Nutrition Intervention Project Sarlahi (NNIPS), Kathmandu, Nepal

[3] Department of Global Health, Milken Institute School of Public Health, George Washington University, Washington, District of Columbia, USA

Corresponding author: Daniel Erchick, Department of International Health, Johns Hopkins Bloomberg School of Public Health, Baltimore, MD, USA. derchick@jhu.edu

Confidential: For Review Only

Abstract

Introduction: Many women in low and middle-income countries (LMICs) enter pregnancy with low nutritional reserves with increased risk for fetal growth restriction (FGR) and poor birth outcomes, including small-for-gestational-age (SGA) and preterm birth. Balanced energy-protein (BEP) supplements have shown reductions in risk of stillbirth and SGA, yet variations in intervention format and composition and limited evidence on the impact of BEP during lactation on growth outcomes, warrant further study. This paper describes the protocol of the Maternal Infant Nutrition Trial (MINT) study, which aims to evaluate the impact of a fortified BEP supplement during pregnancy and lactation on birth outcomes and infant growth in rural Nepal.

Methods and analysis: MINT is a 2x2 factorial, household randomized, unmasked, efficacy trial conducted in a sub-area of Sarlahi District, Nepal. The study area covers six rural municipalities with about 27,000 households and a population of approximately 100,000. Married women (15-30 years) who become pregnant are eligible for participation in the trial and are randomly assigned at enrollment to supplementation with fortified BEP or not and at birth to fortified BEP supplementation or not until 6 months postpartum. The primary pregnancy outcome is incidence of SGA, using the INTERGROWTH-21st standard, among live born infants with birth weight measured within 72 hours of delivery. The primary infant growth outcome is mean length-for-age z-score (LAZ) at 6 months using the WHO international growth reference.

Ethics and dissemination: The study was approved by Institutional Review Board (IRB) at Johns Hopkins Bloomberg School of Public Health, Baltimore, USA, (IRB00009714), the Committee on Human Research IRB at The George Washington University, Washington, DC, USA (081739), and the Ethical Review Board of the Nepal Health Research Council, Kathmandu, Nepal (174/2018).

Trial registration number: NCT03668977

Key words: Balanced energy-protein, maternal nutrition, small-for-gestational-age, pregnancy outcomes, infant growth, randomized trial, Nepal

Protocol version: Version 6, July 20, 2023

Confidential: For Review Only

KEY MESSAGES

What is already known on this topic: The World Health Organization recommendations on antenatal care include balanced energy-protein (BEP) supplementation in undernourished populations to reduce the risk of stillbirth and SGA birth.

What this study hopes to add: This study will demonstrate whether fortified BEP reduces risk of adverse pregnancy outcomes beyond multiple micronutrient supplementation. MINT will also generate evidence whether maternal intervention prevents early life growth faltering and improves maternal outcomes, such as anemia and inadequate gestational weight gain.

How this study might affect research, practice or policy: The MINT trial will help refine and implement the WHO context-specific recommendation on BEP supplementation in pregnancy and provide evidence for potential intervention in pregnancy and postpartum on infant growth outcomes.

Confidential: For Review Only

INTRODUCTION

Many women in low and middle-income countries (LMICs) enter pregnancy with low nutritional reserves, unable to meet the increased requirements for protein, energy, and micronutrients during pregnancy and lactation. Undernourished women are at increased risk of fetal growth restriction (FGR) and adverse birth outcomes, including preterm birth (<37 gestational weeks) and small-for-gestational age (SGA) (birth weight <10th percentile of a reference standard by gestational age and sex), two underlying biologic causes of low birth weight (LBW) (<2.5 kg).[1] SGA infants are more likely to experience higher mortality, linear growth faltering and poor health outcomes, poor cognitive performance, and diminished educational and employment attainment.[2-4] In 2020, 23.4 million infants (17.4% of all births) were born SGA, with the highest prevalence in South Asia (40.9%).[5, 6] Estimates from 2012 indicate that 22% neonatal deaths in LMICs, and 26% in South Asia, were attributable to SGA.[7, 8]

Infant size status at birth and growth in the first 6-months of life, especially among preterm and SGA infants, are critical determinants of childhood stunting and wasting associated with higher risk for mortality, morbidity, and poor developmental outcomes.[9] Many maternal risk factors associated with pregnancy outcomes and consequent preterm or SGA are likely contributors to linear growth failure.[10] Postnatal factors, such as breastfeeding and feeding behaviors, infections, and environmental factors, are also important drivers of infant growth.[11, 12]

Nutritional interventions targeted to women during pregnancy and lactation provide an opportunity to break the intergenerational cycle of growth failure. The World Health Organization (WHO) recommendations on antenatal care (ANC) include balanced energy-protein (BEP) supplementation in undernourished populations to reduce the risk of stillbirth and SGA birth.[13] This is based on a systematic review of BEP supplementation during pregnancy that has shown decreased risk of stillbirth (relative risk (RR): 0.60, 95% confidence interval (CI): 0.39 to 0.94) and SGA (RR: 0.79, 95% CI: 0.69 to 0.90),.[14] BEP also increased mean infant birth weight by 41g overall and 100g among undernourished women.[14, 15]

Despite this context-specific recommendation, the variation in formats, ingredients, and nutrient composition have hindered programmatic progress. Further, there is no recommendation for supplementation of women who are breastfeeding, despite high rates of growth faltering in early life in LMICs, where maternal undernutrition is high and exclusive breastfeeding is recommended in the first 6 months of life. Recently, an expert consensus developed specifications for a fortified BEP "ready-to-use" food supplement for pregnant and lactating women in LMICs.[16] Such a product requires testing in undernourished settings for efficacy in improving birth outcomes and infant growth.

In this paper, we describe the study protocol for the Maternal Infant Nutrition Trial (MINT) at the Nepal Nutrition Intervention Project Sarlahi (NNIPS) research site in Sarlahi District, Nepal. The aim of the MINT study is to evaluate the efficacy of daily fortified BEP supplementation during pregnancy and lactation on SGA and mean length-for-age z-score (LAZ) in the first six months of life. In this rural population, there is a significant burden of undernutrition among mothers and infants and high prevalence of adverse pregnancy outcomes.[17-19] However, breastfeeding

Confidential: For Review Only

through 6 months of life is high, presenting postpartum supplementation as a plausible mechanism by which to improve infant growth. Our primary hypotheses are that 1) fortified BEP supplementation vs. no supplementation during pregnancy will reduce incidence of SGA, and 2) fortified BEP supplementation vs. no supplementation during lactation alone or both during pregnancy and lactation will improve infant linear growth in the first six months after delivery. Several secondary outcomes will also be examined.

Confidential: For Review Only

METHODS AND ANALYSIS

Study design

MINT is a 2x2 factorial, household randomized, unmasked, efficacy trial of fortified BEP supplementation vs. no supplementation among women during pregnancy and lactation on SGA and infant growth to 6 months after delivery in rural Nepal (Figure 1). This community-based trial began in November 2021, and will enroll approximately 1,800 mother-fetus/infant pairs over the course of about 20 months.

We nested several sub-studies within the trial to explore the impact of the intervention on maternal dietary intake during pregnancy and postpartum, infant neurodevelopment at 6 months after delivery, metabolites in blood during pregnancy, and the gut microbiota among mothers in pregnancy and infants in the postnatal period.

This protocol follows the Standard Protocol Items: Recommendations for Interventional Trials (SPIRIT) guidelines (Supplementary Material, Table 1).[20]

Study setting

The MINT study is conducted in Sarlahi District, Madhesh Province, in the Terai region (low lying plains) of Nepal by the NNIPS research team. The NNIPS field research site, established in 1989 and covering approximately one-third of the district, currently operates as a joint undertaking between the Department of International Health, Johns Hopkins Bloomberg School of Public Health (JHSPH), and the nonprofit organization, Nepal Netra Jyoti Sangh, under a memorandum of agreement with the Social Welfare Council, Government of Nepal. Nepal's Terai region has high rates of maternal undernutrition, neonatal mortality, adverse pregnancy outcomes, and inadequate infant growth, and is representative of rural, low-income settings in much of South Asia.[21] Over half (56.9%) of households in Nepal's Madhesh Province faced food insecurity with 10% being severely food insecure, according to the Nepal Demographic and Health Survey 2016.[21] Of children under five years old, 29.3% were stunted and 10.1% were wasted in Madhesh Province in 2022.[22] Data from our most recent trial in the NNIPS study area, which had over 31,000 live births (2010-2017), reported neonatal mortality and stillbirth rates of approximately 32 per 1,000 live births and 29 per 1,000 births respectively (unpublished). In that trial, the proportion of adverse pregnancy outcomes were: preterm birth 14.9%, SGA 48.5%, and LBW 30.1% (unpublished). Nearly a third of women in their first trimester of pregnancy were underweight (29.5%) (body mass index (BMI) <18.5 kg/m^2) and the same proportion (29.5%) had a height less than 145 cm (unpublished). Over a third of infants (35%) are breastfed within an hour of birth and 95% within 24 hours in our trial population (unpublished).

Study population and recruitment

The study area includes 34 wards selected from six rural municipalities (Haripur, Ishwarpur, Kabilasi, Malangawa, Chakraghatta and Chandranagar) with a population of approximately 100,000 people. MINT enumerated and recruited participants through a household census and

Confidential: For Review Only

pregnancy surveillance system. Due to disruptions caused by the COVID-19 pandemic, the trial census and enrollment was divided into two periods.

In July 2019, we conducted a census to enumerate the households in the study area with potentially eligible women. The census activity identified about 27,000 total households, including roughly 13,000 households with at least one woman meeting the eligibility criteria for pregnancy surveillance. The first enrollment period spanned November 2021 to June 2022, enrolling 938 women.

Between the original census and the start of pregnancy enrollment (which was delayed due to the COVID-19 pandemic), many newly married women accumulated in the study area. In December 2022 and January 2023, we conducted a new household census update activity to add these newly married women, who would be eligible for enrollment in the trial, to our census and pregnancy surveillance roster. The census update activity added about 1,400 newly married women to the census roster for a total of around 14,400 women eligible for pregnancy surveillance and subsequent enrollment if they become pregnant. The second enrollment period began in January 2023 and will run until the sample size of 1,800 women is met.

Pregnancy surveillance is conducted by local, female data collectors hired on the project to make household visits every 5 weeks to ask potential participants if they had a period since the last visit and offer a urine-based pregnancy test if not. All married women (15-30 years) resident in the study area at the time of the census who are identified as pregnant by our pregnancy surveillance system (<24 weeks gestation) and consent to participate are eligible for participation in the trial. Women who self-report an allergy to nuts, milk, or soy at enrollment are excluded.

We have enrolled 1,944 women as of August 2023.

Randomization/allocation

The study uses a household-randomization scheme as there are joint families in which more than one woman could contribute a pregnancy, which would increase risk of sharing/crossover in the context of individual randomization. At enrollment, randomization is stratified by maternal height (<150 cm, ≥150 cm). Using the median of three height measures of the first woman enrolled in the house, we assign each household to either supplementation or not during pregnancy within each height category. We assign any additional women who became pregnant in a household that had already been randomized to the same group. At the birth visit, we randomize within each height category and pregnancy allocation group, assigning each household to either supplementation or not during the postpartum period. Only households with at least one live birth are eligible for postpartum randomization and follow-up. Since the likelihood of more than one woman from the same household becoming pregnant within the enrollment period is estimated to be low, at ~96.5% from prior data , this choice of randomization approach allows us to gain the statistical strength of an individually randomized trial.

Confidential: For Review Only

We pre-generated randomization sequences using the R software environment for statistical programming (www.r-project.org). We generated these sequences using randomly permuted blocks of size 4, 6, 8, and 12, to ensure sequential (i.e., chronological) balance across the groups throughout the enrollment period.

Masking of participants or study data collectors was not feasible due to the nature of the intervention and control.

Intervention

At enrollment, households are randomly allocated to either supplementation or not for the duration of pregnancy. Women in the supplementation group receive at home a supply of fortified BEP supplement for daily consumption beginning at 14 gestational weeks until the pregnancy outcome. Both groups also receive i) recommendation to enroll in antenatal care (ANC) at a local health clinic and deliver at a certified birthing facility, ii) nutrition, hygiene, and breast feeding and infant care counseling, and iii) a clean birthing kit. In pregnancy, women in both groups are provided iron-folic acid tablets and albendazole, if not provided via antenatal care at their health facility. At the birth visit, households where a woman reports at least one live birth are randomly allocated to either supplementation or not for the postpartum period until the infant is 6 months old. Counseling for exclusive breast feeding is provided in the postpartum period but iron-folic acid tablets are not provided as per the policy in Nepal.

Composition of fortified BEP supplement

The fortified BEP supplement is a ready-to-eat snack in the form of lipid-based peanut paste packaged in individual sachets (72g). The supplement was designed to meet the expert consensus specifications of a nutritious food supplement for pregnant and lactating women.[16] Each sachet is a daily portion that provides calories (~400 kcal), protein (~14 g), and multiple micronutrients at the estimated average requirement for pregnancy. The product is made by Nutriset, France, as a specially-manufactured version of their product known as “Plumpy’Mum”. We previously conducted two formative research studies to evaluate the acceptability of and adherence to various flavors of the BEP product in this community.[23, 24] Due to unavailability of raw materials, Nutriset produced three variations of Plumpy’Mum for use in our study, each with different combinations of ingredients selected to produce a product with a similar nutritional composition. The changes to the original ingredients were to remove the soy isolate (first change) and soy flour (second change), which resulted in minor changes in energy, protein, and micronutrient concentrations, deemed inconsequential from a nutrition perspective. The specific ingredients and nutritional composition of each variation are provided in the Supplementary Material, Table 2.

Outcomes

The study has two primary outcomes, one each for the pregnancy and postpartum/postnatal periods, respectively. The primary outcome for the pregnancy period is incidence of SGA among live born infants with birth weight measured within 72 hours of delivery. SGA is defined as weight measured within <72 h of delivery <10th centile of the INTERGROWTH 21st reference standard by gestational age and sex.[25] We assessed gestational age using 1st or

Confidential: For Review Only

2nd trimester ultrasound examination prior to 24 gestational weeks. The primary outcome for the postpartum/postnatal period is mean LAZ at 6 months using the international WHO child growth reference.[26] Secondary outcomes are listed in Table 1.

Sample size

With a sample size of 1,548 woman/infant pairs visited within <72 hours of delivery, we will be able to detect a 17.5% reduction in SGA with 80% power and a 20.2% reduction with 90% power assuming a type 1 error of 5% (two-sided). Since we are assuming no interaction between the marginal effects, this sample size will allow us to detect a difference in infant LAZ at 6 months after delivery of 0.16 Z scores with 80% power and 0.18 Z scores with 90% power. The MINT trial is designed to enroll approximately 1,800-2,000 women over a period of 20 months (split into two 6-month enrollment periods). This will yield the required sample of 1,548 woman/infant pairs visited within 72 hours of delivery, after a reduction of 14% due to fetal death and loss to follow-up in pregnancy.

Data collection

All study visits and interviews are conducted in participant households because of the wide dispersion of households across this rural community and the impracticality of bringing participants to a central location (Figure 2). Data collection teams comprising Field Interviewers (FIs) schedule the study visits, administer the questionnaires, and conduct the assessments as described below.

Enrollment visit: FIs conduct the enrollment visit, after notification of a newly identified pregnancy, to administer informed consent, baseline questionnaires, and anthropometry. Questionnaires cover maternal demographic characteristics, socioeconomic status, pregnancy and reproductive history, dietary diversity, and household food security (Household Food Insecurity Access Scale (HFIAS) developed by the Food and Nutrition Technical Assistance).[27] Anthropometric measurements include height (portable stadiometer), weight (MicroLife WS50 scale), mid-upper arm circumference (MUAC) (non-stretch insertion tape), blood pressure, and pulse.

Ultrasound visit: A special cadre of Auxiliary Nurse Midwives (ANMs) conduct the ultrasound assessment for gestational dating by visiting the participant's homes (Supplementary Material, Section A). ANMs are trained to use a portable machine (SonoSite NanoMaxx) and follow the INTERGROWTH 21st methodology for early and late fetal gestational age assessment, including measurement of crown-rump length and/or biparietal diameter (outer-to-outer), head circumference, and femur length.[28, 29]

Weekly morbidity and supplement compliance visits: Data collectors visit each woman weekly during pregnancy starting at 14 weeks based on LMP and in the postpartum period until 6 months after delivery to provide the supplement, collect data on maternal morbidities, and assess supplement compliance.

Confidential: For Review Only

Late pregnancy visit: FIs conduct a late pregnancy visit between 32-34 gestational weeks to collect data on maternal anthropometry (same measures as enrollment), maternal morbidities, tobacco/alcohol use, ANC utilization, delivery planning, diet diversity, household food security, and depression (using a version of the Edinburgh Postnatal Depression Scale validated in Nepali).[30]

Birth assessment visit: FIs aim to visit each woman within 72 hours of delivery to collection information about the birth and delivery process, early breastfeeding and feeding behaviors, and newborn care practices. Infant weight (g), length (cm), head circumference (cm), and temperature (F) are measured. Weight is measured with digital scales (Tanita BD- 585), length with Shorr-type boards, and MUAC with non-stretch insertion tape. Non-digital measures are taken in triplicate and the median is used for analysis.

Postpartum/Postnatal monthly visits: After the birth visit, field interviewers conduct monthly postnatal visits up to 6 months after delivery to administer questionnaires on maternal and infant morbidity, breastfeeding and infant and young child feeding practices, infant care practices, household food security, and anthropometric measurements for the infant (weight, length, head circumference, MUAC) and mother (weight, MUAC). Additionally, we will visit as many woman/infant pairs at 12 months after delivery as possible until the administrative end of the study to allow us to assess infant growth at 12 months as a secondary aim in a sub-sample.

Biospecimen sub-studies: The MINT study will enroll two sub-studies for biospecimen collection (Supplementary Material, Table 3). First, we will enroll about 120 pregnant women during the first enrollment period from the pregnancy supplementation (n=60) and not groups (n=60). At the enrollment and late pregnancy visits, these women will provide volumetric absorptive microsampling (VAM) samples, for serum metabolomics analyses collected via finger-stick using Neoteryx kits, hemoglobin measurement, a maternal stool sample stored in nucleic acid buffer solution (DNA Genotek OMR-GUT), and their infants will provide a stool sample at 6 months. Second, we will enroll about 120 pregnant women during the second enrollment period from the pregnancy supplementation (n=60) and not groups (n=60), and these women will provide a hemoglobin measurement at the enrollment and late pregnancy visits, and their infants will provide a stool sample at 3 and 6 months.

Dietary intake: Field interviewers will conduct duplicate 24-hour dietary recalls at the late pregnancy and 3-months postpartum visits for a subset of 240 women in the first (n=120) and second (n=120) enrollment periods. The purpose of these data is to assess nutrient intakes and determine whether dietary substitution is occurring in the supplement group. Interviewers use the multiple pass method to help the woman recall all the components of her diet from the previous day. A local food list, photo atlas, and electronic entry form have been adapted from previous work in Nepal to assist with portion size recall and data entry.[31, 32] Daily nutrient intakes will be estimated with local recipes and national food composition tables from India and Nepal.

Confidential: For Review Only

Bayley Scales of Infant and Toddler development (BSID-4) visit: A specialized team with experience and training in developmental assessment in children will visit a sub-sample of 400 mother/infant pairs to evaluate infant neurodevelopment at 6 months of life.[33, 34] The visit will include the BSID-4 assessment, a standardized evaluation for infants that measures functioning in five areas: cognition, fine motor, gross motor, receptive language, and expressive language. At this visit, data collectors will also conduct the World Health Organization six gross motor milestones module.[35]

Data management

Our field interviewers administer study questionnaires and record anthropometric measurements via digital tablets with a cellular connection using a customized web-based data management system developed on the Microsoft ASP.NET framework. The local data collectors complete paper forms that are recorded by data entry operators on a weekly basis. On the backend, data are stored in a relational database management system (Microsoft SQL Server). Prior to study launch, the field team tests all forms, and restrictions and checks are programmed to reduce data entry errors. The senior field team and co-investigators review important variables weekly to monitor data quality issues, such as missingness, heaping, digit preference, and outliers. The senior field team corrects data entry errors and implements retraining of data collectors on specific protocols when necessary.

Data monitoring

Data and safety monitoring board: An independent Data and Safety Monitoring Board (DSMB) is established for the trial to advise the investigators on issues of safety and efficacy. DSMB membership includes four individuals from Nepal, including a pediatrician, obstetrician-gynecologist, nutritionist, and statistician. The DSMB met prior to the start of the study, twice during implementation of the study, and will meet at least once more.

Serious adverse events: Serious adverse events, as defined under Good Clinical Practice (GCP) guidelines, include death, a life-threatening reaction to the supplement or other study procedure, hospitalization for a reason other than routine delivery, significant or persistent disability or impairment, or a congenital anomaly/birth defect. All data collection staff are trained to recognize a range of serious and other adverse events (AEs). SAEs are investigated and reported to the DSMB and IRBs according to the study research and ethical protocols. Our most senior data collectors conduct verbal autopsies in the case of a maternal or infant death.

Statistical analysis

Primary aim 1: We will undertake an intention-to-treat analysis to examine the effect of supplementation on the first primary outcome of SGA. The analysis will include all live births for whom a birth weight was collected by study data collectors within 72 hours of delivery for the SGA (<10th centile births using the INTERGROWTH-21st standard) outcome. We will assess the balance between the two pregnancy supplementation groups in baseline household- and individual-level characteristics including maternal age, height, body mass index (BMI), parity, previous adverse pregnancy outcome, education, and socioeconomic status. If imbalanced variables are also associated with the primary outcome ($p<0.10$), we will use multivariable log

Confidential: For Review Only

binomial regression modeling to adjust for potential confounding. We will compare the proportion of SGA, between those who were supplemented vs. not according to their allocation status, using a log binomial regression model to estimate the relative risk and 95% confidence interval with the control group as the referent both unadjusted and adjusted for identified confounders. If the final model does not converge, we will use Poisson regression with robust standard error estimation.

Primary aim 2: We will undertake an intention-to-treat analysis to examine the effect of supplementation on the second primary outcome of mean LAZ at 6 months. The analysis will include all children whose length was assessed at 6 months of age. We will assess the balance across the four pregnancy and lactation supplementation groups in baseline household- and individual-level characteristics. If imbalanced variables are also associated with the secondary outcome ($p<0.10$), we will use multivariable linear regression modeling to adjust for potential confounding. Due to the factorial study design, we will assess the interaction between pregnancy and lactation supplementation on the outcome of mean LAZ, although our sample size is not adequate to detect small interaction effects. We will use a multivariable linear regression model with an interaction term for treatment arms. If the interaction is significant (<0.10), we will report both the main and interaction effects. If not significant, which we anticipate, we will report the marginal treatment effects for supplementation during lactation vs. not and during pregnancy vs. not. We will compare mean LAZ scores between supplementation groups using a multivariable linear regression model to estimate the risk difference and 95% confidence interval with the control group as the referent both unadjusted and adjusted for identified confounders.

ETHICS AND DISSEMINATION

Ethical approval

The study was approved by Institutional Review Board (IRB) at Johns Hopkins Bloomberg School of Public Health, Baltimore, USA, (IRB00009714), the Committee on Human Research IRB at The George Washington University, Washington, DC, USA (081739), and the Ethical Review Board of the Nepal Health Research Council, Kathmandu, Nepal (174/2018).

Patient and public involvement

We conducted two formative studies to assess compliance with and acceptability of various formulations of BEP supplements. Our field leadership team also engaged several local stakeholders prior to the start of the study, including municipality mayors, health coordinators, and health facility in-charges in the study area. After completion of the study, the team will conduct similar meetings to disseminate the results.

Ancillary care

The MINT study provides iron folic acid tablets and albendazole to women who report that they have not received these from the health facility during their ANC visits. Women with low hemoglobin (Hb <11g/dL) are referred to a health facility. Women with suspected fetal

Confidential: For Review Only

abnormality detected during the ultrasound gestational age dating visit are referred for clinical examination.

Confidentiality

Study investigators and staff treat all data as confidential, and participants are informed that their information will be protected. All data collection forms are kept in secure locations with access limited to relevant study staff. All electronic databases are stored on encrypted, password-protected servers, and data transfer uses secure encrypted transfer protocols. Access to identifying information is restricted to the principal investigator and few key co-investigators and staff. Only anonymized datasets are shared beyond the study team.

Dissemination plan

The study team will disseminate MINT findings to the Sarlahi District Public Health Office, Sarahi District Project Advisory Committee, Ministry of Health and Population, Nepal, Central Project Advisory Committee, and to various local organizations, including Unicef-Nepal and Unicef South Asian Regional Office, USAID, and other stakeholders in Nepal with interests in nutrition during pregnancy and lactation. We will share anonymized data with the Ki data repository and researchers involved in the BEP Harmonization Initiative organized and funded by the BMGF for the purpose of meta-analyses using data from similar trials conducted around the world. The study team will also present results at international workshops and conferences with relevant researchers and program or policy stakeholders. Members of the study team will publish results of primary and secondary analyses in peer-reviewed academic journals.

Confidential: For Review Only

DISCUSSION

Nepal has made progress in reducing maternal, newborn, and child mortality, but a significant burden of undernutrition remains among mothers and infants.[21, 22] Novel and effective interventions and practical implementation strategies are needed to prevent adverse pregnancy outcomes and infant growth faltering,[36, 37] and currently WHO (2016)[13] recommends use of BEP supplements in undernourished settings, but standard formulations of such interventions and data on implementation in programs is lacking. The MINT trial will provide evidence to help refine and implement the WHO context-specific recommendation on BEP supplementation in pregnancy, especially using the fortified, enhanced ready-to-use formulation recommended by the expert consensus on BEP supplements.[16] As multiple micronutrient supplementation (MMS) reduces risk of adverse pregnancy outcomes, including low birth weight and SGA, our trial will contribute evidence as to whether the fortified BEP intervention, which include the same 15 micronutrients, provides additional benefit beyond MMS alone.[38] This trial will generate evidence on the potential of a maternal intervention to prevent early life growth faltering and improve maternal outcomes, such as anemia and inadequate gestational weight gain, areas where a research gap exists. The results from the trial will inform health policy and programs in Nepal, where there currently are no guidelines for community-level management of growth faltering in infants less than 6 months of age beyond exclusive breastfeeding. The primary strength of the trial is the 2x2 factorial RCT design, which will enable understanding of the interconnections between maternal nutritional status in pregnancy and lactation and relationships to fetal and infant growth faltering. The trial includes frequent follow-up visits, over the continuum from early pregnancy to 6 months after delivery, which will generate data on many prenatal and postpartum factors that influence maternal and infant outcomes. The trial will describe maternal dietary intake, assess whether provision of BEP supplements lead to dietary substitution, and measure adherence, yielding practical insights for implementation. Lastly, the trial structure allows for nesting various sub-studies, including those to assess the impact of BEP on the maternal and infant microbiome, infant neurodevelopment, and maternal metabolites.

Confidential: For Review Only

Acknowledgements
We express our sincere thanks to the women and their families in Sarlahi, District, Nepal, who participated in the MINT study. Thank you to Nutriset, France, for producing and donating a portion of the fortified BEP supplements. This study was conducted in collaboration with our implementing partner, Nepal Netra Jyoti Sangh, under the auspices of the Social Welfare Council of the Government of Nepal.

Competing interests
The authors declare no competing interests.

Authors' contributions
JT, JK, LM, and SKK conceptualized the study. All authors contributed to development of the study design, protocols, and forms and tools. SKK, TPL, and SCL led data collection and supervision in the field. DE wrote the first draft of the manuscript. All authors provided revisions and inputs to the manuscript. All authors reviewed and agreed on the final version of the manuscript.

Data availability
The principal investigator will share final anonymized data with the Ki data repository and researchers involved in the BEP Harmonization Initiative for the purpose of meta-analyses using data from similar trials conducted around the world. Data will also be available to other investigators upon reasonable request.

Funding
This study was supported by the Bill & Melinda Gates Foundation (OPP11599195), Thrasher Research Fund (TRF01278), and the Eunice Kennedy Shriver National Institute of Child Health and Human Development (R01HD109385). The funders had no role in the design, implementation, or analysis of the trial.

Confidential: For Review Only

Table 1: MINT study primary and secondary outcomes

**Primary outcomes**	
Small-for-gestational age (10th centile)	Proportion of infants with birth weight <10th centile of INTERGROWTH 21st reference standard by gestational age and sex for infants measured <72 hours after delivery.
Length-for-age Z (LAZ)	Mean infant length-for-age Z score at 6 months of age using the World Health Organization Child Growth Study criteria.
**Secondary outcomes**	
**Newborn**	
Small-for-gestational-age (3rd centile)	Proportion of infants with birth weight <3rd centile of INTERGROWTH 21st reference standard by gestational age and sex for infants measured <72 hours after delivery.
Large-for-gestational-age (90th centile)	Proportion of infants with birth weight >90th centile of INTERGROWTH 21st reference standard by gestational age and sex for infants measured <72 hours after delivery.
Short-for-gestational-age (10th centile)	Proportion of infants with birth length <10th centile of INTERGROWTH 21st reference standard by gestational age and sex for infants measured <72 hours after delivery.
Short-for-gestational-age (3rd centile)	Proportion of infants with birth length <3rd centile of INTERGROWTH 21st reference standard by gestational age and sex for infants measured <72 hours after delivery.
Birth weight	Mean infant birth weight.
Birth length	Mean infant birth length.
Head circumference	Mean infant head circumference
Arm circumference	Mean infant mid-upper arm circumference
Low birth weight	Proportion of infants with birth weight <2,500g for infants measured <72 hours after delivery.
Gestational age	Mean infant gestational age at delivery.
Preterm birth (<37 weeks)	Proportion of pregnancies that are <37 gestational weeks using the INTERGROWTH 21st standards for 1st and 2nd trimester ultrasound dating.
Very, extremely preterm (<34, <32, <28 weeks)	Proportion of pregnancies that are <34, <32, <28 gestational weeks using the INTERGROWTH 21st standards for 1st and 2nd trimester ultrasound dating.
Stillbirth	Proportion of pregnancies resulting in stillbirth, defined as fetal death ≥28 gestational weeks, using the INTERGROWTH 21st standards for 1st and 2nd trimester ultrasound dating.

Weight-for-length Z score at 6 months of age	Mean infant weight-for-length Z score at 6 months of age using the World Health Organization Child Growth Study criteria.
Weight-for-age Z score at 6 months of age	Mean infant weight-for-age Z score at 6 months of age using the World Health Organization Child Growth Study criteria.
Stunting	Proportion of infants with <-2 length-for-age Z score at 6 months of age using the World Health Organization Child Growth Study criteria.
Wasting	Proportion of infants with <-2 weight-for-length Z score at 6 months of age using the World Health Organization Child Growth Study criteria.
Underweight	Proportion of infants with <-2 weight-for-age Z score at 6 months of age using the World Health Organization Child Growth Study criteria.
Developmental scales	Bayley Scales of Infant and Toddler Development 4 (BSID-4) assessment at 6 months.
Gross motor milestones	World Health Organization 6 gross motor milestones at 6 months.
Gut microbiome	Gut microbiome diversity and stool metabolomics at 3, 6 months.
**Maternal**	
Gestational weight gain	Mean maternal weight gain from enrollment to late pregnancy visits.
Net gestational weight gain	Mean maternal weight gain from enrollment to maternal birth assessment visit (after delivery).
Inadequate maternal gestational weight gain	Proportion of women with inadequate rate of gestational weight gain from enrollment to late pregnancy visit using the INTERGROWTH 21st reference for normal body mass index (BMI) and IOM reference for low and high BMI.
Inadequate maternal net gestational weight gain	Proportion of women with inadequate rate of gestational weight gain from enrollment to maternal birth assessment visit using the INTERGROWTH 21st reference for normal body mass index (BMI) and IOM reference for low and high BMI.
Mid-upper arm circumference	Mean change in mid-upper arm circumference from enrollment to late pregnancy visits.
Anemia	Proportion of women who are anemic (Hb <110 g/L) in late pregnancy.
Weight at 6 months postpartum	Mean maternal weight at 6 months postpartum.
Maternal dietary intake	Mean maternal dietary intake score in late pregnancy and 3 months postpartum.
Minimum maternal dietary intake	Proportion of women meeting the minimum dietary intake score in late pregnancy and 3 months postpartum.

Confidential: For Review Only

Gut microbiome	Gut microbiome diversity and stool metabolomics during pregnancy.
Metabolites	Metabolites in the blood during pregnancy.

Confidential: For Review Only

References

1. Kozuki N, Katz J, Lee AC, et al. Short Maternal Stature Increases Risk of Small-for-Gestational-Age and Preterm Births in Low- and Middle-Income Countries: Individual Participant Data Meta-Analysis and Population Attributable Fraction. J Nutr. 2015;145(11):2542-50.
2. Christian P, Lee SE, Donahue Angel M, et al. Risk of childhood undernutrition related to small-for-gestational age and preterm birth in low- and middle-income countries. Int J Epidemiol. 2013;42(5):1340-55.
3. Murray E, Fernandes M, Fazel M, et al. Differential effect of intrauterine growth restriction on childhood neurodevelopment: a systematic review. Bjog. 2015;122(8):1062-72.
4. Victora CG, Adair L, Fall C, et al. Maternal and child undernutrition: consequences for adult health and human capital. Lancet. 2008;371(9609):340-57.
5. Lawn J, Ohuma E, Bradley E, et al. Small babies, big risks: Global estimates of prevalence and mortality for vulnerable newborns to accelerate change and improve counting. Lancet. 2023.
6. Erchick DJ, Hazel EA, Katz J, et al. Vulnerable newborn types: analysis of subnational, population-based birth cohorts for 541 285 live births in 23 countries, 2000-2021. Bjog. 2023.
7. Katz J, Lee AC, Kozuki N, et al. Mortality risk in preterm and small-for-gestational-age infants in low-income and middle-income countries: a pooled country analysis. Lancet. 2013;382(9890):417-25.
8. Lee AC, Kozuki N, Cousens S, et al. Estimates of burden and consequences of infants born small for gestational age in low and middle income countries with INTERGROWTH-21(st) standard: analysis of CHERG datasets. Bmj. 2017;358:j3677.
9. Benjamin-Chung J, Mertens A, Colford JM, et al. Early childhood linear growth failure in low- and middle-income countries. medRxiv. 2020:2020.06.09.20127001.
10. Christian P. Prenatal origins of malnutrition. Emerging Societies Coexistence of Childhood Malnutrition and Obesity. 632009.
11. Black RE, Victora CG, Walker SP, et al. Maternal and child undernutrition and overweight in low-income and middle-income countries. Lancet. 2013;382(9890):427-51.
12. Keusch GT, Rosenberg IH, Denno DM, et al. Implications of acquired environmental enteric dysfunction for growth and stunting in infants and children living in low- and middle-income countries. Food Nutr Bull. 2013;34(3):357-64.
13. World Health Organization. WHO Guidelines Approved by the Guidelines Review Committee. WHO Recommendations on Antenatal Care for a Positive Pregnancy Experience. Geneva: World Health Organization; 2016.
14. Ota E, Hori H, Mori R, et al. Antenatal dietary education and supplementation to increase energy and protein intake. Cochrane Database Syst Rev. 2015(6):Cd000032.
15. Imdad A, Bhutta ZA. Maternal nutrition and birth outcomes: effect of balanced protein-energy supplementation. Paediatr Perinat Epidemiol. 2012;26 Suppl 1:178-90.
16. Members of an Expert Consultation on Nutritious Food Supplements for Pregnant and Lactating Women. Framework and specifications for the nutritional composition of a food supplement for pregnant and lactating women (PLW) in undernourished and low income settings. . Gates Open Res. 2019;3:1498 (document)
17. Subedi S, Hazel EA, Mohan D, et al. Prevalence and predictors of spontaneous preterm births in Nepal: findings from a prospective, population-based pregnancy cohort in rural Nepal-a secondary data analysis. BMJ Open. 2022;12(12):e066934.
18. Bryce E, Gurung S, Tong H, et al. Population attributable fractions for risk factors for spontaneous preterm births in 81 low- and middle-income countries: A systematic analysis. J Glob Health. 2022;12:04013.

Confidential: For Review Only

19. Hazel EA, Mohan D, Zeger S, et al. Demographic, socio-economic, obstetric, and behavioral factors associated with small-and large-for-gestational-age from a prospective, population-based pregnancy cohort in rural Nepal: a secondary data analysis. BMC Pregnancy Childbirth. 2022;22(1):652.
20. Chan AW, Tetzlaff JM, Altman DG, et al. SPIRIT 2013 statement: defining standard protocol items for clinical trials. Ann Intern Med. 2013;158(3):200-7.
21. Ministry of Health - MOH/Nepal, New ERA/Nepal, ICF. Nepal Demographic and Health Survey 2016. Kathmandu, Nepal: MOH/Nepal, New ERA, and ICF; 2017.
22. Ministry of Health - MOH/Nepal, New ERA/Nepal, ICF. Nepal Demographic and Health Survey Key Indicators Report 2022. Kathmandu, Nepal: MOH/Nepal, NEW ERA, and ICF; 2022.
23. Lama TP, Khatry SK, Isanaka S, et al. Acceptability of 11 fortified balanced energy-protein supplements for pregnant women in Nepal. Matern Child Nutr. 2022;18(3):e13336.
24. Lama TP, Moore K, Isanaka S, et al. Compliance with and acceptability of two fortified balanced energy protein supplements among pregnant women in rural Nepal. Matern Child Nutr. 2022;18(2):e13306.
25. Villar J, Cheikh Ismail L, Victora CG, et al. International standards for newborn weight, length, and head circumference by gestational age and sex: the Newborn Cross-Sectional Study of the INTERGROWTH-21st Project. Lancet. 2014;384(9946):857-68.
26. WHO Child Growth Standards based on length/height, weight and age. Acta Paediatr Suppl. 2006;450:76-85.
27. Coates J, Swindale, A, Bilinsky P. Household Food Insecurity Access Scale (HFIAS) for Measurement of Household Food Access: Indicator Guide (v. 3). Washington, D.C.: FHI 360/FANTA; 2007.
28. Papageorghiou AT, Kennedy SH, Salomon LJ, et al. International standards for early fetal size and pregnancy dating based on ultrasound measurement of crown–rump length in the first trimester of pregnancy. Ultrasound in Obstetrics & Gynecology. 2014;44(6):641-8.
29. Papageorghiou AT, Kemp B, Stones W, et al. Ultrasound-based gestational-age estimation in late pregnancy. Ultrasound Obstet Gynecol. 2016;48(6):719-26.
30. Bhusal BR, Bhandari N, Chapagai M, et al. Validating the Edinburgh Postnatal Depression Scale as a screening tool for postpartum depression in Kathmandu, Nepal. Int J Ment Health Syst. 2016;10:71-.
31. Harris-Fry H, Paudel P, Karn M, et al. Development and validation of a photographic food atlas for portion size assessment in the southern plains of Nepal. Public Health Nutr. 2016;19(14):2495-507.
32. Harris-Fry H, Beard BJ, Harrisson T, et al. Smartphone tool to collect repeated 24 h dietary recall data in Nepal. Public Health Nutr. 2018;21(2):260-72.
33. Murray-Kolb LE, Rasmussen ZA, Scharf RJ, et al. The MAL-ED cohort study: methods and lessons learned when assessing early child development and caregiving mediators in infants and young children in 8 low- and middle-income countries. Clin Infect Dis. 2014;59 Suppl 4(Suppl 4):S261-72.
34. Ranjitkar S, Kvestad I, Strand TA, et al. Acceptability and Reliability of the Bayley Scales of Infant and Toddler Development-III Among Children in Bhaktapur, Nepal. Front Psychol. 2018;9:1265.
35. Wijnhoven TM, de Onis M, Onyango AW, et al. Assessment of gross motor development in the WHO Multicentre Growth Reference Study. Food Nutr Bull. 2004;25(1 Suppl):S37-45.
36. Bhutta ZA, Das JK, Rizvi A, et al. Evidence-based interventions for improvement of maternal and child nutrition: what can be done and at what cost? Lancet. 2013;382(9890):452-77.
37. Bhutta ZA, Das JK, Bahl R, et al. Can available interventions end preventable deaths in mothers, newborn babies, and stillbirths, and at what cost? Lancet. 2014;384(9940):347-70.

Confidential: For Review Only

38. Keats EC, Haider BA, Tam E, et al. Multiple-micronutrient supplementation for women during pregnancy. Cochrane Database Syst Rev. 2019;3(3):Cd004905.

Confidential: For Review Only

Figure 1: Study design of MINT 2x2 factorial randomized trial

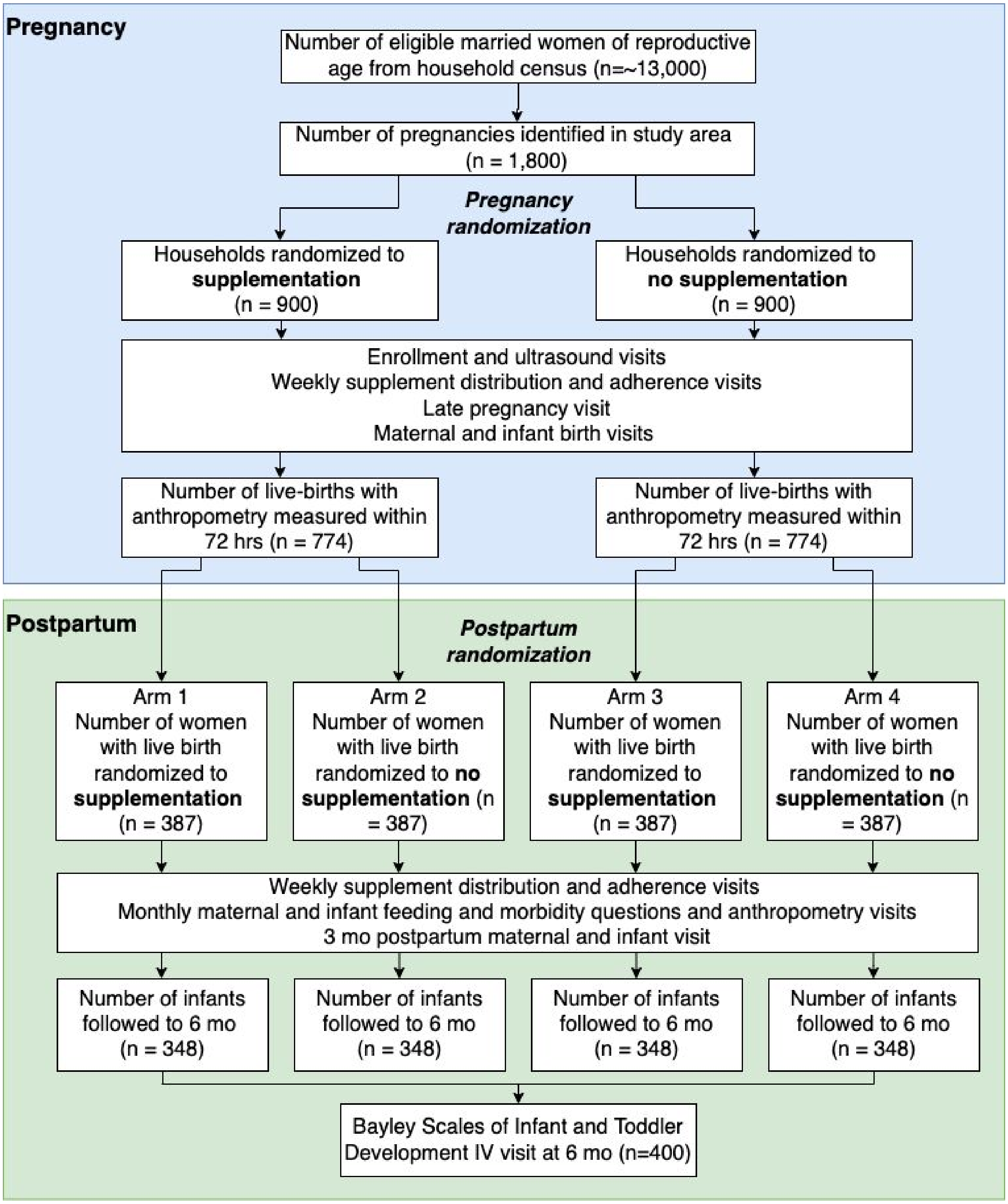

Figure 2: Study procedures flow diagram

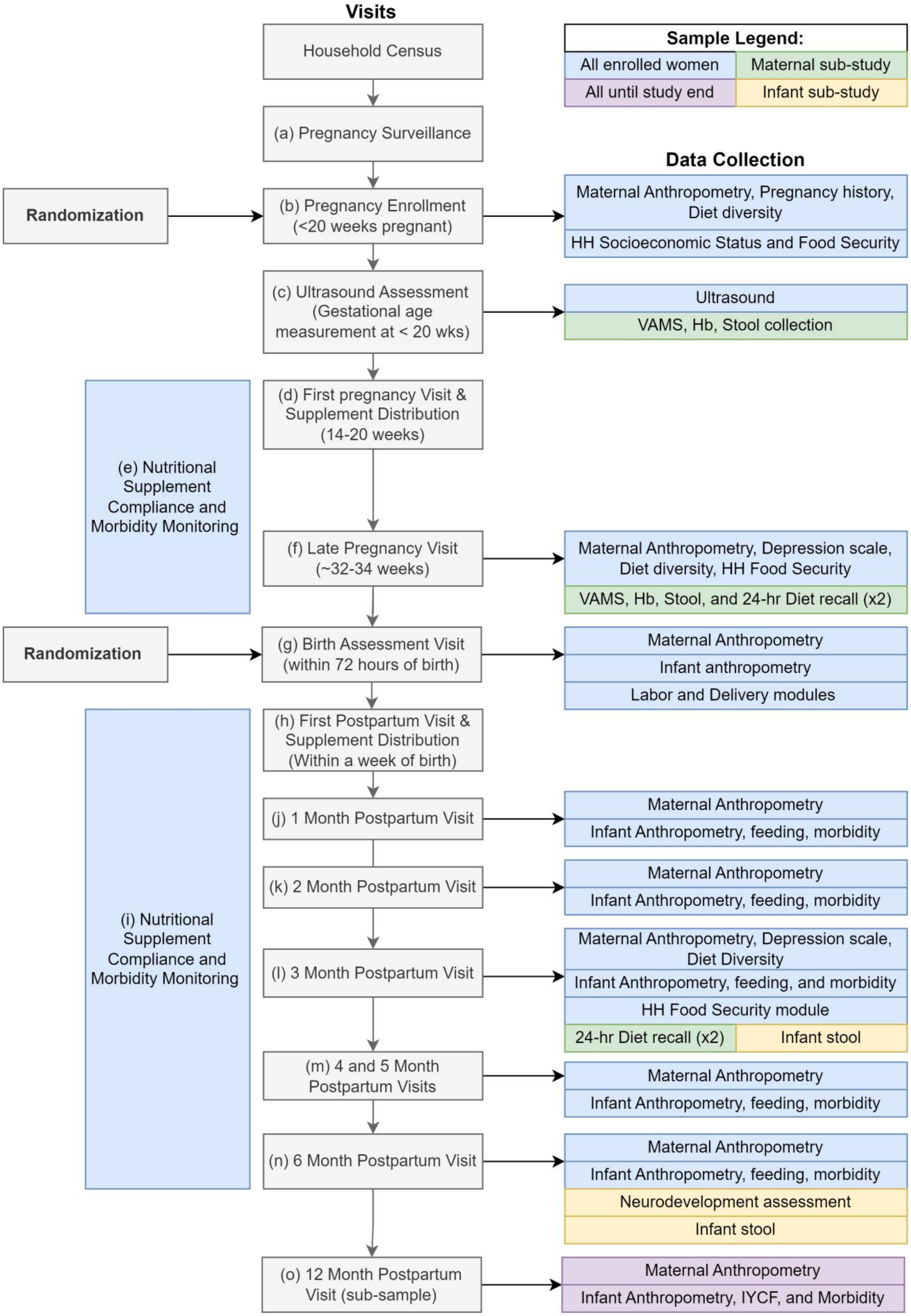

SUPPLEMENTARY MATERIAL

Table 1: Standard protocol items: recommendations for interventional trials (SPIRIT) guidelines checklist

SPIRIT 2013 Checklist: Recommended items to address in a clinical trial protocol and related documents*

**Section/item**	**Item No**	**Description**	**Addressed on page number**
**Administrative information**			
Title	1	Descriptive title identifying the study design, population, interventions, and, if applicable, trial acronym	_____1______
Trial registration	2a	Trial identifier and registry name. If not yet registered, name of intended registry	_____2______
	2b	All items from the World Health Organization Trial Registration Data Set	_____2______
Protocol version	3	Date and version identifier	_____2______
Funding	4	Sources and types of financial, material, and other support	____15______
Roles and responsibilities	5a	Names, affiliations, and roles of protocol contributors	_____1______
	5b	Name and contact information for the trial sponsor	_____15_____

Confidential: For Review Only

	5c	Role of study sponsor and funders, if any, in study design; collection, management, analysis, and interpretation of data; writing of the report; and the decision to submit the report for publication, including whether they will have ultimate authority over any of these activities	_____15______
	5d	Composition, roles, and responsibilities of the coordinating centre, steering committee, endpoint adjudication committee, data management team, and other individuals or groups overseeing the trial, if applicable (see Item 21a for data monitoring committee)	______NA_____
**Introduction**			
Background and rationale	6a	Description of research question and justification for undertaking the trial, including summary of relevant studies (published and unpublished) examining benefits and harms for each intervention	______4______
	6b	Explanation for choice of comparators	______4______
Objectives	7	Specific objectives or hypotheses	_____4-5______
Trial design	8	Description of trial design including type of trial (eg, parallel group, crossover, factorial, single group), allocation ratio, and framework (eg, superiority, equivalence, noninferiority, exploratory)	_____4-5______

Confidential: For Review Only

Methods: Participants, interventions, and outcomes

Study setting	9	Description of study settings (eg, community clinic, academic hospital) and list of countries where data will be collected. Reference to where list of study sites can be obtained	_____6______
Eligibility criteria	10	Inclusion and exclusion criteria for participants. If applicable, eligibility criteria for study centres and individuals who will perform the interventions (eg, surgeons, psychotherapists)	_____7______
Interventions	11a	Interventions for each group with sufficient detail to allow replication, including how and when they will be administered	_____8______
	11b	Criteria for discontinuing or modifying allocated interventions for a given trial participant (eg, drug dose change in response to harms, participant request, or improving/worsening disease)	_____8______
	11c	Strategies to improve adherence to intervention protocols, and any procedures for monitoring adherence (eg, drug tablet return, laboratory tests)	____9-10_____
	11d	Relevant concomitant care and interventions that are permitted or prohibited during the trial	_____8______

Outcomes	12	Primary, secondary, and other outcomes, including the specific measurement variable (eg, systolic blood pressure), analysis metric (eg, change from baseline, final value, time to event), method of aggregation (eg, median, proportion), and time point for each outcome. Explanation of the clinical relevance of chosen efficacy and harm outcomes is strongly recommended	_____8______
Participant timeline	13	Time schedule of enrolment, interventions (including any run-ins and washouts), assessments, and visits for participants. A schematic diagram is highly recommended (see Figure)	__9-10, 16____
Sample size	14	Estimated number of participants needed to achieve study objectives and how it was determined, including clinical and statistical assumptions supporting any sample size calculations	____8-9______
Recruitment	15	Strategies for achieving adequate participant enrolment to reach target sample size	____6-7______

Methods: Assignment of interventions (for controlled trials)

Allocation:

ly

Sequence generation	16a	Method of generating the allocation sequence (eg, computer-generated random numbers), and list of any factors for stratification. To reduce predictability of a random sequence, details of any planned restriction (eg, blocking) should be provided in a separate document that is unavailable to those who enrol participants or assign interventions	____7-8______
Allocation concealment mechanism	16b	Mechanism of implementing the allocation sequence (eg, central telephone; sequentially numbered, opaque, sealed envelopes), describing any steps to conceal the sequence until interventions are assigned	____7-8______
Implementation	16c	Who will generate the allocation sequence, who will enrol participants, and who will assign participants to interventions	____7-8______
Blinding (masking)	17a	Who will be blinded after assignment to interventions (eg, trial participants, care providers, outcome assessors, data analysts), and how	_____8______
	17b	If blinded, circumstances under which unblinding is permissible, and procedure for revealing a participant's allocated intervention during the trial	____NA______

Methods: Data collection, management, and analysis

Data collection methods	18a	Plans for assessment and collection of outcome, baseline, and other trial data, including any related processes to promote data quality (eg, duplicate measurements, training of assessors) and a description of study instruments (eg, questionnaires, laboratory tests) along with their reliability and validity, if known. Reference to where data collection forms can be found, if not in the protocol	___9-10_____
	18b	Plans to promote participant retention and complete follow-up, including list of any outcome data to be collected for participants who discontinue or deviate from intervention protocols	___9-10______
Data management	19	Plans for data entry, coding, security, and storage, including any related processes to promote data quality (eg, double data entry; range checks for data values). Reference to where details of data management procedures can be found, if not in the protocol	_____11_____
Statistical methods	20a	Statistical methods for analysing primary and secondary outcomes. Reference to where other details of the statistical analysis plan can be found, if not in the protocol	____11-12____
	20b	Methods for any additional analyses (eg, subgroup and adjusted analyses)	___11-12_____
	20c	Definition of analysis population relating to protocol non-adherence (eg, as randomised analysis), and any statistical methods to handle missing data (eg, multiple imputation)	___11-12_____

Methods: Monitoring

Data monitoring	21a	Composition of data monitoring committee (DMC); summary of its role and reporting structure; statement of whether it is independent from the sponsor and competing interests; and reference to where further details about its charter can be found, if not in the protocol. Alternatively, an explanation of why a DMC is not needed	____11______
	21b	Description of any interim analyses and stopping guidelines, including who will have access to these interim results and make the final decision to terminate the trial	____NA______
Harms	22	Plans for collecting, assessing, reporting, and managing solicited and spontaneously reported adverse events and other unintended effects of trial interventions or trial conduct	____11______
Auditing	23	Frequency and procedures for auditing trial conduct, if any, and whether the process will be independent from investigators and the sponsor	____NA______

Ethics and dissemination

Research ethics approval	24	Plans for seeking research ethics committee/institutional review board (REC/IRB) approval	____12______

w Only

Protocol amendments	25	Plans for communicating important protocol modifications (eg, changes to eligibility criteria, outcomes, analyses) to relevant parties (eg, investigators, REC/IRBs, trial participants, trial registries, journals, regulators)	____12______
Consent or assent	26a	Who will obtain informed consent or assent from potential trial participants or authorised surrogates, and how (see Item 32)	_____9______
	26b	Additional consent provisions for collection and use of participant data and biological specimens in ancillary studies, if applicable	_____NA_____
Confidentiality	27	How personal information about potential and enrolled participants will be collected, shared, and maintained in order to protect confidentiality before, during, and after the trial	___12-13_____
Declaration of interests	28	Financial and other competing interests for principal investigators for the overall trial and each study site	_____15_____
Access to data	29	Statement of who will have access to the final trial dataset, and disclosure of contractual agreements that limit such access for investigators	_____15_____
Ancillary and post-trial care	30	Provisions, if any, for ancillary and post-trial care, and for compensation to those who suffer harm from trial participation	_____NA_____

Confidential: For Revi

Dissemination policy	31a	Plans for investigators and sponsor to communicate trial results to participants, healthcare professionals, the public, and other relevant groups (eg, via publication, reporting in results databases, or other data sharing arrangements), including any publication restrictions	______13_____
	31b	Authorship eligibility guidelines and any intended use of professional writers	______15_____
	31c	Plans, if any, for granting public access to the full protocol, participant-level dataset, and statistical code	______15_____
**Appendices**			
Informed consent materials	32	Model consent form and other related documentation given to participants and authorised surrogates	__Section C___
Biological specimens	33	Plans for collection, laboratory evaluation, and storage of biological specimens for genetic or molecular analysis in the current trial and for future use in ancillary studies, if applicable	10, main manuscript

*It is strongly recommended that this checklist be read in conjunction with the SPIRIT 2013 Explanation & Elaboration for important clarification on the items. Amendments to the protocol should be tracked and dated. The SPIRIT checklist is copyrighted by the SPIRIT Group under the Creative Commons "Attribution-NonCommercial-NoDerivs 3.0 Unported" license.

Confidential: For Review Only

Table 2: Balanced energy-protein supplement nutritional composition

Formula 1 ingredients: Vegetable oils (rapeseed, palm, soy in varying proportions), defatted soy flour, skimmed milk powder, peanuts, sugar, maltodextrin, soy protein isolate, vitamin and mineral complex, stabilizer (fully hydrogenated vegetable fat, mono and diglycerides). Allergens warning: peanuts, soy and dairy products. May contain traces of gluten.

Formula 2 ingredients: Vegetable oils (rapeseed, palm), skimmed milk powder, defatted soy flour, peanuts, sugar, maltodextrin, vitamin and mineral premix, fully hydrogenated vegetable oil (palm). Allergens warning: peanuts, soy and dairy products. May contain traces of gluten.

Formula 3 ingredients: Peanuts, vegetable oil (rapeseed), skimmed milk powder, sugar, whey protein concentrate, maltodextrin, premix of vitamins and minerals, fully hydrogenated vegetable oil (palm), flavor. Allergens warning: peanuts and dairy products. May contain traces of soy.

		**Formula 1**		**Formula 2**		**Formula 3**	
		**RUSF P&L 72g**		**Reformulation 1 RUSF P&L 72g**		**Reformulation RUSF 2 P&L 72**	
**Nutrients**	**Unit**	Mean / 100g	Mean / dose 72g	Mean / 100g	Mean / dose 72g	Mean / 100g	Mean / dose 72g
**Energy**	**kcal**	546	393	544	391	557	401
**Protein**	**g**	20.2	14.5	20.4	14.7	20.2	14.5
**Lipids**	**g**	36	26	36	26	37.3	26.9
**Linoleic Acid**	**g**	5.4	3.9	5.4	3.9	6.2	4.5
**alpha-Linolenic Acid**	**g**	1.8	1.3	1.9	1.4	1.8	1.3
**Calcium**	**mg**	694	500	776	559	695	500
**Zinc**	**mg**	21	15	21	15	21	15
**Copper**	**mg**	1.8	1.3	1.8	1.3	1.8	1.3

Confidential: For Review Only

**Iron**	**mg**	31	22	30	22	31	22
**Iodine**	**ug**	347	250	360	260	348	250
**Selenium**	**ug**	90	65	91	65	94	68
**Manganese**	**mg**	2.9	2.1	2.9	2.1	2.9	21
**Magnesium**	**mg**	102	73	108	78	99	72
**Free phosphorus**	**mg**	580	418	638	459	664	478
**Potassium**	**mg**	781	562	892	642	800	576
**Vitamin A**	**ug RE**	1069	770	1069	770	1,069	770
**Vitamin B1**	**mg**	1.9	1.4	1.9	1.4	1.9	1.4
**Vitamin B2**	**mg**	1.9	1.4	1.9	1.4	1.9	1.4
**Niacin**	**mg**	21	15	21	15	21	15
**Vitamin B5**	**mg**	9.7	7	9.7	7	9.7	7.0
**Vitamin B6**	**mg**	2.6	1.9	2.6	1.9	2.6	1.9
**Folic Acid**	**ug**	556	400	556	400	556	400
**Vitamin B12**	**ug**	3.6	2.6	3.6	2.6	3.6	2.6
**Vitamin C**	**mg**	139	100	139	100	139	100
**Vitamin D (Cholecacifero l)**	**ug**	21	15	21	15	21	15

Confidential: For Review Only

**Vitamin E (alpha tocopherol)**	**mg**	25	18	25	18	25	16
**Vitamin K**	**ug**	100	72	100	72	100	72

Confidential: For Review Only

Table 3: Summary of sub-study enrollments

This study has three sub-cohorts: two biospecimen cohorts and a neurodevelopment sub-study cohort.

	**1st biospecimen sub- study (n=100 women/infant pairs)**	**2nd biospecimen sub- study (n=120 study women/infant pairs)**	**Neurodevelopment sub-study (n=400 infants) women/infant pairs)**
**Hemoglobin (early and late pregnancy)**	100	120	N/A
**VAMS (early and late pregnancy)**	100	0	N/A
**Maternal stool (early and late pregnancy)**	100	0	N/A
**Infant stool**	100 (6 months)	120 (3, 6 months)	N/A
**Dietary 24 recall visit (2x visits in pregnancy and 3-months postpartum)**	100	120	N/A
**Neurodevelopment assessment visit at 6 months postnatal**	N/A	N/A	400

Confidential: For Review Only

Section A: Ultrasound gestational age dating protocol

Purpose: The purpose of the ultrasound exam is to collect data on fetal measurements using transabdominal ultrasound to estimate gestational age. Gestational age is required for calculation of our primary outcome, incidence of small-for-gestational-age (<10th centile) among live born infants whose birthweight is measured within 72 hours of delivery. Gestational age is also required construction of secondary outcomes, such as preterm birth.

Staff: The ultrasound gestational age exam is conducted by a specialized team of Auxiliary Nurse Midwives (ANMs) and assistants in this study. Previously, ANMs in our field team have undergone extensive training for in-home ultrasound examination to identify non-cephalic presentation, multiple births, placenta previa, and transabdominal ultrasound exam for gestational age dating. These trainings have been conducted by staff at the Department of Radiology at Tribhuvan University in Kathmandu. ANMs in this study receive similar training and oversight.

Measurements: The MINT study schedules participants for the ultrasound exam as soon as possible after identification of a pregnancy through our pregnancy surveillance system and the enrollment visit. The study aims to complete the ultrasound exam as earlier as feasible after pregnancy identification, ideally in the first trimester, for all participants to ensure the most accurate results. As part of the ultrasound exam, ANMs measure and record several measurements, depending upon the gestation at time of the exam, including crown rump length (CRL), biparietal diameter (BPD), head circumference (HC), and femur length (FL). The measurements and calculations follow the INTERGROWTH 21st methodology for early and late fetal gestational age assessment (Papageorghiou 2014, Papageorghiou 2016). In brief, the ANMs first attempt to measure CRL. If CRL is <15 mm, participants are rescheduled for another ultrasound exam in the following weeks. If CRL is ≥15 mm and <85 mm, the ANM will measure and record three CRL measurements. If CRL is ≥85 to ≤95 mm, in addition to CRL, the ANM also measure and record three values for BPD, HC, and FL. If CRL is >95 mm or CRL cannot be measured because the fetus is too large to fit in the field of view, the ANM will only measure and record three values for BPD, HC, and FL. Biparental diameter is measured “outer-to-outer.” The median measure for each anatomical feature is used for calculation of gestational age using the INTERGROWTH 21st formulas (Papageorghiou 2014, Papageorghiou 2016). Suspected abnormalities are recorded for review and referral by the study radiologist.

Quality control: ANMs record measurements and save digital images from the exam for review by senior field staff and a radiologist who are not present at the home visit. The radiologist reviews a 10-15% sample of images collected by the ANMs to validate that correct imaging and measurement techniques are used for each anatomical feature following the INTERGROWTH-21st Study Protocol (International Fetal and Newborn Growth Consortium 2009).

Equipment: We use the Sonosite NanoMaxx ultrasound machine for our study. The machine is FDA approved, and the specifications are as follows:

Confidential: For Review Only

- Product: Nanomaxx ultrasound machine
- Weight: 6 lbs / 2.7 kg (with battery)
- Dimensions: 14.1" L x 8.2" H x 2.3" W / 35.8 cm L x 20.8 cm H x 5.8 cm W
- Display: 8.4" / 21.3 cm diagonal display LCD touch screen
- Architecture: All-digital broadband
- Dynamic Range: Up to 165 dB
- Gray Scale: 200 dpi (voltage axis) x 500 dpi (time axis)
- HIPAA compliance: Comprehensive tool set
- Manufacturer: Sonosite

References:

Papageorghiou AT, Kennedy SH, Salomon LJ, Ohuma EO, Cheikh Ismail L, Barros FC, Lambert A, Carvalho M, Jaffer YA, Bertino E, Gravett MG, Altman DG, Purwar M, Noble JA, Pang R, Victora CG, Bhutta ZA, Villar J; International Fetal and Newborn Growth Consortium for the 21st Century (INTERGROWTH-21st). International standards for early fetal size and pregnancy dating based on ultrasound measurement of crown-rump length in the first trimester of pregnancy. Ultrasound Obstet Gynecol. 2014 Dec;44(6):641-8.

Papageorghiou AT, Kemp B, Stones W, Ohuma EO, Kennedy SH, Purwar M, Salomon LJ, Altman DG, Noble JA, Bertino E, Gravett MG, Pang R, Cheikh Ismail L, Barros FC, Lambert A, Jaffer YA, Victora CG, Bhutta ZA, Villar J; International Fetal and Newborn Growth Consortium for the 21st Century (INTERGROWTH-21st). Ultrasound-based gestational-age estimation in late pregnancy. Ultrasound Obstet Gynecol. 2016 Dec;48(6):719-726.

International Fetal and Newborn Growth Consortium. The International Fetal and Newborn Growth Standards for the 21st Century (INTERGROWTH-21st) Study Protocol, 2009, www.intergrowth21.org.uk

Confidential: For Review Only

Section B: Informed consent document

Nepal Pregnancy and Lactation Nutritional Supplementation Project

Consent for Participation in the Phase 3 Study

GREETING: Namaste! I am a Nepal Nutrition Intervention Project-Sarlahi (NNIPS) staff person. As you may know, NNIPS has been doing research projects concerning the health of mothers and children in your community for the past 30 years. Today, I would like to talk with you about a new NNIPS project. This project is being jointly run by NNIPS, Nepal Netra Jyoti Sangh, George Washington University, and Johns Hopkins University in the USA. I would like to tell you more this new project, ask for your consent to take part in it, and answer any questions you may have about the project.

PURPOSE

We are doing this new research study to learn more about nutrition for pregnant and breastfeeding women in our community. Specifically, we would like to learn if adding a special diet supplement (snack created to meet the needs of pregnant women in this area) to the diet of pregnant and breastfeeding women will improve the health and growth of their newborns and young babies. We would like to compare women who do and don't use this special diet supplement along with their normal diet. We are asking all pregnant women aged 15-30 in this community if they would like to participate in this study, until about 1800 women have agreed to be in the study. This study may help inform the government about ways to improve the health of babies and young infants in Nepal.

PROCEDURES

If you choose to take part in this project, I will ask you some questions today about your household and your family, and also ask about your previous pregnancies. I will also ask you about your current pregnancy, your health and diet, and measure your height, weight, and your blood pressure. This visit will take approximately 30-45 minutes. I will also schedule you to have an ultrasound examination by one of our Auxiliary Nurse-Midwives to check on how far along in pregnancy you are and to take a finger stick blood sample to measure your hemoglobin level. Once you are more than 14 weeks after your last period you will be asked to do one of 4 activities:

- Keep diet and level of activity as you normally would during your pregnancy and while you are breastfeeding.
- Take a special supplement to your diet while you are pregnant but then resume your regular diet after having your baby while you are breastfeeding.
- Keep your regular diet while you are pregnant, but take a special supplement to your diet during the first 6 months after having your baby, while you are breastfeeding.
- Take a special supplement to your diet while you are pregnant and after you have your baby for the first 6 months while you are breastfeeding.

You and the other pregnant women in your neighborhood will be assigned to one of these activities by chance. It is very important that you take the nutritional supplement every day if you are assigned to a supplement group and that you don't change your regular diet.

Confidential: For Review Only

I or another NNIPS worker will visit you to provide you with these special supplements and ask about your use of the supplements on a weekly basis. These visits will only take about 10 minutes.

Once late in your pregnancy, I or another NNIPS worker will visit to ask about your health during your pregnancy and to measure your weight and blood pressure.

We will visit you on the day your baby is born and ask some questions about the delivery and the health of you and your baby. We will also measure your baby's size and temperature. This visit will take about 30 minutes. We will continue to visit weekly until your baby is 6 months old. Once a month for 6 months, we will measure your baby's weight and length and your weight and ask some questions about the baby's and your health. These visits will take about 25 minutes. For some women and their infants, we will visit again when your baby is 12 months old to measure your baby's weight and length and your weight.

Altogether, you will be in this study for about 14 months.

RISKS/DISCOMFORTS

If you are allergic to milk, nuts (including peanuts) or soy you should not participate in this study. There are no significant risks to you or your baby if you take part in this study. There are minor risks to your privacy, but we will work hard to keep your information and participation confidential. You may feel uncomfortable with some of the questions we ask. If you feel at all uncomfortable with any of the questions we ask, you can just skip those questions. In the short interviews today and during the follow up visits, if there are any questions that are sensitive, you do not have to answer. You may experience slight pain in your finger when we take the blood sample.

As you know, proper nutrition in pregnancy is important for the health of babies in our community. While there are many potential benefits of better nutrition in pregnancy, it is possible that the nutritional supplement that we provide may not taste good to you.

BENEFITS

There are some benefits to participating in this study. We will provide you with a clean delivery kit, iron tablets, medicine for worms, and a baby blanket. We will also provide tetanus vaccine to you if you need it and have not received it at your routine antenatal care visit. We ask that you take the medicine for worms only after you are in your second trimester of pregnancy. The iron tablets, deworming medicine, and tetanus vaccine will improve the health of you during your pregnancy and will also improve the health of your growing baby.

All pregnant women and babies will be checked by our NNIPS workers, and while they cannot provide medical care, they can identify when your baby might be sick and can give you advice on when and where to seek care. The government may provide this care for free or you may have to pay some for it. We strongly encourage you to go to the local health center for antenatal care as well and to deliver your baby at an official birthing center.

We will tell you your hemoglobin level the day we take the finger stick blood sample. This will help determine the strength of your blood. If your hemoglobin level is too low, we will encourage you to take your iron tablets and, if needed, refer you to your local health center.

Confidential: For Review Only

The information we learn in this study may be helpful for current and future babies in your community.

CONFIDENTIALITY

All information you provide will remain secure with NNIPS staff and will not be shared with anyone who is not working in this project or those organizations who monitor research studies to be sure they are being done properly. We will share your information with the funding agency and other partners for this project but none of that information will identify you personally. We will keep all the forms and data in locked cabinets at our NNIPS office and in protected computer files. The information you provide will only be used for this research study.

With your permission, we want to keep your information in our files in case there will be another NNIPS study in the future.

VOLUNTARY PARTICIPATION

You have a choice to be in this project and you may say yes or no. If you do not wish to be in this project, your other option is to follow the diet you want to, and have routine care given to women in this area.

If you do agree to participate, you can also stop or withdraw from the study at any time. You will also be able to withdraw your permission for us to share your information with the funding agency and other partners for this project. This will not affect your access to health care or NNIPS activities now or in the future. All things provided to you in this study are free, but you will not be otherwise compensated for your participation.

PERSON TO CONTACT

If you have any questions or problems about the study, I can answer them now, or you can contact the Project Director, Dr Subarna Khatry at NNIPS office in Hariaun, Sarlahi (phone no. 046-530135). You can also contact the Nepal Health Research Council in Kathmandu if you have any concerns (phone no. 1-4227460).

You can also contact these people if you believe you have been injured by being in this study.

Do you give permission to participate in this study? Check: Yes _____ No _____

Do you give permission to share your information with the funding agency and other partners?

Check: Yes _______ No _________

Do you give permission to be contacted for future studies? Check: Yes ___ No ___

PERMISSION TO PROCEED

Confidential: For Review Only

Woman Consenting:

Name Signature Date

NNIPS Staff Witnessing Consent:

Name Signature Date

Husband's or Guardian's Consent for Women Younger than 18 yrs.:

Name Signature Date

Confidential: For Review Only

BMJ Paediatrics Open

Supplementation with fortified balanced energy-protein during pregnancy and lactation and its effects on birth outcomes and infant growth in southern Nepal: protocol of a 2x2 factorial randomized trial

Journal:	*BMJ Paediatrics Open*
Manuscript ID	bmjpo-2023-002229.R2
Article Type:	Protocol
Date Submitted by the Author:	25-Aug-2023

Complete List of Authors:	Erchick, Daniel; Johns Hopkins University Bloomberg School of Public Health, Department of International Health<br>Lama, Tsering P.; Johns Hopkins Bloomberg School of Public Health, Department of International Health; Nepal Nutrition Intervention Project Sarlahi (NNIPS)<br>Khatry, Subarna K.; Nepal Nutrition Intervention Project Sarlahi (NNIPS)<br>Katz, Joanne; Johns Hopkins Bloomberg School of Public Health, Department of International Health<br>Mullany, Luke C.; Johns Hopkins Bloomberg School of Public Health, Department of International Health<br>Zavala, Eleonor; Johns Hopkins Bloomberg School of Public Health, Department of International Health<br>LeClerq, Steven C.; Johns Hopkins Bloomberg School of Public Health, Department of International Health; Nepal Nutrition Intervention Project Sarlahi (NNIPS)<br>Christian, Parul; Johns Hopkins Bloomberg School of Public Health, Department of International Health<br>Tielsch, James M.; George Washington University, Department of Global Health

Keywords:	Epidemiology, Infant

SCHOLARONE™
Manuscripts

I, the Submitting Author has the right to grant and does grant on behalf of all authors of the Work (as defined in the below author licence), an exclusive licence and/or a non-exclusive licence for contributions from authors who are: i) UK Crown employees; ii) where BMJ has agreed a CC-BY licence shall apply, and/or iii) in accordance with the terms applicable for US Federal Government officers or employees acting as part of their official duties; on a worldwide, perpetual, irrevocable, royalty-free basis to BMJ Publishing Group Ltd ("BMJ") its licensees and where the relevant Journal is co-owned by BMJ to the co-owners of the Journal, to publish the Work in this journal and any other BMJ products and to exploit all rights, as set out in our licence.

The Submitting Author accepts and understands that any supply made under these terms is made by BMJ to the Submitting Author unless you are acting as an employee on behalf of your employer or a postgraduate student of an affiliated institution which is paying any applicable article publishing charge ("APC") for Open Access articles. Where the Submitting Author wishes to make the Work available on an Open Access basis (and intends to pay the relevant APC), the terms of reuse of such Open Access shall be governed by a Creative Commons licence – details of these licences and which Creative Commons licence will apply to this Work are set out in our licence referred to above.

Other than as permitted in any relevant BMJ Author's Self Archiving Policies, I confirm this Work has not been accepted for publication elsewhere, is not being considered for publication elsewhere and does not duplicate material already published. I confirm all authors consent to publication of this Work and authorise the granting of this licence.

Confidential: For Review Only

Supplementation with fortified balanced energy-protein during pregnancy and lactation and its effects on birth outcomes and infant growth in southern Nepal: protocol of a 2x2 factorial randomized trial

Daniel J. Erchick[1], Tsering P. Lama[1,2], Subarna K. Khatry[2], Joanne Katz[1], Luke C. Mullany[1], Eleonor Zavala[1], Steven C. LeClerq[1,2], Parul Christian[1], James M. Tielsch[3]

[1] Department of International Health, Johns Hopkins Bloomberg School of Public Health, Baltimore, MD, USA

[2] Nepal Nutrition Intervention Project Sarlahi (NNIPS), Kathmandu, Nepal

[3] Department of Global Health, Milken Institute School of Public Health, George Washington University, Washington, District of Columbia, USA

Corresponding author: Daniel Erchick, Department of International Health, Johns Hopkins Bloomberg School of Public Health, Baltimore, MD, USA. derchick@jhu.edu

Confidential: For Review Only

Abstract

Introduction: Many women in low and middle-income countries (LMICs) enter pregnancy with low nutritional reserves with increased risk for fetal growth restriction (FGR) and poor birth outcomes, including small-for-gestational-age (SGA) and preterm birth. Balanced energy-protein (BEP) supplements have shown reductions in risk of stillbirth and SGA, yet variations in intervention format and composition and limited evidence on the impact of BEP during lactation on growth outcomes, warrant further study. This paper describes the protocol of the Maternal Infant Nutrition Trial (MINT) study, which aims to evaluate the impact of a fortified BEP supplement during pregnancy and lactation on birth outcomes and infant growth in rural Nepal.

Methods and analysis: MINT is a 2x2 factorial, household randomized, unmasked, efficacy trial conducted in a sub-area of Sarlahi District, Nepal. The study area covers six rural municipalities with about 27,000 households and a population of approximately 100,000. Married women (15-30 years) who become pregnant are eligible for participation in the trial and are randomly assigned at enrollment to supplementation with fortified BEP or not and at birth to fortified BEP supplementation or not until 6 months postpartum. The primary pregnancy outcome is incidence of SGA, using the INTERGROWTH-21st standard, among live born infants with birth weight measured within 72 hours of delivery. The primary infant growth outcome is mean length-for-age z-score (LAZ) at 6 months using the WHO international growth reference.

Ethics and dissemination: The study was approved by Institutional Review Board (IRB) at Johns Hopkins Bloomberg School of Public Health, Baltimore, USA, (IRB00009714), the Committee on Human Research IRB at The George Washington University, Washington, DC, USA (081739), and the Ethical Review Board of the Nepal Health Research Council, Kathmandu, Nepal (174/2018).

Trial registration number: NCT03668977

Key words: Balanced energy-protein, maternal nutrition, small-for-gestational-age, pregnancy outcomes, infant growth, randomized trial, Nepal

Protocol version: Version 6, July 20, 2023

Confidential: For Review Only

KEY MESSAGES

What is already known on this topic: The World Health Organization recommendations on antenatal care include balanced energy-protein (BEP) supplementation in undernourished populations to reduce the risk of stillbirth and SGA birth.

What this study hopes to add: This study will demonstrate whether fortified BEP reduces risk of adverse pregnancy outcomes beyond multiple micronutrient supplementation. MINT will also generate evidence whether maternal intervention prevents early life growth faltering and improves maternal outcomes, such as anemia and inadequate gestational weight gain.

How this study might affect research, practice or policy: The MINT trial will help refine and implement the WHO context-specific recommendation on BEP supplementation in pregnancy and provide evidence for potential intervention in pregnancy and postpartum on infant growth outcomes.

Confidential: For Review Only

INTRODUCTION

Many women in low and middle-income countries (LMICs) enter pregnancy with low nutritional reserves, unable to meet the increased requirements for protein, energy, and micronutrients during pregnancy and lactation. Undernourished women are at increased risk of fetal growth restriction (FGR) and adverse birth outcomes, including preterm birth (<37 gestational weeks) and small-for-gestational age (SGA) (birth weight <10th percentile of a reference standard by gestational age and sex), two underlying biologic causes of low birth weight (LBW) (<2.5 kg).[1] SGA infants are more likely to experience higher mortality, linear growth faltering and poor health outcomes, poor cognitive performance, and diminished educational and employment attainment.[2-4] In 2020, 23.4 million infants (17.4% of all births) were born SGA, with the highest prevalence in South Asia (40.9%).[5, 6] Estimates from 2012 indicate that 22% neonatal deaths in LMICs, and 26% in South Asia, were attributable to SGA.[7, 8]

Infant size status at birth and growth in the first 6-months of life, especially among preterm and SGA infants, are critical determinants of childhood stunting and wasting associated with higher risk for mortality, morbidity, and poor developmental outcomes.[9] Many maternal risk factors associated with pregnancy outcomes and consequent preterm or SGA are likely contributors to linear growth failure.[10] Postnatal factors, such as breastfeeding and feeding behaviors, infections, and environmental factors, are also important drivers of infant growth.[11, 12]

Nutritional interventions targeted to women during pregnancy and lactation provide an opportunity to break the intergenerational cycle of growth failure. The World Health Organization (WHO) recommendations on antenatal care (ANC) include balanced energy-protein (BEP) supplementation in undernourished populations to reduce the risk of stillbirth and SGA birth.[13] This is based on a systematic review of BEP supplementation during pregnancy that has shown decreased risk of stillbirth (relative risk (RR): 0.60, 95% confidence interval (CI): 0.39 to 0.94) and SGA (RR: 0.79, 95% CI: 0.69 to 0.90),.[14] BEP also increased mean infant birth weight by 41g overall and 100g among undernourished women.[14, 15]

Despite this context-specific recommendation, the variation in formats, ingredients, and nutrient composition have hindered programmatic progress. Further, there is no recommendation for supplementation of women who are breastfeeding, despite high rates of growth faltering in early life in LMICs, where maternal undernutrition is high and exclusive breastfeeding is recommended in the first 6 months of life. Recently, an expert consensus developed specifications for a fortified BEP "ready-to-use" food supplement for pregnant and lactating women in LMICs.[16] Such a product requires testing in undernourished settings for efficacy in improving birth outcomes and infant growth.

In this paper, we describe the study protocol for the Maternal Infant Nutrition Trial (MINT) at the Nepal Nutrition Intervention Project Sarlahi (NNIPS) research site in Sarlahi District, Nepal. The aim of the MINT study is to evaluate the efficacy of daily fortified BEP supplementation during pregnancy and lactation on SGA and mean length-for-age z-score (LAZ) in the first six months of life. In this rural population, there is a significant burden of undernutrition among mothers and infants and high prevalence of adverse pregnancy outcomes.[17-19] However, breastfeeding

Confidential: For Review Only

through 6 months of life is high, presenting postpartum supplementation as a plausible mechanism by which to improve infant growth. Our primary hypotheses are that 1) fortified BEP supplementation vs. no supplementation during pregnancy will reduce incidence of SGA, and 2) fortified BEP supplementation vs. no supplementation during lactation alone or both during pregnancy and lactation will improve infant linear growth in the first six months after delivery. Several secondary outcomes will also be examined.

Confidential: For Review Only

METHODS AND ANALYSIS

Study design

MINT is a 2x2 factorial, household randomized, unmasked, efficacy trial of fortified BEP supplementation vs. no supplementation among women during pregnancy and lactation on SGA and infant growth to 6 months after delivery in rural Nepal (Figure 1). This community-based trial began in November 2021, and will enroll approximately 1,800 mother-fetus/infant pairs over the course of about 20 months.

We nested several sub-studies within the trial to explore the impact of the intervention on maternal dietary intake during pregnancy and postpartum, infant neurodevelopment at 6 months after delivery, metabolites in blood during pregnancy, and the gut microbiota among mothers in pregnancy and infants in the postnatal period.

This protocol follows the Standard Protocol Items: Recommendations for Interventional Trials (SPIRIT) guidelines (Supplementary Material, Table 1).[20]

Study setting

The MINT study is conducted in Sarlahi District, Madhesh Province, in the Terai region (low lying plains) of Nepal by the NNIPS research team. The NNIPS field research site, established in 1989 and covering approximately one-third of the district, currently operates as a joint undertaking between the Department of International Health, Johns Hopkins Bloomberg School of Public Health (JHSPH), and the nonprofit organization, Nepal Netra Jyoti Sangh, under a memorandum of agreement with the Social Welfare Council, Government of Nepal. Nepal's Terai region has high rates of maternal undernutrition, neonatal mortality, adverse pregnancy outcomes, and inadequate infant growth, and is representative of rural, low-income settings in much of South Asia.[21] Over half (56.9%) of households in Nepal's Madhesh Province faced food insecurity with 10% being severely food insecure, according to the Nepal Demographic and Health Survey 2016.[21] Of children under five years old, 29.3% were stunted and 10.1% were wasted in Madhesh Province in 2022.[22] Data from our most recent trial in the NNIPS study area, which had over 31,000 live births (2010-2017), reported neonatal mortality and stillbirth rates of approximately 32 per 1,000 live births and 29 per 1,000 births respectively (unpublished). In that trial, the proportion of adverse pregnancy outcomes were: preterm birth 14.9%, SGA 48.5%, and LBW 30.1% (unpublished). Nearly a third of women in their first trimester of pregnancy were underweight (29.5%) (body mass index (BMI) <18.5 kg/m^2) and the same proportion (29.5%) had a height less than 145 cm (unpublished). Over a third of infants (35%) are breastfed within an hour of birth and 95% within 24 hours in our trial population (unpublished).

Study population and recruitment

The study area includes 34 wards selected from six rural municipalities (Haripur, Ishwarpur, Kabilasi, Malangawa, Chakraghatta and Chandranagar) with a population of approximately 100,000 people. MINT enumerated and recruited participants through a household census and

Confidential: For Review Only

pregnancy surveillance system. Due to disruptions caused by the COVID-19 pandemic, the trial census and enrollment was divided into two periods.

In July 2019, we conducted a census to enumerate the households in the study area with potentially eligible women. The census activity identified about 27,000 total households, including roughly 13,000 households with at least one woman meeting the eligibility criteria for pregnancy surveillance. The first enrollment period spanned November 2021 to June 2022, enrolling 938 women.

Between the original census and the start of pregnancy enrollment (which was delayed due to the COVID-19 pandemic), many newly married women accumulated in the study area. In December 2022 and January 2023, we conducted a new household census update activity to add these newly married women, who would be eligible for enrollment in the trial, to our census and pregnancy surveillance roster. The census update activity added about 1,400 newly married women to the census roster for a total of around 14,400 women eligible for pregnancy surveillance and subsequent enrollment if they become pregnant. The second enrollment period began in January 2023 and will run until the sample size of 1,800 women is met.

Pregnancy surveillance is conducted by local, female data collectors hired on the project to make household visits every 5 weeks to ask potential participants if they had a period since the last visit and offer a urine-based pregnancy test if not. All married women (15-30 years) resident in the study area at the time of the census who are identified as pregnant by our pregnancy surveillance system (<24 weeks gestation) and consent to participate are eligible for participation in the trial. Women who self-report an allergy to nuts, milk, or soy at enrollment are excluded.

We have enrolled 1,944 women as of August 2023.

Randomization/allocation

The study uses a household-randomization scheme as there are joint families in which more than one woman could contribute a pregnancy, which would increase risk of sharing/crossover in the context of individual randomization. At enrollment, randomization is stratified by maternal height (<150 cm, ≥150 cm). Using the median of three height measures of the first woman enrolled in the house, we assign each household to either supplementation or not during pregnancy within each height category. We assign any additional women who became pregnant in a household that had already been randomized to the same group. At the birth visit, we randomize within each height category and pregnancy allocation group, assigning each household to either supplementation or not during the postpartum period. Only households with at least one live birth are eligible for postpartum randomization and follow-up. Since the likelihood of more than one woman from the same household becoming pregnant within the enrollment period is estimated to be low, at ~96.5% from prior data , this choice of randomization approach allows us to gain the statistical strength of an individually randomized trial.

https://mc.manuscriptcentral.com/bmjpo
Confidential: For Review Only

We pre-generated randomization sequences using the R software environment for statistical programming (www.r-project.org). We generated these sequences using randomly permuted blocks of size 4, 6, 8, and 12, to ensure sequential (i.e., chronological) balance across the groups throughout the enrollment period.

Masking of participants or study data collectors was not feasible due to the nature of the intervention and control.

Intervention

At enrollment, households are randomly allocated to either supplementation or not for the duration of pregnancy. Women in the supplementation group receive at home a supply of fortified BEP supplement for daily consumption beginning at 14 gestational weeks until the pregnancy outcome. Both groups also receive i) recommendation to enroll in antenatal care (ANC) at a local health clinic and deliver at a certified birthing facility, ii) nutrition, hygiene, and breast feeding and infant care counseling, and iii) a clean birthing kit. In pregnancy, women in both groups are provided iron-folic acid tablets and albendazole, if not provided via antenatal care at their health facility. At the birth visit, households where a woman reports at least one live birth are randomly allocated to either supplementation or not for the postpartum period until the infant is 6 months old. Counseling for exclusive breast feeding is provided in the postpartum period but iron-folic acid tablets are not provided as per the policy in Nepal.

Composition of fortified BEP supplement

The fortified BEP supplement is a ready-to-eat snack in the form of lipid-based peanut paste packaged in individual sachets (72g). The supplement was designed to meet the expert consensus specifications of a nutritious food supplement for pregnant and lactating women.[16] Each sachet is a daily portion that provides calories (~400 kcal), protein (~14 g), and multiple micronutrients at the estimated average requirement for pregnancy. The product is made by Nutriset, France, as a specially-manufactured version of their product known as “Plumpy’Mum”. We previously conducted two formative research studies to evaluate the acceptability of and adherence to various flavors of the BEP product in this community.[23, 24] Due to unavailability of raw materials, Nutriset produced three variations of Plumpy’Mum for use in our study, each with different combinations of ingredients selected to produce a product with a similar nutritional composition. The changes to the original ingredients were to remove the soy isolate (first change) and soy flour (second change), which resulted in minor changes in energy, protein, and micronutrient concentrations, deemed inconsequential from a nutrition perspective. The specific ingredients and nutritional composition of each variation are provided in the Supplementary Material, Table 2.

Outcomes

The study has two primary outcomes, one each for the pregnancy and postpartum/postnatal periods, respectively. The primary outcome for the pregnancy period is incidence of SGA among live born infants with birth weight measured within 72 hours of delivery. SGA is defined as weight measured within <72 h of delivery <10th centile of the INTERGROWTH 21st reference standard by gestational age and sex.[25] We assessed gestational age using 1st or

Confidential: For Review Only

2nd trimester ultrasound examination prior to 24 gestational weeks. The primary outcome for the postpartum/postnatal period is mean LAZ at 6 months using the international WHO child growth reference.[26] Secondary outcomes are listed in Table 1.

Sample size

With a sample size of 1,548 woman/infant pairs visited within <72 hours of delivery, we will be able to detect a 17.5% reduction in SGA with 80% power and a 20.2% reduction with 90% power assuming a type 1 error of 5% (two-sided). Since we are assuming no interaction between the marginal effects, this sample size will allow us to detect a difference in infant LAZ at 6 months after delivery of 0.16 Z scores with 80% power and 0.18 Z scores with 90% power. The MINT trial is designed to enroll approximately 1,800-2,000 women over a period of 20 months (split into two 6-month enrollment periods). This will yield the required sample of 1,548 woman/infant pairs visited within 72 hours of delivery, after a reduction of 14% due to fetal death and loss to follow-up in pregnancy.

Data collection

All study visits and interviews are conducted in participant households because of the wide dispersion of households across this rural community and the impracticality of bringing participants to a central location (Figure 2). Data collection teams comprising Field Interviewers (FIs) schedule the study visits, administer the questionnaires, and conduct the assessments as described below.

Enrollment visit: FIs conduct the enrollment visit, after notification of a newly identified pregnancy, to administer informed consent, baseline questionnaires, and anthropometry. Questionnaires cover maternal demographic characteristics, socioeconomic status, pregnancy and reproductive history, dietary diversity, and household food security (Household Food Insecurity Access Scale (HFIAS) developed by the Food and Nutrition Technical Assistance).[27] Anthropometric measurements include height (portable stadiometer), weight (MicroLife WS50 scale), mid-upper arm circumference (MUAC) (non-stretch insertion tape), blood pressure, and pulse.

Ultrasound visit: A special cadre of Auxiliary Nurse Midwives (ANMs) conduct the ultrasound assessment for gestational dating by visiting the participant's homes (Supplementary Material, Section A). ANMs are trained to use a portable machine (SonoSite NanoMaxx) and follow the INTERGROWTH 21st methodology for early and late fetal gestational age assessment, including measurement of crown-rump length and/or biparietal diameter (outer-to-outer), head circumference, and femur length.[28, 29]

Weekly morbidity and supplement compliance visits: Data collectors visit each woman weekly during pregnancy starting at 14 weeks based on LMP and in the postpartum period until 6 months after delivery to provide the supplement, collect data on maternal morbidities, and assess supplement compliance.

https://mc.manuscriptcentral.com/bmjpo
Confidential: For Review Only

Late pregnancy visit: FIs conduct a late pregnancy visit between 32-34 gestational weeks to collect data on maternal anthropometry (same measures as enrollment), maternal morbidities, tobacco/alcohol use, ANC utilization, delivery planning, diet diversity, household food security, and depression (using a version of the Edinburgh Postnatal Depression Scale validated in Nepali).[30]

Birth assessment visit: FIs aim to visit each woman within 72 hours of delivery to collection information about the birth and delivery process, early breastfeeding and feeding behaviors, and newborn care practices. Infant weight (g), length (cm), head circumference (cm), and temperature (F) are measured. Weight is measured with digital scales (Tanita BD- 585), length with Shorr-type boards, and MUAC with non-stretch insertion tape. Non-digital measures are taken in triplicate and the median is used for analysis.

Postpartum/Postnatal monthly visits: After the birth visit, field interviewers conduct monthly postnatal visits up to 6 months after delivery to administer questionnaires on maternal and infant morbidity, breastfeeding and infant and young child feeding practices, infant care practices, household food security, and anthropometric measurements for the infant (weight, length, head circumference, MUAC) and mother (weight, MUAC). Additionally, we will visit as many woman/infant pairs at 12 months after delivery as possible until the administrative end of the study to allow us to assess infant growth at 12 months as a secondary aim in a sub-sample.

Biospecimen sub-studies: The MINT study will enroll two sub-studies for biospecimen collection (Supplementary Material, Table 3). First, we will enroll about 120 pregnant women during the first enrollment period from the pregnancy supplementation (n=60) and not groups (n=60). At the enrollment and late pregnancy visits, these women will provide volumetric absorptive microsampling (VAM) samples, for serum metabolomics analyses collected via finger-stick using Neoteryx kits, hemoglobin measurement, a maternal stool sample stored in nucleic acid buffer solution (DNA Genotek OMR-GUT), and their infants will provide a stool sample at 6 months. Second, we will enroll about 120 pregnant women during the second enrollment period from the pregnancy supplementation (n=60) and not groups (n=60), and these women will provide a hemoglobin measurement at the enrollment and late pregnancy visits, and their infants will provide a stool sample at 3 and 6 months.

Dietary intake: Field interviewers will conduct duplicate 24-hour dietary recalls at the late pregnancy and 3-months postpartum visits for a subset of 240 women in the first (n=120) and second (n=120) enrollment periods. The purpose of these data is to assess nutrient intakes and determine whether dietary substitution is occurring in the supplement group. Interviewers use the multiple pass method to help the woman recall all the components of her diet from the previous day. A local food list, photo atlas, and electronic entry form have been adapted from previous work in Nepal to assist with portion size recall and data entry.[31, 32] Daily nutrient intakes will be estimated with local recipes and national food composition tables from India and Nepal.

Confidential: For Review Only

Bayley Scales of Infant and Toddler development (BSID-4) visit: A specialized team with experience and training in developmental assessment in children will visit a sub-sample of 400 mother/infant pairs to evaluate infant neurodevelopment at 6 months of life.[33, 34] The visit will include the BSID-4 assessment, a standardized evaluation for infants that measures functioning in five areas: cognition, fine motor, gross motor, receptive language, and expressive language. At this visit, data collectors will also conduct the World Health Organization six gross motor milestones module.[35]

Data management

Our field interviewers administer study questionnaires and record anthropometric measurements via digital tablets with a cellular connection using a customized web-based data management system developed on the Microsoft ASP.NET framework. The local data collectors complete paper forms that are recorded by data entry operators on a weekly basis. On the backend, data are stored in a relational database management system (Microsoft SQL Server). Prior to study launch, the field team tests all forms, and restrictions and checks are programmed to reduce data entry errors. The senior field team and co-investigators review important variables weekly to monitor data quality issues, such as missingness, heaping, digit preference, and outliers. The senior field team corrects data entry errors and implements retraining of data collectors on specific protocols when necessary.

Data monitoring

Data and safety monitoring board: An independent Data and Safety Monitoring Board (DSMB) is established for the trial to advise the investigators on issues of safety and efficacy. DSMB membership includes four individuals from Nepal, including a pediatrician, obstetrician-gynecologist, nutritionist, and statistician. The DSMB met prior to the start of the study, twice during implementation of the study, and will meet at least once more.

Serious adverse events: Serious adverse events, as defined under Good Clinical Practice (GCP) guidelines, include death, a life-threatening reaction to the supplement or other study procedure, hospitalization for a reason other than routine delivery, significant or persistent disability or impairment, or a congenital anomaly/birth defect. All data collection staff are trained to recognize a range of serious and other adverse events (AEs). SAEs are investigated and reported to the DSMB and IRBs according to the study research and ethical protocols. Our most senior data collectors conduct verbal autopsies in the case of a maternal or infant death.

Statistical analysis

Primary aim 1: We will undertake an intention-to-treat analysis to examine the effect of supplementation on the first primary outcome of SGA. The analysis will include all live births for whom a birth weight was collected by study data collectors within 72 hours of delivery for the SGA (<10th centile births using the INTERGROWTH-21st standard) outcome. We will assess the balance between the two pregnancy supplementation groups in baseline household- and individual-level characteristics including maternal age, height, body mass index (BMI), parity, previous adverse pregnancy outcome, education, and socioeconomic status. If imbalanced variables are also associated with the primary outcome ($p<0.10$), we will use multivariable log

Confidential: For Review Only

binomial regression modeling to adjust for potential confounding. We will compare the proportion of SGA, between those who were supplemented vs. not according to their allocation status, using a log binomial regression model to estimate the relative risk and 95% confidence interval with the control group as the referent both unadjusted and adjusted for identified confounders. If the final model does not converge, we will use Poisson regression with robust standard error estimation.

Primary aim 2: We will undertake an intention-to-treat analysis to examine the effect of supplementation on the second primary outcome of mean LAZ at 6 months. The analysis will include all children whose length was assessed at 6 months of age. We will assess the balance across the four pregnancy and lactation supplementation groups in baseline household- and individual-level characteristics. If imbalanced variables are also associated with the secondary outcome ($p<0.10$), we will use multivariable linear regression modeling to adjust for potential confounding. Due to the factorial study design, we will assess the interaction between pregnancy and lactation supplementation on the outcome of mean LAZ, although our sample size is not adequate to detect small interaction effects. We will use a multivariable linear regression model with an interaction term for treatment arms. If the interaction is significant (<0.10), we will report both the main and interaction effects. If not significant, which we anticipate, we will report the marginal treatment effects for supplementation during lactation vs. not and during pregnancy vs. not. We will compare mean LAZ scores between supplementation groups using a multivariable linear regression model to estimate the risk difference and 95% confidence interval with the control group as the referent both unadjusted and adjusted for identified confounders.

ETHICS AND DISSEMINATION

Ethical approval

The study was approved by Institutional Review Board (IRB) at Johns Hopkins Bloomberg School of Public Health, Baltimore, USA, (IRB00009714), the Committee on Human Research IRB at The George Washington University, Washington, DC, USA (081739), and the Ethical Review Board of the Nepal Health Research Council, Kathmandu, Nepal (174/2018).

Patient and public involvement

We conducted two formative studies to assess compliance with and acceptability of various formulations of BEP supplements. Our field leadership team also engaged several local stakeholders prior to the start of the study, including municipality mayors, health coordinators, and health facility in-charges in the study area. After completion of the study, the team will conduct similar meetings to disseminate the results.

Ancillary care

The MINT study provides iron folic acid tablets and albendazole to women who report that they have not received these from the health facility during their ANC visits. Women with low hemoglobin (Hb <11g/dL) are referred to a health facility. Women with suspected fetal

Confidential: For Review Only

abnormality detected during the ultrasound gestational age dating visit are referred for clinical examination.

Confidentiality

Study investigators and staff treat all data as confidential, and participants are informed that their information will be protected. All data collection forms are kept in secure locations with access limited to relevant study staff. All electronic databases are stored on encrypted, password-protected servers, and data transfer uses secure encrypted transfer protocols. Access to identifying information is restricted to the principal investigator and few key co-investigators and staff. Only anonymized datasets are shared beyond the study team.

Dissemination plan

The study team will disseminate MINT findings to the Sarlahi District Public Health Office, Sarahi District Project Advisory Committee, Ministry of Health and Population, Nepal, Central Project Advisory Committee, and to various local organizations, including Unicef-Nepal and Unicef South Asian Regional Office, USAID, and other stakeholders in Nepal with interests in nutrition during pregnancy and lactation. We will share anonymized data with the Ki data repository and researchers involved in the BEP Harmonization Initiative organized and funded by the BMGF for the purpose of meta-analyses using data from similar trials conducted around the world. The study team will also present results at international workshops and conferences with relevant researchers and program or policy stakeholders. Members of the study team will publish results of primary and secondary analyses in peer-reviewed academic journals.

Confidential: For Review Only

DISCUSSION

Nepal has made progress in reducing maternal, newborn, and child mortality, but a significant burden of undernutrition remains among mothers and infants.[21, 22] Novel and effective interventions and practical implementation strategies are needed to prevent adverse pregnancy outcomes and infant growth faltering,[36, 37] and currently WHO (2016)[13] recommends use of BEP supplements in undernourished settings, but standard formulations of such interventions and data on implementation in programs is lacking. The MINT trial will provide evidence to help refine and implement the WHO context-specific recommendation on BEP supplementation in pregnancy, especially using the fortified, enhanced ready-to-use formulation recommended by the expert consensus on BEP supplements.[16] As multiple micronutrient supplementation (MMS) reduces risk of adverse pregnancy outcomes, including low birth weight and SGA, our trial will contribute evidence as to whether the fortified BEP intervention, which include the same 15 micronutrients, provides additional benefit beyond MMS alone.[38] This trial will generate evidence on the potential of a maternal intervention to prevent early life growth faltering and improve maternal outcomes, such as anemia and inadequate gestational weight gain, areas where a research gap exists. The results from the trial will inform health policy and programs in Nepal, where there currently are no guidelines for community-level management of growth faltering in infants less than 6 months of age beyond exclusive breastfeeding. The primary strength of the trial is the 2x2 factorial RCT design, which will enable understanding of the interconnections between maternal nutritional status in pregnancy and lactation and relationships to fetal and infant growth faltering. The trial includes frequent follow-up visits, over the continuum from early pregnancy to 6 months after delivery, which will generate data on many prenatal and postpartum factors that influence maternal and infant outcomes. The trial will describe maternal dietary intake, assess whether provision of BEP supplements lead to dietary substitution, and measure adherence, yielding practical insights for implementation. Lastly, the trial structure allows for nesting various sub-studies, including those to assess the impact of BEP on the maternal and infant microbiome, infant neurodevelopment, and maternal metabolites.

Confidential: For Review Only

Figure legend

Figure 1: MINT study design

Figure 2: MINT study visits and data collection

Acknowledgements

We express our sincere thanks to the women and their families in Sarlahi, District, Nepal, who participated in the MINT study. Thank you to Nutriset, France, for producing and donating a portion of the fortified BEP supplements. This study was conducted in collaboration with our implementing partner, Nepal Netra Jyoti Sangh, under the auspices of the Social Welfare Council of the Government of Nepal.

Competing interests

The authors declare no competing interests.

Authors' contributions

JT, JK, LM, and SKK conceptualized the study. All authors contributed to development of the study design, protocols, and forms and tools. SKK, TPL, and SCL led data collection and supervision in the field. DE wrote the first draft of the manuscript. All authors provided revisions and inputs to the manuscript. All authors reviewed and agreed on the final version of the manuscript.

Data availability

The principal investigator will share final anonymized data with the Ki data repository and researchers involved in the BEP Harmonization Initiative for the purpose of meta-analyses using data from similar trials conducted around the world. Data will also be available to other investigators upon reasonable request.

Funding

This study was supported by the Bill & Melinda Gates Foundation (OPP11599195), Thrasher Research Fund (TRF01278), and the Eunice Kennedy Shriver National Institute of Child Health and Human Development (R01HD109385). The funders had no role in the design, implementation, or analysis of the trial.

Confidential: For Review Only

Table 1: MINT study primary and secondary outcomes

**Primary outcomes**	
Small-for-gestational age (10th centile)	Proportion of infants with birth weight <10th centile of INTERGROWTH 21st reference standard by gestational age and sex for infants measured <72 hours after delivery.
Length-for-age Z (LAZ)	Mean infant length-for-age Z score at 6 months of age using the World Health Organization Child Growth Study criteria.
**Secondary outcomes**	
**Newborn**	
Small-for-gestational-age (3rd centile)	Proportion of infants with birth weight <3rd centile of INTERGROWTH 21st reference standard by gestational age and sex for infants measured <72 hours after delivery.
Large-for-gestational-age (90th centile)	Proportion of infants with birth weight >90th centile of INTERGROWTH 21st reference standard by gestational age and sex for infants measured <72 hours after delivery.
Short-for-gestational-age (10th centile)	Proportion of infants with birth length <10th centile of INTERGROWTH 21st reference standard by gestational age and sex for infants measured <72 hours after delivery.
Short-for-gestational-age (3rd centile)	Proportion of infants with birth length <3rd centile of INTERGROWTH 21st reference standard by gestational age and sex for infants measured <72 hours after delivery.
Birth weight	Mean infant birth weight.
Birth length	Mean infant birth length.
Head circumference	Mean infant head circumference
Arm circumference	Mean infant mid-upper arm circumference
Low birth weight	Proportion of infants with birth weight <2,500g for infants measured <72 hours after delivery.
Gestational age	Mean infant gestational age at delivery.
Preterm birth (<37 weeks)	Proportion of pregnancies that are <37 gestational weeks using the INTERGROWTH 21st standards for 1st and 2nd trimester ultrasound dating.
Very, extremely preterm (<34, <32, <28 weeks)	Proportion of pregnancies that are <34, <32, <28 gestational weeks using the INTERGROWTH 21st standards for 1st and 2nd trimester ultrasound dating.
Stillbirth	Proportion of pregnancies resulting in stillbirth, defined as fetal death ≥28 gestational weeks, using the INTERGROWTH 21st standards for 1st and 2nd trimester ultrasound dating.

Confidential: For Review Only

Weight-for-length Z score at 6 months of age	Mean infant weight-for-length Z score at 6 months of age using the World Health Organization Child Growth Study criteria.
Weight-for-age Z score at 6 months of age	Mean infant weight-for-age Z score at 6 months of age using the World Health Organization Child Growth Study criteria.
Stunting	Proportion of infants with <-2 length-for-age Z score at 6 months of age using the World Health Organization Child Growth Study criteria.
Wasting	Proportion of infants with <-2 weight-for-length Z score at 6 months of age using the World Health Organization Child Growth Study criteria.
Underweight	Proportion of infants with <-2 weight-for-age Z score at 6 months of age using the World Health Organization Child Growth Study criteria.
Developmental scales	Bayley Scales of Infant and Toddler Development 4 (BSID-4) assessment at 6 months.
Gross motor milestones	World Health Organization 6 gross motor milestones at 6 months.
Gut microbiome	Gut microbiome diversity and stool metabolomics at 3, 6 months.
**Maternal**	
Gestational weight gain	Mean maternal weight gain from enrollment to late pregnancy visits.
Net gestational weight gain	Mean maternal weight gain from enrollment to maternal birth assessment visit (after delivery).
Inadequate maternal gestational weight gain	Proportion of women with inadequate rate of gestational weight gain from enrollment to late pregnancy visit using the INTERGROWTH 21st reference for normal body mass index (BMI) and IOM reference for low and high BMI.
Inadequate maternal net gestational weight gain	Proportion of women with inadequate rate of gestational weight gain from enrollment to maternal birth assessment visit using the INTERGROWTH 21st reference for normal body mass index (BMI) and IOM reference for low and high BMI.
Mid-upper arm circumference	Mean change in mid-upper arm circumference from enrollment to late pregnancy visits.
Anemia	Proportion of women who are anemic (Hb <110 g/L) in late pregnancy.
Weight at 6 months postpartum	Mean maternal weight at 6 months postpartum.
Maternal dietary intake	Mean maternal dietary intake score in late pregnancy and 3 months postpartum.
Minimum maternal dietary intake	Proportion of women meeting the minimum dietary intake score in late pregnancy and 3 months postpartum.

Confidential: For Review Only

Gut microbiome	Gut microbiome diversity and stool metabolomics during pregnancy.
Metabolites	Metabolites in the blood during pregnancy.

Confidential: For Review Only

References

1. Kozuki N, Katz J, Lee AC, et al. Short Maternal Stature Increases Risk of Small-for-Gestational-Age and Preterm Births in Low- and Middle-Income Countries: Individual Participant Data Meta-Analysis and Population Attributable Fraction. J Nutr. 2015;145(11):2542-50.
2. Christian P, Lee SE, Donahue Angel M, et al. Risk of childhood undernutrition related to small-for-gestational age and preterm birth in low- and middle-income countries. Int J Epidemiol. 2013;42(5):1340-55.
3. Murray E, Fernandes M, Fazel M, et al. Differential effect of intrauterine growth restriction on childhood neurodevelopment: a systematic review. Bjog. 2015;122(8):1062-72.
4. Victora CG, Adair L, Fall C, et al. Maternal and child undernutrition: consequences for adult health and human capital. Lancet. 2008;371(9609):340-57.
5. Lawn J, Ohuma E, Bradley E, et al. Small babies, big risks: Global estimates of prevalence and mortality for vulnerable newborns to accelerate change and improve counting. Lancet. 2023.
6. Erchick DJ, Hazel EA, Katz J, et al. Vulnerable newborn types: analysis of subnational, population-based birth cohorts for 541 285 live births in 23 countries, 2000-2021. Bjog. 2023.
7. Katz J, Lee AC, Kozuki N, et al. Mortality risk in preterm and small-for-gestational-age infants in low-income and middle-income countries: a pooled country analysis. Lancet. 2013;382(9890):417-25.
8. Lee AC, Kozuki N, Cousens S, et al. Estimates of burden and consequences of infants born small for gestational age in low and middle income countries with INTERGROWTH-21(st) standard: analysis of CHERG datasets. Bmj. 2017;358:j3677.
9. Benjamin-Chung J, Mertens A, Colford JM, et al. Early childhood linear growth failure in low- and middle-income countries. medRxiv. 2020:2020.06.09.20127001.
10. Christian P. Prenatal origins of malnutrition. Emerging Societies Coexistence of Childhood Malnutrition and Obesity. 632009.
11. Black RE, Victora CG, Walker SP, et al. Maternal and child undernutrition and overweight in low-income and middle-income countries. Lancet. 2013;382(9890):427-51.
12. Keusch GT, Rosenberg IH, Denno DM, et al. Implications of acquired environmental enteric dysfunction for growth and stunting in infants and children living in low- and middle-income countries. Food Nutr Bull. 2013;34(3):357-64.
13. World Health Organization. WHO Guidelines Approved by the Guidelines Review Committee. WHO Recommendations on Antenatal Care for a Positive Pregnancy Experience. Geneva: World Health Organization; 2016.
14. Ota E, Hori H, Mori R, et al. Antenatal dietary education and supplementation to increase energy and protein intake. Cochrane Database Syst Rev. 2015(6):Cd000032.
15. Imdad A, Bhutta ZA. Maternal nutrition and birth outcomes: effect of balanced protein-energy supplementation. Paediatr Perinat Epidemiol. 2012;26 Suppl 1:178-90.
16. Members of an Expert Consultation on Nutritious Food Supplements for Pregnant and Lactating Women. Framework and specifications for the nutritional composition of a food supplement for pregnant and lactating women (PLW) in undernourished and low income settings. . Gates Open Res. 2019;3:1498 (document)
17. Subedi S, Hazel EA, Mohan D, et al. Prevalence and predictors of spontaneous preterm births in Nepal: findings from a prospective, population-based pregnancy cohort in rural Nepal-a secondary data analysis. BMJ Open. 2022;12(12):e066934.
18. Bryce E, Gurung S, Tong H, et al. Population attributable fractions for risk factors for spontaneous preterm births in 81 low- and middle-income countries: A systematic analysis. J Glob Health. 2022;12:04013.

Confidential: For Review Only

19. Hazel EA, Mohan D, Zeger S, et al. Demographic, socio-economic, obstetric, and behavioral factors associated with small-and large-for-gestational-age from a prospective, population-based pregnancy cohort in rural Nepal: a secondary data analysis. BMC Pregnancy Childbirth. 2022;22(1):652.
20. Chan AW, Tetzlaff JM, Altman DG, et al. SPIRIT 2013 statement: defining standard protocol items for clinical trials. Ann Intern Med. 2013;158(3):200-7.
21. Ministry of Health - MOH/Nepal, New ERA/Nepal, ICF. Nepal Demographic and Health Survey 2016. Kathmandu, Nepal: MOH/Nepal, New ERA, and ICF; 2017.
22. Ministry of Health - MOH/Nepal, New ERA/Nepal, ICF. Nepal Demographic and Health Survey Key Indicators Report 2022. Kathmandu, Nepal: MOH/Nepal, NEW ERA, and ICF; 2022.
23. Lama TP, Khatry SK, Isanaka S, et al. Acceptability of 11 fortified balanced energy-protein supplements for pregnant women in Nepal. Matern Child Nutr. 2022;18(3):e13336.
24. Lama TP, Moore K, Isanaka S, et al. Compliance with and acceptability of two fortified balanced energy protein supplements among pregnant women in rural Nepal. Matern Child Nutr. 2022;18(2):e13306.
25. Villar J, Cheikh Ismail L, Victora CG, et al. International standards for newborn weight, length, and head circumference by gestational age and sex: the Newborn Cross-Sectional Study of the INTERGROWTH-21st Project. Lancet. 2014;384(9946):857-68.
26. WHO Child Growth Standards based on length/height, weight and age. Acta Paediatr Suppl. 2006;450:76-85.
27. Coates J, Swindale, A, Bilinsky P. Household Food Insecurity Access Scale (HFIAS) for Measurement of Household Food Access: Indicator Guide (v. 3). Washington, D.C.: FHI 360/FANTA; 2007.
28. Papageorghiou AT, Kennedy SH, Salomon LJ, et al. International standards for early fetal size and pregnancy dating based on ultrasound measurement of crown–rump length in the first trimester of pregnancy. Ultrasound in Obstetrics & Gynecology. 2014;44(6):641-8.
29. Papageorghiou AT, Kemp B, Stones W, et al. Ultrasound-based gestational-age estimation in late pregnancy. Ultrasound Obstet Gynecol. 2016;48(6):719-26.
30. Bhusal BR, Bhandari N, Chapagai M, et al. Validating the Edinburgh Postnatal Depression Scale as a screening tool for postpartum depression in Kathmandu, Nepal. Int J Ment Health Syst. 2016;10:71-.
31. Harris-Fry H, Paudel P, Karn M, et al. Development and validation of a photographic food atlas for portion size assessment in the southern plains of Nepal. Public Health Nutr. 2016;19(14):2495-507.
32. Harris-Fry H, Beard BJ, Harrisson T, et al. Smartphone tool to collect repeated 24 h dietary recall data in Nepal. Public Health Nutr. 2018;21(2):260-72.
33. Murray-Kolb LE, Rasmussen ZA, Scharf RJ, et al. The MAL-ED cohort study: methods and lessons learned when assessing early child development and caregiving mediators in infants and young children in 8 low- and middle-income countries. Clin Infect Dis. 2014;59 Suppl 4(Suppl 4):S261-72.
34. Ranjitkar S, Kvestad I, Strand TA, et al. Acceptability and Reliability of the Bayley Scales of Infant and Toddler Development-III Among Children in Bhaktapur, Nepal. Front Psychol. 2018;9:1265.
35. Wijnhoven TM, de Onis M, Onyango AW, et al. Assessment of gross motor development in the WHO Multicentre Growth Reference Study. Food Nutr Bull. 2004;25(1 Suppl):S37-45.
36. Bhutta ZA, Das JK, Rizvi A, et al. Evidence-based interventions for improvement of maternal and child nutrition: what can be done and at what cost? Lancet. 2013;382(9890):452-77.
37. Bhutta ZA, Das JK, Bahl R, et al. Can available interventions end preventable deaths in mothers, newborn babies, and stillbirths, and at what cost? Lancet. 2014;384(9940):347-70.

Confidential: For Review Only

38. Keats EC, Haider BA, Tam E, et al. Multiple-micronutrient supplementation for women during pregnancy. Cochrane Database Syst Rev. 2019;3(3):Cd004905.

Confidential: For Review Only

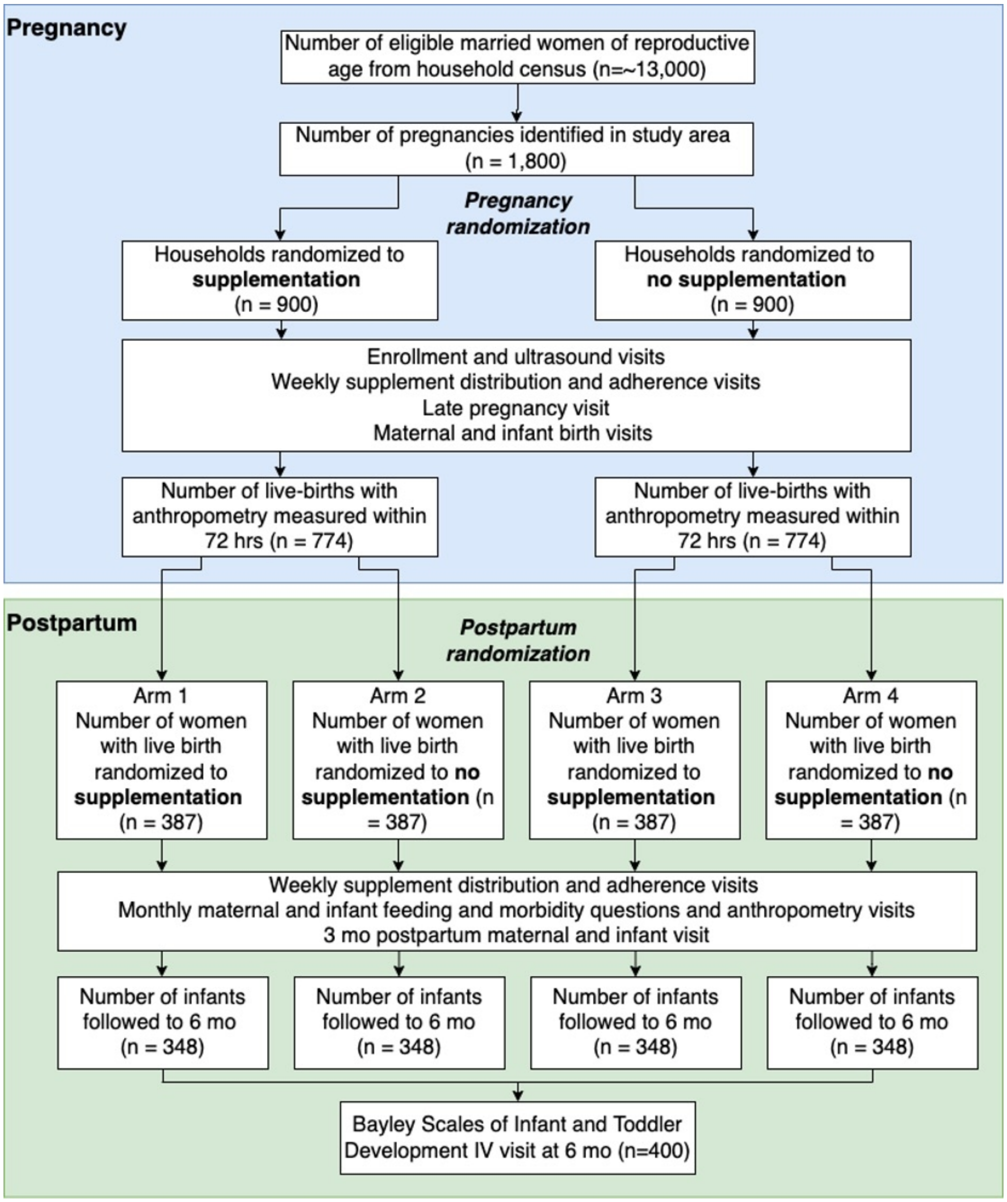

Figure 1: Study design of MINT 2x2 factorial randomized trial

165x197mm (144 x 144 DPI)

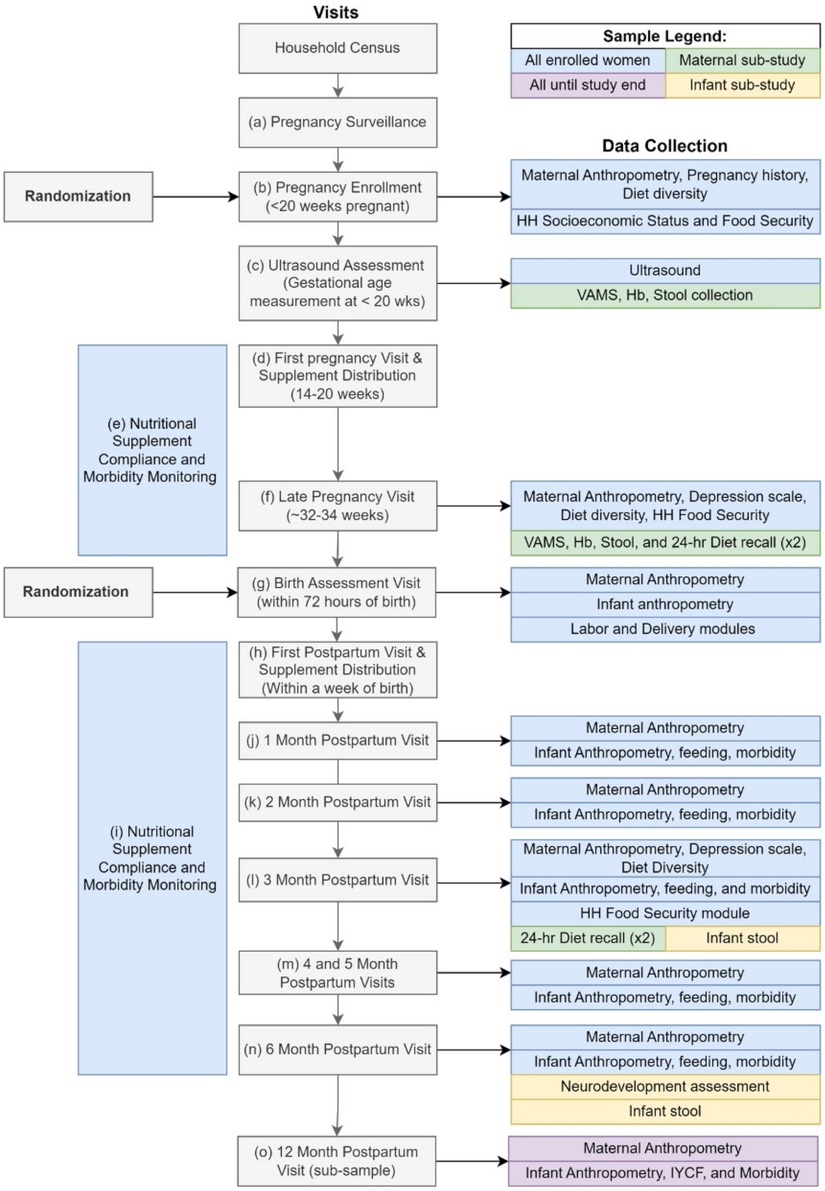

151x220mm (144 x 144 DPI)

Confidential: For Review Only

SUPPLEMENTARY MATERIAL

Table 1: Standard protocol items: recommendations for interventional trials (SPIRIT) guidelines checklist

SPIRIT 2013 Checklist: Recommended items to address in a clinical trial protocol and related documents*

**Section/item**	**Item No**	**Description**	**Addressed on page number**
**Administrative information**			
Title	1	Descriptive title identifying the study design, population, interventions, and, if applicable, trial acronym	_____1______
Trial registration	2a	Trial identifier and registry name. If not yet registered, name of intended registry	_____2______
	2b	All items from the World Health Organization Trial Registration Data Set	_____2______
Protocol version	3	Date and version identifier	_____2______
Funding	4	Sources and types of financial, material, and other support	____15______
Roles and responsibilities	5a	Names, affiliations, and roles of protocol contributors	_____1______
	5b	Name and contact information for the trial sponsor	_____15_____

	5c	Role of study sponsor and funders, if any, in study design; collection, management, analysis, and interpretation of data; writing of the report; and the decision to submit the report for publication, including whether they will have ultimate authority over any of these activities	_____15______
	5d	Composition, roles, and responsibilities of the coordinating centre, steering committee, endpoint adjudication committee, data management team, and other individuals or groups overseeing the trial, if applicable (see Item 21a for data monitoring committee)	______NA______

Introduction

Background and rationale	6a	Description of research question and justification for undertaking the trial, including summary of relevant studies (published and unpublished) examining benefits and harms for each intervention	______4______
	6b	Explanation for choice of comparators	______4______
Objectives	7	Specific objectives or hypotheses	_____4-5______
Trial design	8	Description of trial design including type of trial (eg, parallel group, crossover, factorial, single group), allocation ratio, and framework (eg, superiority, equivalence, noninferiority, exploratory)	_____4-5______

Confidential: For Review Only

Methods: Participants, interventions, and outcomes

Study setting	9	Description of study settings (eg, community clinic, academic hospital) and list of countries where data will be collected. Reference to where list of study sites can be obtained	_____6______
Eligibility criteria	10	Inclusion and exclusion criteria for participants. If applicable, eligibility criteria for study centres and individuals who will perform the interventions (eg, surgeons, psychotherapists)	_____7______
Interventions	11a	Interventions for each group with sufficient detail to allow replication, including how and when they will be administered	_____8______
	11b	Criteria for discontinuing or modifying allocated interventions for a given trial participant (eg, drug dose change in response to harms, participant request, or improving/worsening disease)	_____8______
	11c	Strategies to improve adherence to intervention protocols, and any procedures for monitoring adherence (eg, drug tablet return, laboratory tests)	____9-10_____
	11d	Relevant concomitant care and interventions that are permitted or prohibited during the trial	_____8______

Outcomes	12	Primary, secondary, and other outcomes, including the specific measurement variable (eg, systolic blood pressure), analysis metric (eg, change from baseline, final value, time to event), method of aggregation (eg, median, proportion), and time point for each outcome. Explanation of the clinical relevance of chosen efficacy and harm outcomes is strongly recommended	_____8______
Participant timeline	13	Time schedule of enrolment, interventions (including any run-ins and washouts), assessments, and visits for participants. A schematic diagram is highly recommended (see Figure)	__9-10, 16____
Sample size	14	Estimated number of participants needed to achieve study objectives and how it was determined, including clinical and statistical assumptions supporting any sample size calculations	____8-9______
Recruitment	15	Strategies for achieving adequate participant enrolment to reach target sample size	____6-7______

Methods: Assignment of interventions (for controlled trials)

Allocation:

ly

Sequence generation	16a	Method of generating the allocation sequence (eg, computer-generated random numbers), and list of any factors for stratification. To reduce predictability of a random sequence, details of any planned restriction (eg, blocking) should be provided in a separate document that is unavailable to those who enrol participants or assign interventions	____7-8______
Allocation concealment mechanism	16b	Mechanism of implementing the allocation sequence (eg, central telephone; sequentially numbered, opaque, sealed envelopes), describing any steps to conceal the sequence until interventions are assigned	____7-8______
Implementation	16c	Who will generate the allocation sequence, who will enrol participants, and who will assign participants to interventions	____7-8______
Blinding (masking)	17a	Who will be blinded after assignment to interventions (eg, trial participants, care providers, outcome assessors, data analysts), and how	_____8______
	17b	If blinded, circumstances under which unblinding is permissible, and procedure for revealing a participant's allocated intervention during the trial	____NA______

Methods: Data collection, management, and analysis

Data collection methods	18a	Plans for assessment and collection of outcome, baseline, and other trial data, including any related processes to promote data quality (eg, duplicate measurements, training of assessors) and a description of study instruments (eg, questionnaires, laboratory tests) along with their reliability and validity, if known. Reference to where data collection forms can be found, if not in the protocol	___9-10_____
	18b	Plans to promote participant retention and complete follow-up, including list of any outcome data to be collected for participants who discontinue or deviate from intervention protocols	___9-10______
Data management	19	Plans for data entry, coding, security, and storage, including any related processes to promote data quality (eg, double data entry; range checks for data values). Reference to where details of data management procedures can be found, if not in the protocol	_____11_____
Statistical methods	20a	Statistical methods for analysing primary and secondary outcomes. Reference to where other details of the statistical analysis plan can be found, if not in the protocol	____11-12____
	20b	Methods for any additional analyses (eg, subgroup and adjusted analyses)	___11-12_____
	20c	Definition of analysis population relating to protocol non-adherence (eg, as randomised analysis), and any statistical methods to handle missing data (eg, multiple imputation)	___11-12_____

Methods: Monitoring

Data monitoring	21a	Composition of data monitoring committee (DMC); summary of its role and reporting structure; statement of whether it is independent from the sponsor and competing interests; and reference to where further details about its charter can be found, if not in the protocol. Alternatively, an explanation of why a DMC is not needed	____11______
	21b	Description of any interim analyses and stopping guidelines, including who will have access to these interim results and make the final decision to terminate the trial	____NA______
Harms	22	Plans for collecting, assessing, reporting, and managing solicited and spontaneously reported adverse events and other unintended effects of trial interventions or trial conduct	____11______
Auditing	23	Frequency and procedures for auditing trial conduct, if any, and whether the process will be independent from investigators and the sponsor	____NA______

Ethics and dissemination

Research ethics approval	24	Plans for seeking research ethics committee/institutional review board (REC/IRB) approval	____12______

w Only

Protocol amendments	25	Plans for communicating important protocol modifications (eg, changes to eligibility criteria, outcomes, analyses) to relevant parties (eg, investigators, REC/IRBs, trial participants, trial registries, journals, regulators)	____12______
Consent or assent	26a	Who will obtain informed consent or assent from potential trial participants or authorised surrogates, and how (see Item 32)	_____9______
	26b	Additional consent provisions for collection and use of participant data and biological specimens in ancillary studies, if applicable	_____NA_____
Confidentiality	27	How personal information about potential and enrolled participants will be collected, shared, and maintained in order to protect confidentiality before, during, and after the trial	___12-13_____
Declaration of interests	28	Financial and other competing interests for principal investigators for the overall trial and each study site	_____15_____
Access to data	29	Statement of who will have access to the final trial dataset, and disclosure of contractual agreements that limit such access for investigators	_____15_____
Ancillary and post-trial care	30	Provisions, if any, for ancillary and post-trial care, and for compensation to those who suffer harm from trial participation	_____NA_____

Confidential: For Revi

Dissemination policy	31a	Plans for investigators and sponsor to communicate trial results to participants, healthcare professionals, the public, and other relevant groups (eg, via publication, reporting in results databases, or other data sharing arrangements), including any publication restrictions	______13_____
	31b	Authorship eligibility guidelines and any intended use of professional writers	______15_____
	31c	Plans, if any, for granting public access to the full protocol, participant-level dataset, and statistical code	______15_____
**Appendices**			
Informed consent materials	32	Model consent form and other related documentation given to participants and authorised surrogates	__Section C___
Biological specimens	33	Plans for collection, laboratory evaluation, and storage of biological specimens for genetic or molecular analysis in the current trial and for future use in ancillary studies, if applicable	10, main manuscript

*It is strongly recommended that this checklist be read in conjunction with the SPIRIT 2013 Explanation & Elaboration for important clarification on the items. Amendments to the protocol should be tracked and dated. The SPIRIT checklist is copyrighted by the SPIRIT Group under the Creative Commons "Attribution-NonCommercial-NoDerivs 3.0 Unported" license.

Confidential: For Review Only

Table 2: Balanced energy-protein supplement nutritional composition

Formula 1 ingredients: Vegetable oils (rapeseed, palm, soy in varying proportions), defatted soy flour, skimmed milk powder, peanuts, sugar, maltodextrin, soy protein isolate, vitamin and mineral complex, stabilizer (fully hydrogenated vegetable fat, mono and diglycerides). Allergens warning: peanuts, soy and dairy products. May contain traces of gluten.

Formula 2 ingredients: Vegetable oils (rapeseed, palm), skimmed milk powder, defatted soy flour, peanuts, sugar, maltodextrin, vitamin and mineral premix, fully hydrogenated vegetable oil (palm). Allergens warning: peanuts, soy and dairy products. May contain traces of gluten.

Formula 3 ingredients: Peanuts, vegetable oil (rapeseed), skimmed milk powder, sugar, whey protein concentrate, maltodextrin, premix of vitamins and minerals, fully hydrogenated vegetable oil (palm), flavor. Allergens warning: peanuts and dairy products. May contain traces of soy.

		**Formula 1**		**Formula 2**		**Formula 3**	
		**RUSF P&L 72g**		**Reformulation 1 RUSF P&L 72g**		**Reformulation RUSF 2 P&L 72**	
**Nutrients**	**Unit**	Mean / 100g	Mean / dose 72g	Mean / 100g	Mean / dose 72g	Mean / 100g	Mean / dose 72g
**Energy**	**kcal**	546	393	544	391	557	401
**Protein**	**g**	20.2	14.5	20.4	14.7	20.2	14.5
**Lipids**	**g**	36	26	36	26	37.3	26.9
**Linoleic Acid**	**g**	5.4	3.9	5.4	3.9	6.2	4.5
**alpha-Linolenic Acid**	**g**	1.8	1.3	1.9	1.4	1.8	1.3
**Calcium**	**mg**	694	500	776	559	695	500
**Zinc**	**mg**	21	15	21	15	21	15
**Copper**	**mg**	1.8	1.3	1.8	1.3	1.8	1.3

Confidential: For Review Only

**Iron**	**mg**	31	22	30	22	31	22
**Iodine**	**ug**	347	250	360	260	348	250
**Selenium**	**ug**	90	65	91	65	94	68
**Manganese**	**mg**	2.9	2.1	2.9	2.1	2.9	21
**Magnesium**	**mg**	102	73	108	78	99	72
**Free phosphorus**	**mg**	580	418	638	459	664	478
**Potassium**	**mg**	781	562	892	642	800	576
**Vitamin A**	**ug RE**	1069	770	1069	770	1,069	770
**Vitamin B1**	**mg**	1.9	1.4	1.9	1.4	1.9	1.4
**Vitamin B2**	**mg**	1.9	1.4	1.9	1.4	1.9	1.4
**Niacin**	**mg**	21	15	21	15	21	15
**Vitamin B5**	**mg**	9.7	7	9.7	7	9.7	7.0
**Vitamin B6**	**mg**	2.6	1.9	2.6	1.9	2.6	1.9
**Folic Acid**	**ug**	556	400	556	400	556	400
**Vitamin B12**	**ug**	3.6	2.6	3.6	2.6	3.6	2.6
**Vitamin C**	**mg**	139	100	139	100	139	100
**Vitamin D (Cholecacifero l)**	**ug**	21	15	21	15	21	15

Confidential: For Review Only

**Vitamin E (alpha tocopherol)**	**mg**	25	18	25	18	25	16
**Vitamin K**	**ug**	100	72	100	72	100	72

Confidential: For Review Only

Table 3: Summary of sub-study enrollments
This study has three sub-cohorts: two biospecimen cohorts and a neurodevelopment sub-study cohort.

	**1st biospecimen sub- study (n=100 women/infant pairs)**	**2nd biospecimen sub- study (n=120 study women/infant pairs)**	**Neurodevelopment sub-study (n=400 infants) women/infant pairs)**
**Hemoglobin (early and late pregnancy)**	100	120	N/A
**VAMS (early and late pregnancy)**	100	0	N/A
**Maternal stool (early and late pregnancy)**	100	0	N/A
**Infant stool**	100 (6 months)	120 (3, 6 months)	N/A
**Dietary 24 recall visit (2x visits in pregnancy and 3-months postpartum)**	100	120	N/A
**Neurodevelopment assessment visit at 6 months postnatal**	N/A	N/A	400

Confidential: For Review Only

Section A: Ultrasound gestational age dating protocol

Purpose: The purpose of the ultrasound exam is to collect data on fetal measurements using transabdominal ultrasound to estimate gestational age. Gestational age is required for calculation of our primary outcome, incidence of small-for-gestational-age (<10th centile) among live born infants whose birthweight is measured within 72 hours of delivery. Gestational age is also required construction of secondary outcomes, such as preterm birth.

Staff: The ultrasound gestational age exam is conducted by a specialized team of Auxiliary Nurse Midwives (ANMs) and assistants in this study. Previously, ANMs in our field team have undergone extensive training for in-home ultrasound examination to identify non-cephalic presentation, multiple births, placenta previa, and transabdominal ultrasound exam for gestational age dating. These trainings have been conducted by staff at the Department of Radiology at Tribhuvan University in Kathmandu. ANMs in this study receive similar training and oversight.

Measurements: The MINT study schedules participants for the ultrasound exam as soon as possible after identification of a pregnancy through our pregnancy surveillance system and the enrollment visit. The study aims to complete the ultrasound exam as earlier as feasible after pregnancy identification, ideally in the first trimester, for all participants to ensure the most accurate results. As part of the ultrasound exam, ANMs measure and record several measurements, depending upon the gestation at time of the exam, including crown rump length (CRL), biparietal diameter (BPD), head circumference (HC), and femur length (FL). The measurements and calculations follow the INTERGROWTH 21st methodology for early and late fetal gestational age assessment (Papageorghiou 2014, Papageorghiou 2016). In brief, the ANMs first attempt to measure CRL. If CRL is <15 mm, participants are rescheduled for another ultrasound exam in the following weeks. If CRL is ≥15 mm and <85 mm, the ANM will measure and record three CRL measurements. If CRL is ≥85 to ≤95 mm, in addition to CRL, the ANM also measure and record three values for BPD, HC, and FL. If CRL is >95 mm or CRL cannot be measured because the fetus is too large to fit in the field of view, the ANM will only measure and record three values for BPD, HC, and FL. Biparental diameter is measured "outer-to-outer." The median measure for each anatomical feature is used for calculation of gestational age using the INTERGROWTH 21st formulas (Papageorghiou 2014, Papageorghiou 2016). Suspected abnormalities are recorded for review and referral by the study radiologist.

Quality control: ANMs record measurements and save digital images from the exam for review by senior field staff and a radiologist who are not present at the home visit. The radiologist reviews a 10-15% sample of images collected by the ANMs to validate that correct imaging and measurement techniques are used for each anatomical feature following the INTERGROWTH-21st Study Protocol (International Fetal and Newborn Growth Consortium 2009).

Equipment: We use the Sonosite NanoMaxx ultrasound machine for our study. The machine is FDA approved, and the specifications are as follows:

Confidential: For Review Only

- Product: Nanomaxx ultrasound machine
- Weight: 6 lbs / 2.7 kg (with battery)
- Dimensions: 14.1" L x 8.2" H x 2.3" W / 35.8 cm L x 20.8 cm H x 5.8 cm W
- Display: 8.4" / 21.3 cm diagonal display LCD touch screen
- Architecture: All-digital broadband
- Dynamic Range: Up to 165 dB
- Gray Scale: 200 dpi (voltage axis) x 500 dpi (time axis)
- HIPAA compliance: Comprehensive tool set
- Manufacturer: Sonosite

References:

Papageorghiou AT, Kennedy SH, Salomon LJ, Ohuma EO, Cheikh Ismail L, Barros FC, Lambert A, Carvalho M, Jaffer YA, Bertino E, Gravett MG, Altman DG, Purwar M, Noble JA, Pang R, Victora CG, Bhutta ZA, Villar J; International Fetal and Newborn Growth Consortium for the 21st Century (INTERGROWTH-21st). International standards for early fetal size and pregnancy dating based on ultrasound measurement of crown-rump length in the first trimester of pregnancy. Ultrasound Obstet Gynecol. 2014 Dec;44(6):641-8.

Papageorghiou AT, Kemp B, Stones W, Ohuma EO, Kennedy SH, Purwar M, Salomon LJ, Altman DG, Noble JA, Bertino E, Gravett MG, Pang R, Cheikh Ismail L, Barros FC, Lambert A, Jaffer YA, Victora CG, Bhutta ZA, Villar J; International Fetal and Newborn Growth Consortium for the 21st Century (INTERGROWTH-21st). Ultrasound-based gestational-age estimation in late pregnancy. Ultrasound Obstet Gynecol. 2016 Dec;48(6):719-726.

International Fetal and Newborn Growth Consortium. The International Fetal and Newborn Growth Standards for the 21st Century (INTERGROWTH-21st) Study Protocol, 2009, www.intergrowth21.org.uk

Confidential: For Review Only

Section B: Informed consent document

Nepal Pregnancy and Lactation Nutritional Supplementation Project

Consent for Participation in the Phase 3 Study

GREETING: Namaste! I am a Nepal Nutrition Intervention Project-Sarlahi (NNIPS) staff person. As you may know, NNIPS has been doing research projects concerning the health of mothers and children in your community for the past 30 years. Today, I would like to talk with you about a new NNIPS project. This project is being jointly run by NNIPS, Nepal Netra Jyoti Sangh, George Washington University, and Johns Hopkins University in the USA. I would like to tell you more this new project, ask for your consent to take part in it, and answer any questions you may have about the project.

PURPOSE

We are doing this new research study to learn more about nutrition for pregnant and breastfeeding women in our community. Specifically, we would like to learn if adding a special diet supplement (snack created to meet the needs of pregnant women in this area) to the diet of pregnant and breastfeeding women will improve the health and growth of their newborns and young babies. We would like to compare women who do and don't use this special diet supplement along with their normal diet. We are asking all pregnant women aged 15-30 in this community if they would like to participate in this study, until about 1800 women have agreed to be in the study. This study may help inform the government about ways to improve the health of babies and young infants in Nepal.

PROCEDURES

If you choose to take part in this project, I will ask you some questions today about your household and your family, and also ask about your previous pregnancies. I will also ask you about your current pregnancy, your health and diet, and measure your height, weight, and your blood pressure. This visit will take approximately 30-45 minutes. I will also schedule you to have an ultrasound examination by one of our Auxiliary Nurse-Midwives to check on how far along in pregnancy you are and to take a finger stick blood sample to measure your hemoglobin level. Once you are more than 14 weeks after your last period you will be asked to do one of 4 activities:

- Keep diet and level of activity as you normally would during your pregnancy and while you are breastfeeding.
- Take a special supplement to your diet while you are pregnant but then resume your regular diet after having your baby while you are breastfeeding.
- Keep your regular diet while you are pregnant, but take a special supplement to your diet during the first 6 months after having your baby, while you are breastfeeding.
- Take a special supplement to your diet while you are pregnant and after you have your baby for the first 6 months while you are breastfeeding.

You and the other pregnant women in your neighborhood will be assigned to one of these activities by chance. It is very important that you take the nutritional supplement every day if you are assigned to a supplement group and that you don't change your regular diet.

Confidential: For Review Only

I or another NNIPS worker will visit you to provide you with these special supplements and ask about your use of the supplements on a weekly basis. These visits will only take about 10 minutes.

Once late in your pregnancy, I or another NNIPS worker will visit to ask about your health during your pregnancy and to measure your weight and blood pressure.

We will visit you on the day your baby is born and ask some questions about the delivery and the health of you and your baby. We will also measure your baby's size and temperature. This visit will take about 30 minutes. We will continue to visit weekly until your baby is 6 months old. Once a month for 6 months, we will measure your baby's weight and length and your weight and ask some questions about the baby's and your health. These visits will take about 25 minutes. For some women and their infants, we will visit again when your baby is 12 months old to measure your baby's weight and length and your weight.

Altogether, you will be in this study for about 14 months.

RISKS/DISCOMFORTS

If you are allergic to milk, nuts (including peanuts) or soy you should not participate in this study. There are no significant risks to you or your baby if you take part in this study. There are minor risks to your privacy, but we will work hard to keep your information and participation confidential. You may feel uncomfortable with some of the questions we ask. If you feel at all uncomfortable with any of the questions we ask, you can just skip those questions. In the short interviews today and during the follow up visits, if there are any questions that are sensitive, you do not have to answer. You may experience slight pain in your finger when we take the blood sample.

As you know, proper nutrition in pregnancy is important for the health of babies in our community. While there are many potential benefits of better nutrition in pregnancy, it is possible that the nutritional supplement that we provide may not taste good to you.

BENEFITS

There are some benefits to participating in this study. We will provide you with a clean delivery kit, iron tablets, medicine for worms, and a baby blanket. We will also provide tetanus vaccine to you if you need it and have not received it at your routine antenatal care visit. We ask that you take the medicine for worms only after you are in your second trimester of pregnancy. The iron tablets, deworming medicine, and tetanus vaccine will improve the health of you during your pregnancy and will also improve the health of your growing baby.

All pregnant women and babies will be checked by our NNIPS workers, and while they cannot provide medical care, they can identify when your baby might be sick and can give you advice on when and where to seek care. The government may provide this care for free or you may have to pay some for it. We strongly encourage you to go to the local health center for antenatal care as well and to deliver your baby at an official birthing center.

We will tell you your hemoglobin level the day we take the finger stick blood sample. This will help determine the strength of your blood. If your hemoglobin level is too low, we will encourage you to take your iron tablets and, if needed, refer you to your local health center.

Confidential: For Review Only

The information we learn in this study may be helpful for current and future babies in your community.

CONFIDENTIALITY

All information you provide will remain secure with NNIPS staff and will not be shared with anyone who is not working in this project or those organizations who monitor research studies to be sure they are being done properly. We will share your information with the funding agency and other partners for this project but none of that information will identify you personally. We will keep all the forms and data in locked cabinets at our NNIPS office and in protected computer files. The information you provide will only be used for this research study.

With your permission, we want to keep your information in our files in case there will be another NNIPS study in the future.

VOLUNTARY PARTICIPATION

You have a choice to be in this project and you may say yes or no. If you do not wish to be in this project, your other option is to follow the diet you want to, and have routine care given to women in this area.

If you do agree to participate, you can also stop or withdraw from the study at any time. You will also be able to withdraw your permission for us to share your information with the funding agency and other partners for this project. This will not affect your access to health care or NNIPS activities now or in the future. All things provided to you in this study are free, but you will not be otherwise compensated for your participation.

PERSON TO CONTACT

If you have any questions or problems about the study, I can answer them now, or you can contact the Project Director, Dr Subarna Khatry at NNIPS office in Hariaun, Sarlahi (phone no. 046-530135). You can also contact the Nepal Health Research Council in Kathmandu if you have any concerns (phone no. 1-4227460).

You can also contact these people if you believe you have been injured by being in this study.

Do you give permission to participate in this study? Check: Yes _____ No _____

Do you give permission to share your information with the funding agency and other partners?

Check: Yes _______ No _________

Do you give permission to be contacted for future studies? Check: Yes ___ No ___

PERMISSION TO PROCEED

Confidential: For Review Only

Woman Consenting:

Name Signature Date

NNIPS Staff Witnessing Consent:

Name Signature Date

Husband's or Guardian's Consent for Women Younger than 18 yrs.:

Name Signature Date

Confidential: For Review Only
