# Supplementary material for: Supplementation with fortified balanced energy–protein during pregnancy and lactation and its effects on birth outcomes and infant growth in southern Nepal: protocol of a 2×2 factorial randomised trial
Source: BMJ Paediatr Open. 2023 Nov 3;7(1):e002229. doi: 10.1136/bmjpo-2023-002229 (PMC10626787; doi:10.1136/bmjpo-2023-002229)
Supplement: Reviewer comments [file bmjpo-2023-002229.reviewer_comments.pdf]

PEER REVIEW HISTORY

BMJ Paediatrics Open publishes all reviews undertaken for accepted manuscripts. Reviewers are asked to complete a checklist review form and are provided with free text boxes to elaborate on their assessment. These free text comments are reproduced below.

ARTICLE DETAILS

**TITLE (PROVISIONAL)**	Supplementation with fortified balanced energy-protein during pregnancy and lactation and its effects on birth outcomes and infant growth in southern Nepal: protocol of a 2x2 factorial randomized trial
**AUTHORS**	Daniel J. Erchick, Tsering P. Lama, Subarna K. Khatry, Joanne Katz, Luke C. Mullany, Eleonor Zavala, Steven C. LeClerq, Parul Christian, James M. Tielsch

This article was not externally reviewed at BMJ Paediatrics Open. Protocols that have been independently assessed prior to submission to BMJ Paediatrics Open are usually fast-tracked to publication on the grounds that further substantial changes will not be possible. This independent assessment will usually be external, independent review for both a substantial grant award from a non-commercial or government funder and ethics approval.
